# Supplementary material for: Detection of interphylum transfers of the magnetosome gene cluster in magnetotactic bacteria
Source: Front Microbiol. 2022 Aug 1;13:945734. doi: 10.3389/fmicb.2022.945734 (PMC9376291; doi:10.3389/fmicb.2022.945734)
Supplement: Supplementary file 1 [file Data_Sheet_1.PDF]

frontiers

Supplementary information

Detection of interphylum transfers of the magnetosome gene cluster in magnetotactic bacteria

Maria Uzun[1,2,*], Veronika Koziaeva[1,*], Marina Dziuba[1,3], Pedro Leão[4,+], Maria Krutkina[5], Denis Grouzdev[5, #]

*authors with equal contribution

[1] Skryabin Institute of Bioengineering, Research Center of Biotechnology of the Russian Academy of Sciences, Moscow, Russia

[2] Faculty of Biology, Lomonosov Moscow State University, Moscow, Russia

[3] Department of Microbiology, University of Bayreuth, Bayreuth, Germany

[4] Instituto de Microbiologia Paulo de Góes, Universidade Federal do Rio de Janeiro, Rio de Janeiro, Brazil

[+] Current affiliation: Department of Marine Science, The University of Texas at Austin, Port Aransas, USA

[5] SciBear OU, Tallinn, Estonia

[#] corresponding author: denisgrouzdev@gmail.com

Content

Supplementary figure S1. Crystal size metrics for strain LBB01. 2

Supplementary figure S2. Phylogenomic analysis and reconciliation results for genomes from the *Nitrospirota* phylum including 36 MTB genomes. 3

Supplementary table S1. Statistics for all bacterial genomes used in this work 4

Supplementary table S2. Results of reconciliations for protein trees and concatenated protein tree obtained by Notung and Ranger-DTL tools. 16

Supplementary table S3. Reconstructed genomes statistics. 18

Supplementary table S4. AAI and POCP values between *Nitrospirota* genomes. 19

Supplementary table S5. ANI and DDH values between LBB01 and closely related genomes 20

Supplementary table S6. ANI and DDH values between LBB02 and closely related genomes 21

Supplementary table S7. Genomes used for the MGC genes search in the *Nitrospirota* phylum 22

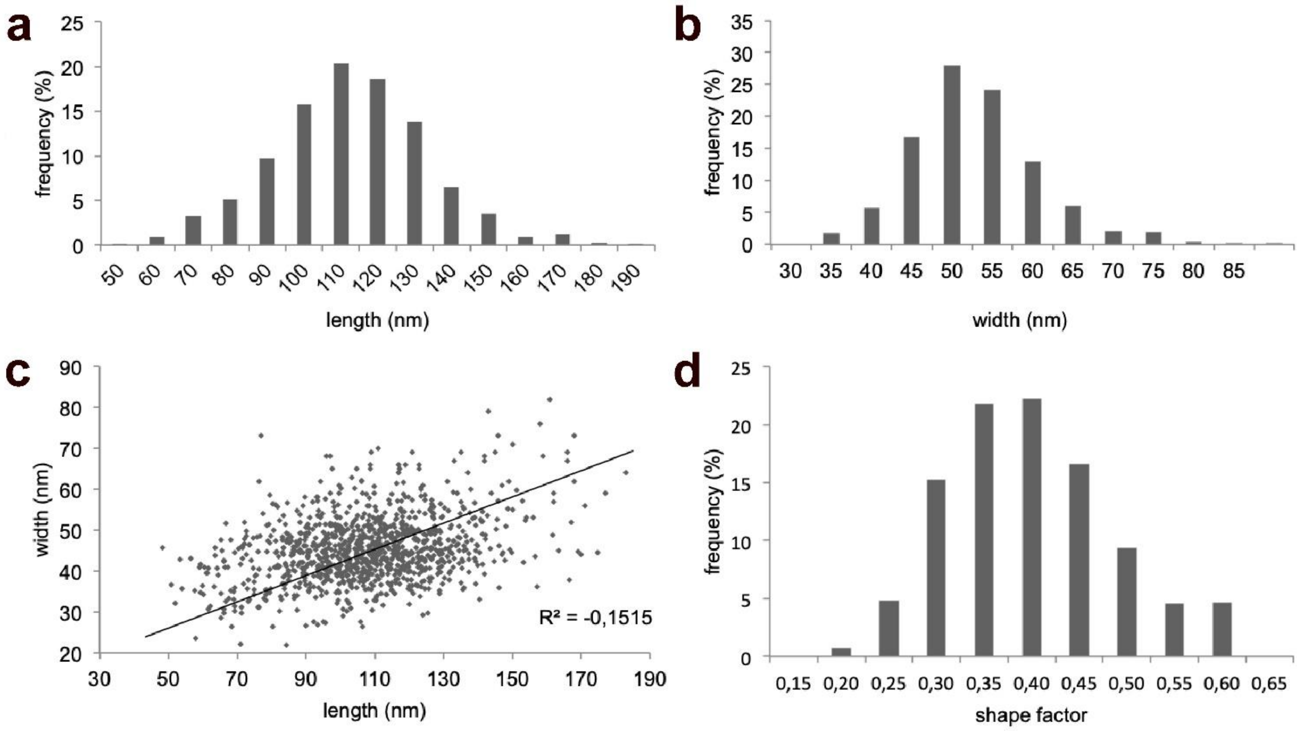

Supplementary figure S1. Crystal size metrics for *Candidatus* Magnetomonas plexicatena LBB01. **a** Length distribution; **b** width distribution; **c** length against width (aspect ratio); **d** shape factor distribution (n = 1061)

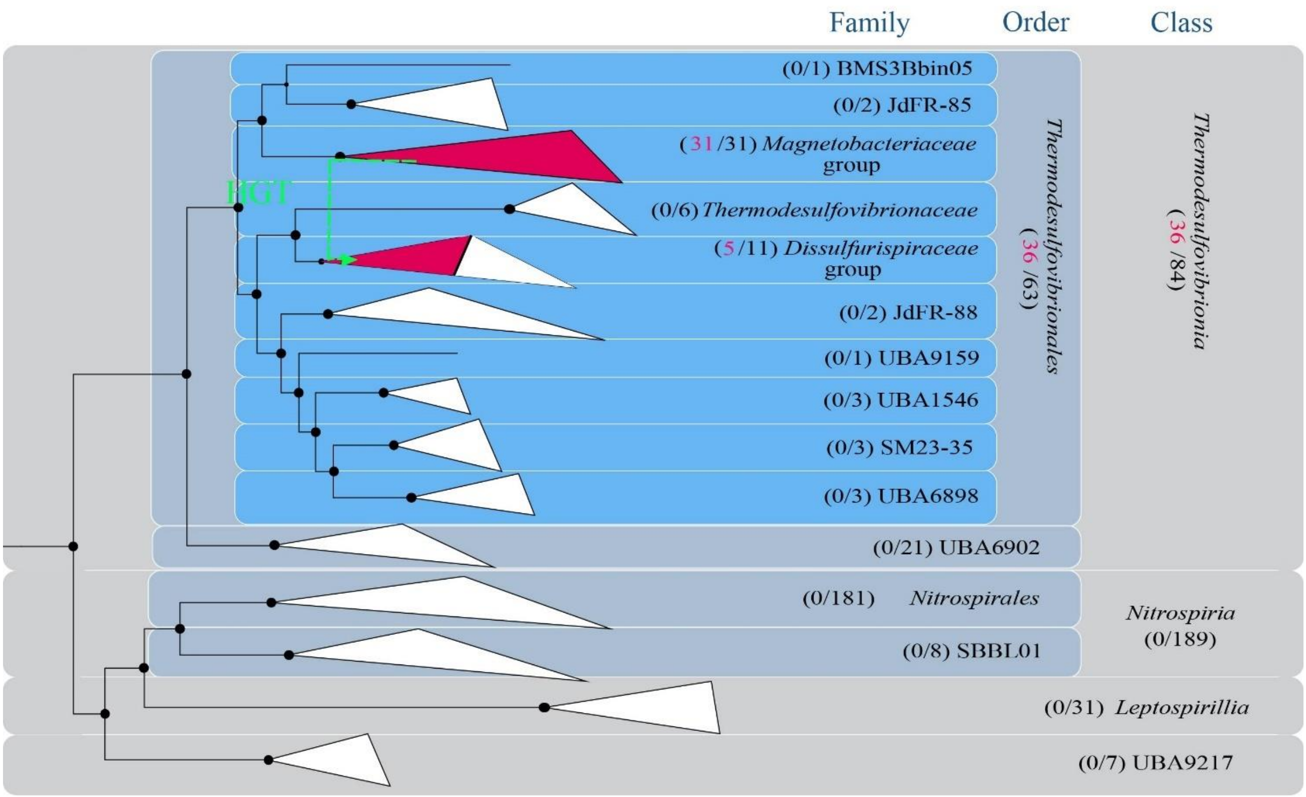

Supplementary figure S2. Phylogenomic analysis and reconciliation results for genomes from the *Nitrospirota* phylum including 36 MTB genomes. A maximum likelihood phylogenomic tree was inferred from concatenated 120 bacterial single-copy marker proteins, which was constructed with evolutionary model LG + F + I + G4. Branch supports were obtained with 1000 ultrafast bootstraps. The scale bar represents amino acid substitutions per site. Red-colored branches indicate groups that include MTB representatives. White-colored branches do not have MTB representatives. Green lines indicate the direction of horizontal gene transfer (HGT) of MGC

Supplementary table S1. Statistics for all bacterial genomes used in this work

Genome name	NCBI/IMG accession	Size, Mbp	Scaff, no.	GC, %	GTDB Taxonomy	Completeness, %	Contamination, %
Alphaproteobacteria bacterium nDJH14bin10	GCA_015233585.1	3.33	435	60.2	d_Bacteria;p_Proteobacteria;c_Alphaproteobacteria;o_Rhodospirillales;f_WOUV01;g_;s_	96.63	0.00
Alphaproteobacteria bacterium nJC1bin3	GCA_015232485.1	3.96	698	67.27	d_Bacteria;p_Proteobacteria;c_Alphaproteobacteria;o_Rhodospirillales;f_Magnetospirillaceae;g_;s_	86.90	2.94
Alphaproteobacteria bacterium nJC2bin54	GCA_015232435.1	3.89	694	67.6	d_Bacteria;p_Proteobacteria;c_Alphaproteobacteria;o_Rhodospirillales;f_Magnetospirillaceae;g_;s_	97.48	2.10
Alphaproteobacteria bacterium nKLKbin1	GCA_015232335.1	3.43	89	59.34	d_Bacteria;p_Proteobacteria;c_Alphaproteobacteria;o_Rhodospirillales;f_WMHbin7;g_WMHbin7;s_	99.50	0.00
Alphaproteobacteria bacterium nN2-2bin1	GCA_015232095.1	4.33	129	65.17	d_Bacteria;p_Proteobacteria;c_Alphaproteobacteria;o_Rhodospirillales;f_Magnetospirillaceae;g_Magnetospirillum;s_Magnetospirillum moscoviense	99.50	0.50
Alphaproteobacteria b`acterium nN2-2bin2	GCA_015232055.1	4.10	602	67.51	d_Bacteria;p_Proteobacteria;c_Alphaproteobacteria;o_Rhodospirillales;f_Magnetospirillaceae;g_;s_	96.97	1.82
Alphaproteobacteria bacterium nN3bin14	GCA_015232025.1	3.74	499	67.66	p_Proteobacteria;c_Alphaproteobacteria;o_Rhodospirillales;f_;g_;s_	61.19	2.10
Alphaproteobacteria bacterium nSSYDbin4	GCA_015231605.1	3.18	213	63.34	p_Proteobacteria;c_Alphaproteobacteria;o_Rhodospirillales;f_Magnetovibrionaceae;g_;s_	78.78	1.58
Alphaproteobacteria bacterium nTSbin12	GCA_015231505.1	2.39	329	63.33	p_Proteobacteria;c_Alphaproteobacteria;o_Rhodospirillales;f_Magnetovibrionaceae;g_;s_	78.43	0.00
Alphaproteobacteria bacterium nWMHbin5	GCA_015229215.1	3.56	80	59.35	p_Proteobacteria;c_Alphaproteobacteria;o_Rhodospirillales;f_WMHbin7;g_WMHbin7;s_WMHbin7 sp002753155	99.84	7.58
Alphaproteobacteria bacterium nWRX3bin4	GCA_015228915.1	2.59	411	66.09	p_Proteobacteria;c_Alphaproteobacteria;o_Rhodospirillales;f_WOUV01;g_;s_	97.66	0.00
Alphaproteobacteria bacterium nYQH56bin3	GCA_015228695.1	2.80	436	65.57	p_Proteobacteria;c_Alphaproteobacteria;o_Rhodospirillales;f_WOUV01;g_;s_	97.17	0.86
Alphaproteobacteria bacterium WMHbin7	GCA_002753155.1	2.98	73	59.84	p_Proteobacteria;c_Alphaproteobacteria;o_Rhodospirillales;f_WMHbin7;g_WMHbin7;s_WMHbin7 sp002753155	96.43	3.87
Bilophila wadsworthia ATCC 49260	GCF_000701705.1	4.63	198	59.25	p_Desulfobacterota;c_Desulfovibrionia;o_Desulfovibrionales;f_Desulfovibrionaceae;g_Bilophila;s_Bilophila wadsworthia	93.15	3.04
Caenispirillum salinarum AK4	GCF_000315795.1	4.95	61	68.79	p_Proteobacteria;c_Alphaproteobacteria;o_Rhodospirillales;f_Rhodospirillaceae;g_Caenispirillum;s_Caenispirillum salinarum	55.90	0.00
Caldimicrobium thiodismutans TF1	GCF_001548275.1	1.81	1	38.3	p_Desulfobacterota;c_Thermodesulfobacteria;o_Thermodesulfobacteriales;f_Thermodesulfobacteriaceae;g_Caldimicrobium;s_Caldimicrobium thiodismutans	99.95	1.29
Caldimicrobium thiodismutans ZAV-15	GCA_002877875.1	1.30	103	36.9	p_Desulfobacterota;c_Thermodesulfobacteria;o_Thermodesulfobacteriales;f_Thermodesulfobacteriaceae;g_Caldimicrobium;s_Caldimicrobium thiodismutans_A	84.30	3.23
Ca. Adiutrix intracellularis Adiu1	GCA_001577715.1	2.08	155	43.26	p_Desulfobacterota;c_Desulfarculia_A;o_Adiutricales;f_Adiutricaceae;g_Adiutrix;s_Adiutrix intracellularis	73.45	2.14
Ca. Desulfofervidus auxilii HS1	GCF_001577525.1	2.54	1	37.17	p_Desulfobacterota;c_Desulfofervidia;o_Desulfofervidales;f_Desulfofervidaceae;g_Desulfofervidus;s_Desulfofervidus auxilii	83.53	3.23
Ca. Electrothrix aarhusiensis MCF	GCA_004028505.1	3.73	143	47.47	p_Desulfobacterota;c_Desulfobulbia;o_Desulfobulbales;f_Desulfobulbaceae;g_Electrothrix;s_Electrothrix aarhusiensis	58.77	1.75
Ca. Electrothrix marina A5	GCA_004028495.1	2.06	472	49.7	p_Desulfobacterota;c_Desulfobulbia;o_Desulfobulbales;f_Desulfobulbaceae;g_Electrothrix;s_Electrothrix marina	91.29	3.71
Ca. Endomicrobium trichonymphae Rs-D17	GCA_002355835.1	1.13	4	35.23	p_Elusimicrobiota;c_Endomicrobia;o_Endomicrobiales;f_Endomicrobiaceae;g_Endomicrobium_A;s_Endomicrobium_A trichonymphae	90.32	0.65
Ca. Hydrogenedens terephthalicus JGI OTU-1	GCA_000493945.1	2.87	106	36.9	p_Hydrogenedentota;c_Hydrogenedentia;o_Hydrogenedentiales;f_Hydrogenedentaceae;g_Hydrogenedens;s_Hydrogenedens terephthalicus	49.08	0.97
Ca. Hydrogenedentes bacterium MAG_17963_hgd_111	GCA_013349485.1	3.02	288	60.18	p_Hydrogenedentota;c_Hydrogenedentia;o_Hydrogenedentiales;f_DUZN01;g_DUZN01;s_DUZN01 sp013349485	67.25	2.56
Ca. Hydrogenedentes bacterium MAG_17971_hgd_130	GCA_013349535.1	2.68	240	60.43	p_Hydrogenedentota;c_Hydrogenedentia;o_Hydrogenedentiales;f_DUZN01;g_DUZN01;s_DUZN01 sp013349535	90.20	3.64
Ca. Hydrogenedentes bacterium NORP43	GCA_002746185.1	4.23	202	49.96	p_Hydrogenedentota;c_Hydrogenedentia;o_Hydrogenedentiales;f_GCA-2746185;g_GCA-2746185;s_GCA-2746185 sp002746185	89.44	1.24
Ca. Hydrogenedentes bacterium UBA6118	GCA_002422525.1	4.51	94	67.12	p_Hydrogenedentota;c_Hydrogenedentia;o_Hydrogenedentiales;f_SLHB01;g_UBA6118;s_UBA6118 sp002422525	89.59	2.53
Ca. Lambdaproteobacteria bacterium PCRbin3	GCA_002753255.1	5.06	530	41.83	p_SAR324;c_SAR324;o_SAR324;f_GCA-2753255;g_GCA-2753255;s_GCA-2753255 sp002753255	97.67	2.73
Ca. Lambdaproteobacteria bacterium RIFOXYC1_FULL_56_13	GCA_001783655.1	3.09	66	56.53	p_SAR324;c_SAR324;o_XYD2-FULL-50-16;f_XYD2-FULL-50-16;g_XYC1-FULL-56-13;s_XYC1-FULL-56-13 sp001783715	100.00	0.97
Ca. Lambdaproteobacteria bacterium RIFOXYD2_FULL_50_16	GCA_001783695.1	3.14	54	49.65	p_SAR324;c_SAR324;o_XYD2-FULL-50-16;f_XYD2-FULL-50-16;g_XYD2-FULL-50-16;s_XYD2-FULL-50-16 sp001783695	98.71	2.26
Ca. Latescibacter anaerobius SCGC AAA252-E07	2264867254	2.29	164	42.12	p_Latescibacterota;c_Latescibacteria;o_Latescibacterales;f_Latescibacteraceae;g_Latescibacter;s_Latescibacter anaerobius	57.63	1.10
Ca. Latescibacteria bacterium 4484_107	GCA_002059205.1	1.60	192	55.50	p_Latescibacterota;c_Latescibacteria_A;o_4484-107;f_4484-107;g_4484-107;s_4484-107 sp002059205	76.68	0.00
Ca. Latescibacteria bacterium 4484_181	GCA_002049985.1	1.00	102	49.86	p_Latescibacterota;c_MVCY01;o_MVCY01;f_MVCY01;g_MVCY01;s_MVCY01 sp002049985	76.91	0.25
Ca. Latescibacteria bacterium 4484_7	GCA_002085285.1	1.53	161	50.94	p_Krumholzibacteriota;c_Krumholzibacteria;o_Krumholzibacteriales;f_Krumholzibacteriaceae;g_4484-7;s_4484-7 sp002085285	69.78	2.20
Ca. Magnetaquicoccus inordinatus UR-1	GCA_004217665.1	4.14	546	52.51	p_Proteobacteria;c_Magnetococcia;o_Magnetococcales;f_Magnetaquicoccaceae;g_Magnetaquicoccus;s_Magnetaquicoccus inordinatus	99.50	1.00
Ca. Magnetobacterium casensis MYR-1	GCF_000714715.1	3.42	70	48.87	p_Nitrospirota;c_Thermodesulfovibrionia;o_Thermodesulfovibrionales;f_Magnetobacteriaceae;g_Magnetobacterium;s_Magnetobacterium casensis	95.76	1.36
Ca. Magnetobacterium sp. XYC	GCA_018606805.1	3.59	91	37.75	p_Nitrospirota;c_Thermodesulfovibrionia;o_Thermodesulfovibrionales;f_Magnetobacteriaceae;g_;s_	92.94	0.42
Ca. Magnetobacterium sp. XYR	GCA_018606785.1	4.23	195	48.61	p_Nitrospirota;c_Thermodesulfovibrionia;o_Thermodesulfovibrionales;f_Magnetobacteriaceae;g_Magnetobacterium;s_Magnetobacterium sp002753685	73.26	1.37
Ca. Magnetomorum sp nER2bin1	GCA_015232875.1	6.16	393	38.94	p_Desulfobacterota;c_Desulfobacteria;o_Desulfobacterales;f_Magnetomoraceae;g_Magnetomorum;s_Magnetomorum sp002753725	90.97	3.55
Ca. Magnetomorum sp. HK-1	GCA_001292585.1	14.15	3036	34.61	p_Desulfobacterota;c_Desulfobacteria;o_Desulfobacterales;f_Magnetomoraceae;g_Magnetomorum;s_	90.31	4.20
Ca. Nitrospira inopinata ENR4	GCF_001458695.1	3.30	1	59.23	p_Nitrospirota;c_Nitrospiria;o_Nitrospirales;f_Nitrospiraceae;g_Nitrospira_F;s_Nitrospira_F inopinata	78.32	0.18
Ca. Nitrospira nitrificans COMA2	GCF_001458775.1	4.12	36	56.59	p_Nitrospirota;c_Nitrospiria;o_Nitrospirales;f_Nitrospiraceae;g_Nitrospira_F;s_Nitrospira_F nitrificans	97.48	2.94
Ca. Omnitrophica bacterium Cal1bin1	GCA_002753745.1	2.54	240	49.55	p_Omnitrophota;c_Koll11;o_UBA10015;f_GCA-002753745;g_GCA-2753745;s_GCA-2753745 sp002753745	76.15	3.23
Ca. Omnitrophica bacterium MBPbin6	GCA_002753465.1	2.18	150	49.49	p_Omnitrophota;c_Koll11;o_UBA10015;f_GCA-002753745;g_GCA-2753465;s_GCA-2753465 sp002753465	94.84	0.83
Ca. Omnitrophica bacterium nCal1bin2	GCA_015233765.1	2.95	196	49.66	p_Omnitrophota;c_Koll11;o_UBA10015;f_GCA-002753745;g_GCA-2753745;s_GCA-2753745 sp002753745	66.90	1.72
Ca. Omnitrophica bacterium nCal2bin1	GCA_015234065.1	1.75	176	51.48	p_Omnitrophota;c_Koll11;o_UBA10015;f_GCA-002753745;g_;s_	82.80	3.23
Ca. Omnitrophica bacterium nDJH13bin13	GCA_015233705.1	1.17	97	54.15	p_Omnitrophota;c_Koll11;o_UBA10015;f_GCA-002753745;g_;s_	95.77	0.00
Ca. Omnitrophica bacterium nDJH13bin20	GCA_015233675.1	2.12	31	42.8	p_Omnitrophota;c_Koll11;o_UBA10015;f_GCA-002753745;g_JABDGZ01;s_	69.64	0.00
Ca. Omnitrophica bacterium nDJH15bin13	GCA_015233495.1	2.10	111	49.08	p_Omnitrophota;c_Koll11;o_UBA10015;f_Kpj58rc;g_UBA12451;s_	97.32	0.00
Ca. Omnitrophica bacterium nDJH15bin6	GCA_015233405.1	2.38	47	43.25	p_Omnitrophota;c_Koll11;o_UBA10015;f_Kpj58rc;g_;s_	66.54	0.97
Ca. Omnitrophica bacterium nDJH2bin13	GCA_015233375.1	1.76	209	42.08	p_Omnitrophota;c_Koll11;o_UBA10015;f_GCA-002753745;g_;s_	81.25	0.65
Ca. Omnitrophica bacterium nDJH2bin18	GCA_015233335.1	1.85	38	44.09	p_Omnitrophota;c_Koll11;o_UBA10015;f_GCA-002753745;g_JABDGZ01;s_	95.17	1.97
Ca. Omnitrophica bacterium nDJH6bin13	GCA_015233235.1	1.59	175	47.17	p_Omnitrophota;c_Koll11;o_UBA1560;f_SKK-01;g_;s_	95.95	0.91
Ca. Omnitrophica bacterium nDJH6bin14	GCA_015233195.1	1.98	50	37.49	p_Omnitrophota;c_Koll11;o_UBA10015;f_Kpj58rc;g_UBA12451;s_	70.95	0.00
Ca. Omnitrophica bacterium nDJH6bin18	GCA_015233165.1	2.29	187	41.64	p_Omnitrophota;c_Koll11;o_UBA10015;f_GCA-002753745;g_;s_	71.27	1.82
Ca. Omnitrophica bacterium nDJH6bin28	GCA_015233145.1	1.81	58	47.32	p_Omnitrophota;c_Koll11;o_UBA10015;f_GCA-002753745;g_;s_	98.99	3.99

Genome name	NCBI/IMG accession	Size, Mbp	Scaff, no.	GC, %	GTDB Taxonomy	Completeness, %	Contamination, %
Ca. Omnitrophica bacterium nDJH6bin5	GCA_015233115.1	2.39	37	40.68	p_Omnitrophota;c_Koll11;o_UBA10015;f_Kpj58rc;g_UBA12451;s_	64.82	1.82
Ca. Omnitrophica bacterium nHGRbin18	GCA_015232565.1	2.64	80	51.11	p_Omnitrophota;c_Koll11;o_UBA10015;f_GCA-002753745;g_GCA-2753745;s_	77.53	1.69
Ca. Omnitrophica bacterium nHLHbin2	GCA_015232535.1	2.21	210	42.78	p_Omnitrophota;c_Koll11;o_UBA10015;f_Kpj58rc;g_;s_	67.24	0.65
Ca. Omnitrophica bacterium nMBPbin3	GCA_015232235.1	1.97	41	50.38	p_Omnitrophota;c_Koll11;o_UBA10015;f_GCA-002753745;g_GCA-2753465;s_GCA-2753465 sp002753465	65.52	0.00
Ca. Omnitrophica bacterium nS315bin20	GCA_015231685.1	1.38	200	56.96	p_Omnitrophota;c_Koll11;o_2-02-FULL-51-18;f_;g_;s_	98.03	0.91
Ca. Omnitrophica bacterium nS315bin24	GCA_015231715.1	1.50	115	37.11	p_Omnitrophota;c_Koll11;o_UBA1560;f_SKK-01;g_;s_	92.02	0.91
Ca. Omnitrophica bacterium nW3bin14	GCA_015231405.1	3.66	93	49.49	p_Omnitrophota;c_Koll11;o_UBA1560;f_SKK-01;g_;s_	79.69	2.15
Ca. Omnitrophica bacterium nXXbin4	GCA_015228785.1	2.02	123	46.42	p_Omnitrophota;c_Koll11;o_UBA10015;f_GCA-002753745;g_JAAYZZ01;s_	73.12	0.10
Ca. Omnitrophus fodinae SCGC AAA011-A17	GCA_000405945.1	2.04	150	49.56	p_Omnitrophota;c_Omnitrophia;o_Omnitrophales;f_Omnitrophaceae;g_Omnitrophus;s_Omnitrophus fodinae	83.33	1.08
Ca. Riflebacteria bacterium nDJH14bin3	GCA_015233565.1	8.30	185	40.61	p_Riflebacteria;c_Ozemobacteria;o_Ozemobacterales;f_Ozemobacteraceae;g_;s_	98.70	2.37
Ca. Riflebacteria bacterium nDJH6bin19	GCA_015233155.1	6.31	185	49.32	p_Riflebacteria;c_Ozemobacteria;o_Ozemobacterales;f_Ozemobacteraceae;g_;s_	99.91	0.60
Ca. Riflebacteria bacterium nHGRbin4	GCA_015232575.1	7.05	54	40.28	p_Riflebacteria;c_Ozemobacteria;o_Ozemobacterales;f_Ozemobacteraceae;g_;s_	98.06	0.00
Ca. Terasakiella magnetica PR-1	GCF_900093605.1	3.68	48	45.97	p_Proteobacteria;c_Alphaproteobacteria;o_Rhodospirillales;f_Terasakiellaceae;g_Terasakiella;s_Terasakiella magnetica_A	89.26	3.64
delta proteobacterium MLS_D	GCA_002030015.1	2.51	70	55.2	p_Desulfobacterota;c_Syntrophia;o_Syntrophales;f_UBA2210;g_MLS-D;s_MLS-D sp002030015	96.76	3.18
Delta proteobacterium NaphS2	GCA_000179315.1	6.55	810	49.83	p_Desulfobacterota;c_Desulfobacteria;o_Desulfatiglandales;f_Desulfatiglandaceae;g_NaphS2;s_NaphS2 sp000179315	97.85	0.00
Deltaproteobacteria bacterium 37-65-8	GCA_002279275.1	1.83	193	65.33	p_Desulfobacterota_E;c_MBNT15;o_MBNT15;f_MBNT15;g_CG2-30-66-27;s_CG2-30-66-27 sp002279275	70.32	0.65
Deltaproteobacteria bacterium B111_G9	GCA_003646995.1	3.85	542	47.56	p_Desulfobacterota;c_Desulfobacteria;o_Desulfatiglandales;f_Desulfatiglandaceae;g_B111-G9;s_B111-G9 sp003646995	61.93	0.33
Deltaproteobacteria bacterium B119_G9	GCA_003646715.1	2.74	559	43.47	p_Desulfobacterota;c_Syntrophobacteria;o_B119-G9;f_B119-G9;g_B119-G9;s_B119-G9 sp003646715	99.50	0.50
Deltaproteobacteria bacterium B13_G4	GCA_003647525.1	1.69	215	39.2	p_Desulfobacterota;c_Desulfobacteria;o_Desulfobacterales;f_ETH-SRB1;g_B13-G4;s_B13-G4 sp003647525	64.43	2.58
Deltaproteobacteria bacterium B17_G16	GCA_003646975.1	3.25	554	58.82	p_Desulfobacterota;c_Desulfobacteria;o_Desulfatiglandales;f_Desulfatiglandaceae;g_B17-G16;s_B17-G16 sp003646975	91.59	0.00
Deltaproteobacteria bacterium B25_G16	GCA_003646935.1	2.38	429	50.52	p_Desulfobacterota;c_Desulfobacteria;o_Desulfatiglandales;f_B25-G16;g_B25-G16;s_B25-G16 sp003646935	99.87	0.89
Deltaproteobacteria bacterium B3_G2	GCA_003647385.1	3.07	611	41.15	p_Desulfobacterota;c_Desulfobacteria;o_Desulfobacterales;f_Desulfobacteraceae;g_Desulfobacula;s_Desulfobacula sp003647385	91.29	0.75
Deltaproteobacteria bacterium B30_G6	GCA_003647375.1	5.41	703	47.76	p_Desulfobacterota;c_Desulfobacteria;o_Desulfobacterales;f_B30-G6;g_B30-G6;s_B30-G6 sp003647375	72.33	2.58
Deltaproteobacteria bacterium B33_G16	GCA_003646875.1	2.20	384	41.86	p_Desulfobacterota;c_Desulfobacteria;o_Desulfatiglandales;f_B25-G16;g_B33-G16;s_B33-G16 sp003646875	96.94	3.57
Deltaproteobacteria bacterium B37_G16	GCA_003646865.1	2.47	349	48.37	p_Desulfobacterota;c_Desulfobacteria;o_Desulfatiglandales;f_Desulfatiglandaceae;g_B111-G9;s_B111-G9 sp003646865	85.66	6.77
Deltaproteobacteria bacterium B46_G9	GCA_003646815.1	3.47	410	51.5	p_Desulfobacterota;c_Desulfobacteria;o_Desulfatiglandales;f_Desulfatiglandaceae;g_B46-G9;s_B46-G9 sp003646815	78.99	0.84
Deltaproteobacteria bacterium CG_4_8_14_3_um_filter_43_13	GCA_002782605.1	2.57	311	43.25	p_Desulfobacterota;c_SM23-61;o_UBA8473;f_UBA8473;g_UBA8473;s_UBA8473 sp002782605	88.06	0.70
Deltaproteobacteria bacterium CG03_land_8_20_14_0_80_45_14	GCA_002780715.1	3.24	253	45.5	p_Desulfobacterota;c_BSN033;o_BSN033;f_UBA1163;g_UBA1163;s_UBA1163 sp002780715	94.52	2.39
Deltaproteobacteria bacterium CG07_land_8_20_14_0_80_60_11	GCA_002779455.1	2.42	285	59.82	p_Desulfobacterota;c_Desulfobaccia;o_Desulfobaccales;f_0-14-0-80-60-11;g_0-14-0-80-60-11;s_0-14-0-80-60-11 sp002779455	100.00	0.00
Deltaproteobacteria bacterium CSP1-8	GCA_001443455.1	2.18	46	63.49	p_Desulfobacterota_E;c_MBNT15;o_MBNT15;f_MBNT15;g_CSP1-8;s_CSP1-8 sp001443455	95.45	2.00
Deltaproteobacteria bacterium D1FN1.001	GCA_005774545.1	3.10	115	65.26	p_Desulfobacterota_F;c_Desulfuromonadia;o_Desulfuromonadales;f_BM103;g_VAUL01;s_VAUL01 sp005774545	73.02	0.74
Deltaproteobacteria bacterium DOLJORAL78_54_23	GCA_002747175.1	1.85	61	53.53	p_Desulfobacterota;c_Desulfobacteria;o_Desulfobacterales;f_Desulfosarcinaceae;g_Desulfosarcina;s_Desulfosarcina sp002747175	54.03	0.00
Deltaproteobacteria bacterium DOLZORAL124_49_6	GCA_002747805.1	1.55	89	48.77	p_Desulfobacterota;c_Desulfobulbia;o_Desulfobulbales;f_Desulfocapsaceae;g_GCA-2747805;s_GCA-2747805 sp002747805	89.16	4.96
Deltaproteobacteria bacterium ER2bin7	GCA_002753725.1	5.97	653	38.47	p_Desulfobacterota;c_Desulfobacteria;o_Desulfobacterales;f_Magnetomoraceae;g_Magnetomorum;s_Magnetomorum sp002753725	86.90	1.29
Deltaproteobacteria bacterium GWA2_55_10	GCA_001797415.1	2.24	161	55.63	p_Desulfobacterota_F;c_GWC2-55-46;o_GWC2-55-46;f_GWC2-55-46;g_GWC2-55-46_A;s_GWC2-55-46_A sp001797415	96.09	0.65
Deltaproteobacteria bacterium GWC2_55_46	GCA_001595385.3	2.79	3	55.4	p_Desulfobacterota_F;c_GWC2-55-46;o_GWC2-55-46;f_GWC2-55-46;g_GWC2-55-46;s_GWC2-55-46 sp001595385	68.08	1.45
Deltaproteobacteria bacterium GWF2_42_12	GCA_001797665.1	2.44	126	42.05	p_Desulfobacterota_F;c_GWC2-55-46;o_GWC2-55-46;f_UBA9637;g_UBA9637;s_UBA9637 sp001797665	96.77	1.29
Deltaproteobacteria bacterium HGW-Deltaproteobacteria-1	GCA_002840635.1	3.30	558	47.91	p_Desulfobacterota;c_Syntrophia;o_Syntrophales;f_Smithellaceae;g_UBA8904;s_UBA8904 sp002840635	92.22	2.78
Deltaproteobacteria bacterium HGW-Deltaproteobacteria-12	GCA_002840565.1	3.99	64	47.28	p_Desulfobacterota;c_Syntrophia;o_Syntrophales;f_Smithellaceae;g_Fen-1166;s_Fen-1166 sp002840565	90.39	0.65
Deltaproteobacteria bacterium HGW-Deltaproteobacteria-13	GCA_002840555.1	3.51	29	45.65	p_Desulfobacterota;c_Syntrophia;o_Syntrophales;f_Smithellaceae;g_Smithella;s_Smithella sp002840555	98.35	1.29
Deltaproteobacteria bacterium HGW-Deltaproteobacteria-15	GCA_002840535.1	6.98	106	54.47	p_Desulfobacterota;c_Desulfobacteria;o_Desulfatiglandales;f_HGW-15;g_HGW-15;s_HGW-15 sp002840535	85.04	5.94
Deltaproteobacteria bacterium HGW-Deltaproteobacteria-19	GCA_002841865.1	3.98	87	59.86	p_Desulfobacterota;c_Syntrophia;o_Syntrophales;f_PHBD01;g_PHBD01;s_PHBD01 sp002841865	75.18	0.65
Deltaproteobacteria bacterium HGW-Deltaproteobacteria-2	GCA_002840505.1	3.28	11	42.84	p_Desulfobacterota;c_Syntrophia;o_Syntrophales;f_Smithellaceae;g_Smithella;s_Smithella sp002840505	83.50	3.87
Deltaproteobacteria bacterium HGW-Deltaproteobacteria-3	GCA_002841785.1	2.85	388	57.95	p_Desulfobacterota;c_Desulfobulbia;o_Desulfobulbales;f_Desulfurivibrionaceae;g_UBA2262;s_UBA2262 sp002841785	86.27	2.10
Deltaproteobacteria bacterium HGW-Deltaproteobacteria-4	GCA_002841765.1	3.02	30	55.77	p_Desulfobacterota_F;c_Desulfuromonadia;o_Desulfuromonadales;f_Trichloromonadaceae;g_UBA2197;s_UBA2197 sp002841765	96.76	3.92
Deltaproteobacteria bacterium HGW-Deltaproteobacteria-6	GCA_002840435.1	4.09	53	49.46	p_Desulfobacterota;c_Syntrophia;o_Syntrophales;f_Smithellaceae;g_UBA8904;s_UBA8904 sp002840435	96.02	0.65
Deltaproteobacteria bacterium M0040	GCA_006226895.1	1.63	270	53	p_Desulfobacterota_F;c_Desulfuromonadia;o_Desulfuromonadales;f_BM103;g_M0040;s_M0040 sp006226895	98.71	3.23
Deltaproteobacteria bacterium MAG 48	GCA_003973265.1	3.27	113	52.35	p_Desulfobacterota;c_Desulfobacteria;o_Desulfobacterales;f_QNYZ01;g_QNYZ01;s_QNYZ01 sp003973265	87.24	3.49
Deltaproteobacteria bacterium MAG_00134_naph_006	GCA_013349875.1	1.50	692	49.54	p_Desulfobacterota;c_Desulfobacteria;o_Desulfatiglandales;f_Desulfatiglandaceae;g_DUZB01;s_DUZB01 sp013349725	94.84	1.94
Deltaproteobacteria bacterium MAG_00241_naph_010	GCA_013349745.1	1.55	324	49.45	p_Desulfobacterota;c_Desulfobacteria;o_Desulfatiglandales;f_Desulfatiglandaceae;g_DUZB01;s_DUZB01 sp013349725	86.38	1.29
Deltaproteobacteria bacterium MAG_00792_naph_016	GCA_013349725.1	3.03	409	49.74	p_Desulfobacterota;c_Desulfobacteria;o_Desulfatiglandales;f_Desulfatiglandaceae;g_DUZB01;s_DUZB01 sp013349725	91.00	4.32
Deltaproteobacteria bacterium MAG_09788_naph_37	GCA_013349635.1	0.90	137	47.23	p_Desulfobacterota;c_Desulfobacteria;o_Desulfatiglandales;f_Desulfatiglandaceae;g_JACNIU01;s_	91.46	4.78
Deltaproteobacteria bacterium MAG_15370_dsfb_81	GCA_013349495.1	3.87	334	48.4	p_Desulfobacterota;c_Desulfobacteria;o_Desulfobacterales;f_SURF-15;g_DUZK01;s_DUZK01 sp013349495	74.22	3.23
Deltaproteobacteria bacterium MAG_17929_sntb_26	GCA_013349515.1	2.78	276	53.1	p_Desulfobacterota;c_Syntrophobacteria;o_Syntrophobacterales;f_Syntrophobacteraceae;g_SbD1;s_	90.45	5.43
Deltaproteobacteria bacterium MAG_17996_sntb_20	GCA_013349395.1	1.69	454	53.11	p_Desulfobacterota;c_Syntrophobacteria;o_Syntrophobacterales;f_Syntrophobacteraceae;g_SbD1;s_	68.18	2.76
Deltaproteobacteria bacterium MAG_22204_dsfv_001	GCA_013349285.1	2.68	75	52.74	p_Desulfobacterota;c_Desulfobulbia;o_Desulfobulbales;f_Desulfurivibrionaceae;g_DUZU01;s_DUZU01 sp013349285	81.84	0.00
Deltaproteobacteria bacterium MAG_22309_dsfv_022	GCA_013349295.1	2.90	66	55.15	p_Desulfobacterota;c_Desulfobulbia;o_Desulfobulbales;f_Desulfurivibrionaceae;g_UBA2262;s_UBA2262 sp013349295	95.00	3.86
Deltaproteobacteria bacterium nDJH13bin5	GCA_015233625.1	4.59	619	51.85	p_Desulfobacterota;c_Desulfobaccia_A;o_;f_;g_;s_	90.97	5.16
Deltaproteobacteria bacterium nDJH2bin9	GCA_015233355.1	4.76	633	51.72	p_Desulfobacterota;c_Desulfobaccia_A;o_;f_;g_;s_	99.58	0.84

Genome name	NCBI/IMG accession	Size, Mbp	Scaff, no.	GC, %	GTDB Taxonomy	Completeness, %	Contamination, %
Deltaproteobacteria bacterium nDJH5bin8	GCA_015233285.1	3.75	521	51.53	p_Desulfobacterota;c_Desulfobacteria;o_;f_;g_;s_	98.39	2.58
Deltaproteobacteria bacterium nDJH6bin12	GCA_015233295.1	3.95	569	51.41	p_Desulfobacterota;c_Desulfobaccia_A;o_;f_;g_;s_	99.35	1.61
Deltaproteobacteria bacterium nDJH6bin20	GCA_015233135.1	2.61	64	45.14	p_UBA10199;c_UBA10199;o_DSSB01;f_;g_;s_	95.85	3.18
Deltaproteobacteria bacterium nDJH8bin5	GCA_015233055.1	4.05	527	51.56	p_Desulfobacterota;c_Desulfobaccia_A;o_;f_;g_;s_	93.19	0.39
Deltaproteobacteria bacterium nXXbin1	GCA_015228875.1	4.58	228	47.04	p_UBA10199;c_UBA10199;o_;f_;g_;s_	99.70	0.89
Deltaproteobacteria bacterium Phox-21	GCA_001896555.1	3.31	63	57.7	p_Desulfobacterota;c_Desulfomonilia;o_UBA1062;f_UBA1062;g_UBA1062;s_UBA1062 sp001896555	83.26	2.47
Deltaproteobacteria bacterium PowLak16_MAG11	GCA_007280345.1	2.43	223	50.5	p_Desulfobacterota;c_QYQD01;o_QYQD01;f_QYQD01;g_QYQD01;s_QYQD01 sp007280345	77.26	1.68
Deltaproteobacteria bacterium PowLak16_MAG18	GCA_007280195.1	1.67	140	65.04	p_Desulfobacterota_E;c_MBNT15;o_MBNT15;f_MBNT15;g_CG2-30-66-27;s_CG2-30-66-27 sp007280195	81.61	1.45
Deltaproteobacteria bacterium RBG_13_43_22	GCA_001797675.1	3.23	39	48.06	p_Desulfobacterota;c_Desulfobaccia_A;o_RBG-13-43-22;f_RBG-13-43-22;g_RBG-13-43-22;s_RBG-13-43-22 sp001797675	58.23	1.35
Deltaproteobacteria bacterium RBG_13_49_15	GCA_001797695.1	2.59	201	49.16	p_Desulfobacterota;c_Desulfobacteria;o_Desulfobacterales;f_UBA2156;g_RBG-13-49-15;s_RBG-13-49-15 sp001797695	94.44	0.85
Deltaproteobacteria bacterium RBG_13_52_11	GCA_001797745.1	2.54	107	52.05	p_Desulfobacterota;c_BSN033;o_B13-G15;f_RBG-16-54-18;g_RBG-13-52-11;s_RBG-13-52-11 sp001797745	100.00	0.00
Deltaproteobacteria bacterium RBG_16_44_11	GCA_001797845.1	2.43	47	43.54	p_Desulfobacterota;c_Syntrophia;o_Syntrophales;f_Smithellaceae;g_UBA4810;s_UBA4810 sp001797845	97.74	1.64
Deltaproteobacteria bacterium RBG_16_54_11	GCA_001797905.1	2.37	139	53.8	p_Desulfobacterota;c_BSN033;o_B13-G15;f_RBG-16-54-18;g_RBG-16-54-11;s_RBG-16-54-11 sp001797905	92.95	6.42
Deltaproteobacteria bacterium RIFCSPHIGHO2_02_FULL_43_33	GCA_001798165.1	2.19	126	42.54	p_Desulfobacterota_F;c_GWC2-55-46;o_GWC2-55-46;f_UBA9637;g_UBA10170;s_UBA10170 sp001798165	91.10	3.38
Deltaproteobacteria bacterium RIFOXYD12_FULL_50_9	GCA_001799225.1	3.77	274	50.16	p_Desulfobacterota;c_Desulfobulbia;o_Desulfobulbales;f_Desulfurivibrionaceae;g_XYD12-FULL-50-9;s_XYD12-FULL-50-9 sp001799225	76.34	1.13
Deltaproteobacteria bacterium RIFOXYD12_FULL_55_16	GCA_001799255.1	2.46	90	55.67	p_Desulfobacterota;c_Desulfobulbia;o_Desulfobulbales;f_Desulfurivibrionaceae;g_UBA2262;s_UBA2262 sp001799255	95.45	0.60
Deltaproteobacteria bacterium RIFOXYD12_FULL_56_24	GCA_001799285.1	3.04	67	56.42	p_Desulfobacterota;c_Desulfobulbia;o_Desulfobulbales;f_Desulfurivibrionaceae;g_UBA2262;s_UBA2262 sp001799285	74.86	1.66
Deltaproteobacteria bacterium RIFOXYD12_FULL_57_12	GCA_001799275.1	3.84	126	56.76	p_Desulfobacterota;c_Desulfobulbia;o_Desulfobulbales;f_BM004;g_YD12-FULL-57-12;s_YD12-FULL-57-12 sp001799275	86.90	2.10
Deltaproteobacteria bacterium SM23_61	GCA_001304105.1	4.24	286	56.18	p_Desulfobacterota;c_SM23-61;o_SM23-61;f_SM23-61;g_SM23-61;s_SM23-61 sp001304105	98.32	1.68
Deltaproteobacteria bacterium SURF_34	GCA_003599365.1	1.92	108	43.36	p_Desulfobacterota;c_SM23-61;o_UBA8473;f_UBA8473;g_UBA8473;s_UBA8473 sp003599365	94.12	2.10
Deltaproteobacteria bacterium SURF_52	GCA_003599195.1	3.92	179	57.38	p_Desulfobacterota;c_Desulfobaccia;o_Desulfobaccales;f_0-14-0-80-60-11;g_SURF-52;s_SURF-52 sp003599195	98.88	0.00
Deltaproteobacteria bacterium tcs-42	GCA_002049545.1	2.55	121	48.34	p_Desulfobacterota_F;c_Desulfuromonadia;o_Desulfuromonadales;f_Geopsychrobacteraceae;g_Desulfuromusa;s_Desulfuromusa sp002049545	91.94	7.31
Deltaproteobacteria bacterium UBA11853	GCA_003513855.1	2.01	259	64.22	p_Desulfobacterota_E;c_MBNT15;o_MBNT15;f_MBNT15;g_CSP1-8;s_CSP1-8 sp003513855	97.42	5.16
Deltaproteobacteria bacterium UBA1386	GCA_002305765.1	3.57	121	57.67	p_Desulfobacterota_G;c_Syntrophorhabdia;o_Syntrophorhabdales;f_Syntrophorhabdaceae;g_Delta-02;s_Delta-02 sp002305765	76.60	1.68
Deltaproteobacteria bacterium YD0425bin50	GCA_002753105.1	4.97	589	36.91	p_Desulfobacterota;c_Desulfobacteria;o_Desulfobacterales;f_YD0425bin50;g_YD0425bin50;s_YD0425bin50 sp002753105	96.94	1.45
Deltaproteobacteria bacterium YD0425bin51	GCA_002753225.1	5.22	134	32.23	p_Desulfobacterota;c_Desulfobacteria;o_Desulfobacterales;f_YD0425bin51;g_YD0425bin51;s_YD0425bin51 sp002753225	96.77	1.02
Desulfacinum hydrothermale DSM 13146	GCF_900176285.1	3.70	59	60.97	p_Desulfobacterota;c_Syntrophobacteria;o_Syntrophobacterales;f_DSM-9756;g_Desulfacinum;s_Desulfacinum hydrothermale	89.58	1.69
Desulfacinum infernum DSM 9756	GCF_900129305.1	4.24	81	61.97	p_Desulfobacterota;c_Syntrophobacteria;o_Syntrophobacterales;f_DSM-9756;g_Desulfacinum;s_Desulfacinum infernum	70.52	0.67
Desulfamplus magnetovallimortis PRJEB14757	GCF_900170035.1	6.68	108	40.72	p_Desulfobacterota;c_Desulfobacteria;o_Desulfobacterales;f_Desulfobacteraceae;g_Desulfamplus;s_Desulfamplus magnetovallimortis	92.20	4.77
Desulfamplus sp nDH2bin3	GCA_015233755.1	2.54	407	35.65	p_Desulfobacterota;c_Desulfobacteria;o_Desulfobacterales;f_Desulfobacteraceae;g_Desulfamplus;s_	95.45	0.91
Desulfamplus sp nER1bin2	GCA_015232935.1	2.88	486	42.66	p_Desulfobacterota;c_Desulfobacteria;o_Desulfobacterales;f_Desulfobacteraceae;g_Desulfamplus;s_	93.18	1.42
Desulfamplus sp nHGRbin17	GCA_015232555.1	3.82	106	37.82	p_Desulfobacterota;c_Desulfobacteria;o_Desulfobacterales;f_Desulfobacteraceae;g_Desulfamplus;s_	83.99	1.08
Desulfamplus sp nHLHbin7	GCA_015232465.1	3.02	147	37.75	p_Desulfobacterota;c_Desulfobacteria;o_Desulfobacterales;f_Desulfobacteraceae;g_Desulfamplus;s_	80.42	0.00
Desulfamplus sp nJC1bin9	GCA_015232455.1	4.35	82	35.99	p_Desulfobacterota;c_Desulfobacteria;o_Desulfobacterales;f_Desulfobacteraceae;g_Desulfamplus;s_	67.05	0.91
Desulfamplus sp nN2-2bin5	GCA_015232035.1	3.18	215	37.93	p_Desulfobacterota;c_Desulfobacteria;o_Desulfobacterales;f_Desulfobacteraceae;g_Desulfamplus;s_	96.36	0.00
Desulfamplus sp nS315bin3	GCA_015231705.1	3.60	282	40.67	p_Desulfobacterota;c_Desulfobacteria;o_Desulfobacterales;f_Desulfobacteraceae;g_Desulfamplus;s_	96.77	0.32
Desulfamplus sp nS315bin44	GCA_015231615.1	3.86	200	36.17	p_Desulfobacterota;c_Desulfobacteria;o_Desulfobacterales;f_Desulfobacteraceae;g_Desulfamplus;s_	83.90	1.69
Desulfamplus sp nS315bin9	GCA_015231655.1	3.17	195	37.59	p_Desulfobacterota;c_Desulfobacteria;o_Desulfobacterales;f_Desulfobacteraceae;g_Desulfamplus;s_	94.12	0.81
Desulfamplus sp nTSbin15	GCA_015231525.1	5.49	851	40.46	p_Desulfobacterota;c_Desulfobacteria;o_Desulfobacterales;f_Desulfobacteraceae;g_Desulfamplus;s_	91.91	0.89
Desulfamplus sp nTSbin20	GCA_015231465.1	3.63	402	47.21	p_Desulfobacterota;c_Desulfobacteria;o_Desulfobacterales;f_Desulfobacteraceae;g_Desulfamplus;s_	94.28	0.00
Desulfamplus sp. nTSbin4	GCA_015231415.1	3.23	196	37.6	p_Desulfobacterota;c_Desulfobacteria;o_Desulfobacterales;f_Desulfobacteraceae;g_Desulfamplus;s_	93.21	1.19
Desulfamplus sp. nXXbin12	GCA_015228835.1	2.59	369	37.76	p_Desulfobacterota;c_Desulfobacteria;o_Desulfobacterales;f_Desulfobacteraceae;g_Desulfamplus;s_	84.99	1.59
Desulfamplus_sp nTSbin21	GCA_015231395.1	2.81	548	39.13	p_Desulfobacterota;c_Desulfobacteria;o_Desulfobacterales;f_Desulfobacteraceae;g_Desulfamplus;s_	90.33	0.60
Desulfarculaceae bacterium JdFR-95	GCA_002011835.1	2.81	499	68.22	p_Desulfobacterota;c_Desulfarculia;o_Desulfarculales;f_Desulfarculaceae;g_JdFR-95;s_JdFR-95 sp002011835	95.45	0.00
Desulfarculales bacterium UBA696	GCA_002298995.1	4.17	77	53.8	p_Desulfobacterota;c_Syntrophobacteria;o_Syntrophobacterales;f_Syntrophobacteraceae;g_UBA696;s_UBA696 sp002298995	82.83	1.79
Desulfarculales bacterium UBA702	GCA_002298165.1	4.63	119	52.03	p_Desulfobacterota;c_Syntrophobacteria;o_Syntrophobacterales;f_Syntrophobacteraceae;g_UBA696;s_UBA696 sp002298165	61.13	1.44
Desulfarculus baarsii DSM 2075	GCF_000143965.1	3.66	1	65.7	p_Desulfobacterota;c_Desulfarculia;o_Desulfarculales;f_Desulfarculaceae;g_Desulfarculus;s_Desulfarculus baarsii	100.00	1.77
Desulfarculus sp. SURF_10	GCA_003605055.1	3.60	78	66.98	p_Desulfobacterota;c_Desulfarculia;o_Desulfarculales;f_Desulfarculaceae;g_SURF-10;s_SURF-10 sp003605055	100.00	0.00
Desulfatibacillum aliphaticivorans DSM 15576	GCF_000429905.1	6.47	64	54.43	p_Desulfobacterota;c_Desulfobacteria;o_Desulfobacterales;f_Desulfatibacillaceae;g_Desulfatibacillum;s_Desulfatibacillum aliphaticivorans	76.75	0.65
Desulfatibacillum alkenivorans DSM 16219	GCF_900142135.1	6.47	102	54.95	p_Desulfobacterota;c_Desulfobacteria;o_Desulfobacterales;f_Desulfatibacillaceae;g_Desulfatibacillum;s_Desulfatibacillum alkenivorans	77.77	1.45
Desulfatiglans anilini DSM 4660	GCF_000422285.1	4.67	107	58.78	p_Desulfobacterota;c_Desulfobacteria;o_Desulfatiglandales;f_Desulfatiglandaceae;g_Desulfatiglans;s_Desulfatiglans anilini	98.18	0.91
Desulfatirhabdium butyrativorans DSM 18734	GCF_000429925.1	4.48	73	54.92	p_Desulfobacterota;c_Desulfobacteria;o_Desulfobacterales;f_Desulfatirhabdiaceae;g_Desulfatirhabdium;s_Desulfatirhabdium butyrativorans	80.00	5.34
Desulfatitalea sp. BRH_c12	GCA_000961655.1	6.00	91	55.05	p_Desulfobacterota;c_Desulfobacteria;o_Desulfobacterales;f_Desulfosarcinaceae;g_Desulfatitalea;s_Desulfatitalea sp000961655	98.18	0.83
Desulfatitalea tepidiphila S28bF	GCF_001293685.1	5.61	7	56.68	p_Desulfobacterota;c_Desulfobacteria;o_Desulfobacterales;f_Desulfosarcinaceae;g_Desulfatitalea;s_Desulfatitalea tepidiphila	88.71	3.23
Desulfobacca acetoxidans DSM 11109	GCF_000195295.1	3.28	1	52.89	p_Desulfobacterota;c_Desulfobaccia;o_Desulfobaccales;f_Desulfobaccaceae;g_Desulfobacca;s_Desulfobacca acetoxidans	66.01	1.08
Desulfobacca sp. 4484_104	GCA_002049795.1	2.65	181	53.64	p_Desulfobacterota;c_Desulfobaccia;o_Desulfobaccales;f_Desulfobaccaceae;g_Desulfobacca_B;s_Desulfobacca_B sp002049795	88.32	2.25
Desulfobacter curvatus DSM 3379	GCF_000373985.1	5.64	234	46.9	p_Desulfobacterota;c_Desulfobacteria;o_Desulfobacterales;f_Desulfobacteraceae;g_Desulfobacter;s_Desulfobacter curvatus	93.58	2.78
Desulfobacter hydrogenophilus AcRS1	GCF_004319545.1	5.16	3	46.53	p_Desulfobacterota;c_Desulfobacteria;o_Desulfobacterales;f_Desulfobacteraceae;g_Desulfobacter;s_Desulfobacter hydrogenophilus	56.31	0.00

Genome name	NCBI/IMG accession	Size, Mbp	Scaff, no.	GC, %	GTDB Taxonomy	Completeness, %	Contamination, %
Desulfobacter postgatei 2ac9	GCF_000233695.2	3.97	1	47.2	p_Desulfobacterota;c_Desulfobacteria;o_Desulfobacterales;f_Desulfobacteraceae;g_Desulfobacter;s_Desulfobacter postgatei	54.15	0.65
Desulfobacter postgatei DOLJORAL78_47_202	GCA_002747145.1	1.48	65	47	p_Desulfobacterota;c_Desulfobacteria;o_Desulfobacterales;f_Desulfobacteraceae;g_Desulfobacter;s_Desulfobacter postgatei_A	71.94	0.09
Desulfobacter vibrioformis DSM 8776	GCF_000745975.1	4.47	86	48.64	p_Desulfobacterota;c_Desulfobacteria;o_Desulfobacterales;f_Desulfobacteraceae;g_Desulfobacter;s_Desulfobacter vibrioformis	89.77	0.00
Desulfobacteraceae bacterium 4484_190.2	GCA_002050025.1	2.37	232	45.82	p_Desulfobacterota;c_Desulfobacteria;o_Desulfatiglandales;f_Desulfatiglandaceae;g_4484-190-2;s_4484-190-2 sp002050025	62.78	3.98
Desulfobacteraceae bacterium 4572_123	GCA_002084545.1	3.18	168	47.51	p_Desulfobacterota;c_Desulfobacteria;o_Desulfobacterales;f_4572-123;g_4572-123;s_4572-123 sp002084545	90.99	1.68
Desulfobacteraceae bacterium 4572_130	GCA_002084425.1	1.94	24	29.47	p_Desulfobacterota;c_Desulfobacteria;o_Desulfobacterales;f_Desulfobacteraceae;g_4572-130;s_4572-130 sp002084425	88.04	0.65
Desulfobacteraceae bacterium 4572_35.2	GCA_002084665.1	1.90	129	46.14	p_Desulfobacterota_F;c_Desulfuromonadia;o_Desulfuromonadales;f_Desulfuromonadaceae;g_Desulfuromonas;s_Desulfuromonas sp002084665	61.51	1.66
Desulfobacteraceae bacterium 4572_89	GCA_002085465.1	1.70	98	43.64	p_Desulfobacterota;c_Desulfobacteria;o_Desulfobacterales;f_Desulfobacteraceae;g_NBML01;s_NBML01 sp002085465	98.49	1.94
Desulfobacteraceae bacterium B1Sed10_16	GCA_003551985.1	2.12	442	54.15	p_Desulfobacterota;c_Desulfobacteria;o_Desulfobacterales;f_SURF-3;g_B1SED10-16;s_B1SED10-16 sp003551985	95.85	3.69
Desulfobacteraceae bacterium BM002	GCA_002899795.1	3.80	638	50.24	p_Desulfobacterota;c_Syntrophobacteria;o_BM002;f_BM002;g_BM002;s_BM002 sp002899795	93.64	6.39
Desulfobacteraceae bacterium BM005	GCA_002868985.1	3.05	577	43.45	p_Desulfobacterota;c_Desulfobacteria;o_Desulfobacterales;f_UBA11574;g_UBA11574;s_UBA11574 sp002868985	54.79	0.00
Desulfobacteraceae bacterium CG2_30_51_40	GCA_001874005.1	3.44	229	50.72	p_Desulfobacterota;c_Desulfobacteria;o_Desulfatiglandales;f_Desulfatiglandaceae;g_CG2-30-51-40;s_CG2-30-51-40 sp001874005	97.10	0.65
Desulfobacteraceae bacterium CSSed165cm_505	GCA_007131845.1	2.05	410	55.22	p_Desulfobacterota;c_Desulfobacteria;o_Desulfobacterales;f_SURF-3;g_B1SED10-16;s_B1SED10-16 sp007131845	95.85	3.69
Desulfobacteraceae bacterium Eth-SRB2	GCA_004193595.1	5.26	83	43.72	p_Desulfobacterota;c_Desulfobacteria;o_Desulfobacterales;f_UBA11574;g_S5133MH16;s_S5133MH16 sp004193595	81.21	0.32
Desulfobacteraceae bacterium maxbin2.1429	GCA_003819975.1	5.14	978	48.48	p_Desulfobacterota;c_Desulfobacteria;o_Desulfatiglandales;f_HGW-15;g_RPPU01;s_RPPU01 sp003819975	92.80	1.68
Desulfobacteraceae bacterium RAAP-1	GCA_001443525.1	4.09	193	49.94	p_Desulfobacterota;c_Desulfobacteria;o_Desulfobacterales;f_Desulfatirhabdiaceae;g_RAAP-1;s_RAAP-1 sp001443525	93.87	1.33
Desulfobacteraceae bacterium SURF_15	GCA_003599475.1	5.29	268	54.29	p_Desulfobacterota;c_Desulfobacteria;o_Desulfobacterales;f_SURF-15;g_SURF-15;s_SURF-15 sp003599475	95.48	1.61
Desulfobacteraceae bacterium SURF_3	GCA_003599885.1	4.51	90	49.04	p_Desulfobacterota;c_Desulfobacteria;o_Desulfobacterales;f_SURF-3;g_SURF-3;s_SURF-3 sp003599885	92.06	4.60
Desulfobacteraceae bacterium SURF_33	GCA_003597945.1	3.72	137	52.78	p_Desulfobacterota;c_Desulfobacteria;o_Desulfobacterales;f_SURF-3;g_SURF-33;s_SURF-33 sp003597945	80.65	0.00
Desulfobacteraceae bacterium SURF_4	GCA_003599575.1	6.45	197	55.75	p_Desulfobacterota;c_Desulfobacteria;o_Desulfobacterales;f_Desulfosarcinaceae;g_Desulfatitalea;s_Desulfatitalea sp003599575	68.49	0.86
Desulfobacteraceae bacterium SURF_67	GCA_003599015.1	3.00	149	49.3	p_Desulfobacterota;c_Desulfobacteria;o_Desulfatiglandales;f_Desulfatiglandaceae;g_CG2-30-51-40;s_CG2-30-51-40 sp003599015	98.81	0.62
Desulfobacteraceae bacterium UBA11574	GCA_003486165.1	2.82	505	43.35	p_Desulfobacterota;c_Desulfobacteria;o_Desulfobacterales;f_UBA11574;g_UBA11574;s_UBA11574 sp003486165	98.74	0.84
Desulfobacteraceae bacterium UBA2156	GCA_002328165.1	2.94	78	45.87	p_Desulfobacterota;c_Desulfobacteria;o_Desulfobacterales;f_UBA2156;g_UBA2156;s_UBA2156 sp002328165	95.80	3.64
Desulfobacteraceae bacterium UBA2771	GCA_002352605.1	3.03	196	54.14	p_Desulfobacterota;c_Desulfobacteria;o_Desulfobacterales;f_Desulfosarcinaceae;g_Desulfosarcina;s_Desulfosarcina sp002352605	93.88	7.28
Desulfobacteraceae bacterium UBA4064	GCA_002382065.1	4.77	300	50.19	p_Desulfobacterota;c_Desulfobacteria;o_Desulfobacterales;f_Desulfatirhabdiaceae;g_UBA4064;s_UBA4064 sp002382065	82.58	4.52
Desulfobacteraceae bacterium UBA5616	GCA_002423615.1	3.66	121	51.61	p_Desulfobacterota;c_Desulfobacteria;o_Desulfobacterales;f_UBA5616;g_UBA5616;s_UBA5616 sp002423615	65.27	0.00
Desulfobacteraceae bacterium UBA5623	GCA_002424495.1	4.02	456	47.46	p_Desulfobacterota;c_Desulfobacteria;o_Desulfatiglandales;f_Desulfatiglandaceae;g_UBA5623;s_UBA5623 sp002424495	96.77	1.29
Desulfobacteraceae bacterium UBA8212	GCA_003538835.1	5.56	584	53.41	p_Desulfobacterota;c_Desulfobacteria;o_Desulfobacterales;f_Desulfobacteraceae;g_Desulfobacter;s_Desulfobacter sp003538835	88.71	0.20
Desulfobacterales bacterium CG2_30_60_27	GCA_001873115.1	2.52	157	60.25	p_Desulfobacterota;c_Desulfobulbia;o_Desulfobulbales;f_Desulfurivibrionaceae;g_CG2-30-60-27;s_CG2-30-60-27 sp001873115	97.42	0.32
Desulfobacterales bacterium CG23	GCA_002771315.1	2.75	309	52.32	p_Desulfobacterota;c_Desulfobacteria;o_Desulfobacterales;f_UBA2156;g_GCA-002779465;s_GCA-002779465 sp002771315	94.09	2.73
Desulfobacterales bacterium CSSed165cm_255	GCA_007134875.1	2.88	231	55.67	p_Desulfobacterota;c_Desulfobacteria;o_Desulfobacterales;f_SURF-3;g_B1SED10-16;s_B1SED10-16 sp007134875	91.82	5.73
Desulfobacterales bacterium GWB2_56_26	GCA_001799365.1	4.51	263	55.56	p_Desulfobacterota;c_Desulfobulbia;o_Desulfobulbales;f_Desulfocapsaceae;g_Desulforhopalus;s_Desulforhopalus sp001799365	91.40	3.41
Desulfobacterales bacterium nHLHbin5	GCA_015232515.1	4.58	421	32.52	p_Desulfobacterota;c_Desulfobacteria;o_Desulfobacterales;f_YD0425bin51;g_YD0425bin51;s_YD0425bin51 sp002753225	92.58	3.37
Desulfobacterales bacterium nTSbin1	GCA_015231595.1	5.13	210	32.05	p_Desulfobacterota;c_Desulfobacteria;o_Desulfobacterales;f_YD0425bin50;g_;s_	81.38	2.66
Desulfobacterales bacterium nYD0425bin5	GCA_015228685.1	5.63	133	32.38	p_Desulfobacterota;c_Desulfobacteria;o_Desulfobacterales;f_YD0425bin51;g_YD0425bin51;s_YD0425bin51 sp002753225	94.49	0.71
Desulfobacterales bacterium nYD0425bin6	GCA_015228675.1	5.42	467	36.82	p_Desulfobacterota;c_Desulfobacteria;o_Desulfobacterales;f_YD0425bin50;g_YD0425bin50;s_YD0425bin50 sp002753105	90.90	0.65
Desulfobacterales bacterium S5133MH16	GCA_001751005.1	3.02	574	43.59	p_Desulfobacterota;c_Desulfobacteria;o_Desulfobacterales;f_UBA11574;g_S5133MH16;s_S5133MH16 sp001751005	80.59	4.52
Desulfobacterium autotrophicum HRM2	GCF_000020365.1	5.66	2	48.76	p_Desulfobacterota;c_Desulfobacteria;o_Desulfobacterales;f_Desulfobacteraceae;g_Desulfobacterium_B;s_Desulfobacterium_B autotrophicum	84.88	2.58
Desulfobacterium vacuolatum DSM 3385	GCF_900176365.1	5.04	80	46.54	p_Desulfobacterota;c_Desulfobacteria;o_Desulfobacterales;f_Desulfobacteraceae;g_Desulfobacterium_A;s_Desulfobacterium_A vacuolatum	91.07	0.60
Desulfobacula toluolica Tol2	GCF_000307105.1	5.20	1	41.45	p_Desulfobacterota;c_Desulfobacteria;o_Desulfobacterales;f_Desulfobacteraceae;g_Desulfobacula;s_Desulfobacula toluolica	95.81	0.00
Desulfobotulus mexicanus PAR22N	GCF_006175995.1	3.83	69	48.98	p_Desulfobacterota;c_Desulfobacteria;o_Desulfobacterales;f_Desulforegulaceae;g_Desulfobotulus;s_Desulfobotulus mexicanus	98.18	1.82
Desulfobulbaceae bacterium BM004	GCA_002868955.1	2.66	189	47.45	p_Desulfobacterota;c_Desulfobulbia;o_Desulfobulbales;f_BM004;g_BM004;s_BM004 sp002868955	88.17	2.90
Desulfobulbaceae bacterium BM506	GCA_002868945.1	3.71	83	56.29	p_Desulfobacterota;c_Desulfobulbia;o_Desulfobulbales;f_Desulfurivibrionaceae;g_BM506;s_BM506 sp002868945	92.58	2.58
Desulfobulbaceae bacterium BRH_c16a	GCA_000961725.1	6.52	72	51.68	p_Desulfobacterota;c_Desulfobulbia;o_Desulfobulbales;f_Desulfocapsaceae;g_Desulforhopalus;s_Desulforhopalus sp000961725	93.12	2.78
Desulfobulbaceae bacterium CSSed10_361	GCA_003557565.1	2.23	357	59.21	p_Desulfobacterota;c_Desulfobulbia;o_Desulfobulbales;f_Desulfurivibrionaceae;g_Desulfurivibrio;s_Desulfurivibrio sp003557565	57.04	0.12
Desulfobulbaceae bacterium DB1	GCA_001914235.1	3.87	28	53.78	p_Desulfobacterota;c_Desulfobulbia;o_Desulfobulbales;f_Desulfurivibrionaceae;g_DB1;s_DB1 sp001914235	90.88	0.59
Desulfobulbaceae bacterium Del10	GCA_004332195.1	1.66	21	54.26	p_Desulfobacterota;c_Desulfobulbia;o_Desulfobulbales;f_Desulfurivibrionaceae;g_Desulfurivibrio;s_Desulfurivibrio sp004332195	74.06	1.82
Desulfobulbaceae bacterium nTSbin18	GCA_015231515.1	3.41	225	45.02	p_Desulfobacterota;c_Desulfobulbia;o_Desulfobulbales;f_Desulfurivibrionaceae;g_;s_	84.17	1.94
Desulfobulbaceae bacterium SZUA-575	GCA_003249645.1	3.74	182	45.85	p_Desulfobacterota;c_Desulfobulbia;o_Desulfobulbales;f_Desulfocapsaceae;g_SZUA-575;s_SZUA-575 sp003249645	96.77	5.48
Desulfobulbaceae bacterium SZUA-615	GCA_003249165.1	3.54	497	45.36	p_Desulfobacterota;c_Desulfobulbia;o_Desulfobulbales;f_Desulfurivibrionaceae;g_SURF-16;s_SURF-16 sp003249165	84.70	5.65
Desulfobulbaceae bacterium UBA10518	GCA_003508005.1	3.01	95	57.91	p_Desulfobacterota;c_Desulfobulbia;o_Desulfobulbales;f_Desulfobulbaceae;g_UBA10518;s_UBA10518 sp003508005	76.96	2.58
Desulfobulbaceae bacterium UBA11700	GCA_003517965.1	2.52	81	53.21	p_Desulfobacterota;c_Desulfobulbia;o_Desulfobulbales;f_Desulfurivibrionaceae;g_UBA2262;s_UBA2262 sp003517965	90.30	3.64
Desulfobulbaceae bacterium UBA2213	GCA_002327225.1	2.46	214	51.85	p_Desulfobacterota;c_Desulfobulbia;o_Desulfobulbales;f_Desulfocapsaceae;g_UBA2270;s_UBA2270 sp002327225	91.35	6.85
Desulfobulbaceae bacterium UBA2262	GCA_002347185.1	2.85	141	61.68	p_Desulfobacterota;c_Desulfobulbia;o_Desulfobulbales;f_Desulfurivibrionaceae;g_UBA2262;s_UBA2262 sp002347185	80.42	1.29
Desulfobulbaceae bacterium UBA2270	GCA_002347745.1	3.95	171	48.8	p_Desulfobacterota;c_Desulfobulbia;o_Desulfobulbales;f_Desulfocapsaceae;g_UBA2270;s_UBA2270 sp002347745	89.19	0.65
Desulfobulbaceae bacterium UBA2273	GCA_002347095.1	3.20	33	57.57	p_Desulfobacterota;c_Desulfobulbia;o_Desulfobulbales;f_Desulfurivibrionaceae;g_UBA2262;s_UBA2262 sp002347095	77.42	3.23
Desulfobulbaceae bacterium UBA2276	GCA_002347085.1	1.79	68	51.37	p_Desulfobacterota;c_Desulfobulbia;o_Desulfobulbales;f_Desulfocapsaceae;g_UBA2270;s_UBA2270 sp002347085	94.84	3.46
Desulfobulbaceae bacterium UBA5121	GCA_002414225.1	3.53	59	63.19	p_Desulfobacterota;c_Desulfobulbia;o_Desulfobulbales;f_Desulfurivibrionaceae;g_UBA5123;s_UBA5123 sp002414225	53.42	0.00
Desulfobulbaceae bacterium UBA5123	GCA_002415465.1	2.28	240	61.01	p_Desulfobacterota;c_Desulfobulbia;o_Desulfobulbales;f_Desulfurivibrionaceae;g_UBA5123;s_UBA5123 sp002415465	86.93	2.07

Genome name	NCBI/IMG accession	Size, Mbp	Scaff, no.	GC, %	GTDB Taxonomy	Completeness, %	Contamination, %
Desulfobulbaceae bacterium UBA5611	GCA_002424635.1	2.95	95	50.29	p_Desulfobacterota;c_Desulfobulbia;o_Desulfobulbales;f_Desulfocapsaceae;g_UBA2270;s_UBA2270 sp002424635	96.63	0.00
Desulfobulbaceae bacterium UBA5628	GCA_002421565.1	1.80	197	60.1	p_Desulfobacterota;c_Desulfobulbia;o_Desulfobulbales;f_Desulfurivibrionaceae;g_UBA5628;s_UBA5628 sp002421565	98.90	1.10
Desulfobulbus mediterraneus DSM 13871	GCF_000429965.1	4.80	68	57.66	p_Desulfobacterota;c_Desulfobulbia;o_Desulfobulbales;f_Desulfobulbaceae;g_Desulfobulbus_A;s_Desulfobulbus_A mediterraneus	85.16	0.65
Desulfobulbus propionicus DOLZORAL124_48_34	GCA_002746685.1	1.61	53	47.78	p_Desulfobacterota;c_Desulfobulbia;o_Desulfobulbales;f_Desulfobulbaceae;g_Desulfobulbus_A;s_Desulfobulbus_A propionicus_C	85.58	0.07
Desulfobulbus sp. SURF_48	GCA_003604995.1	2.64	108	59.65	p_Desulfobacterota;c_Desulfobulbia;o_Desulfobulbales;f_Desulfobulbaceae;g_UBA10518;s_UBA10518 sp003604995	89.65	2.58
Desulfocarbo indianensis SCBM	GCF_001184205.1	5.11	334	63.05	p_Desulfobacterota;c_Desulfarculia;o_Desulfarculales;f_Desulfarculaceae;g_Desulfocarbo;s_Desulfocarbo indianensis	98.45	0.00
Desulfococcus multivorans DSM 2059	GCF_001854245.1	4.46	1	56.83	p_Desulfobacterota;c_Desulfobacteria;o_Desulfobacterales;f_Desulfococcaceae;g_Desulfococcus;s_Desulfococcus multivorans	98.02	0.15
Desulfoluna spongiiphila AA1	GCF_900101345.1	6.54	52	57.24	p_Desulfobacterota;c_Desulfobacteria;o_Desulfobacterales;f_Desulfolunaceae;g_Desulfoluna;s_Desulfoluna spongiiphila	94.55	1.82
Desulfonatronospira sp. CSSed162cmB_39	GCA_007127015.1	1.31	200	49.04	p_Desulfobacterota;c_Desulfovibrionia;o_Desulfovibrionales;f_Desulfonatronovibrionaceae;g_Desulfonatronospira;s_Desulfonatronospira sp007127015	70.18	0.88
Desulfonatronospira sp. CSSed162cmB_565	GCA_007125075.1	2.17	149	49.19	p_Desulfobacterota;c_Desulfovibrionia;o_Desulfovibrionales;f_Desulfonatronovibrionaceae;g_Desulfonatronospira;s_Desulfonatronospira sp007125075	86.57	0.00
Desulfonatronovibrio hydrogenovorans DSM 9292	GCF_000686525.1	2.94	16	50.35	p_Desulfobacterota;c_Desulfovibrionia;o_Desulfovibrionales;f_Desulfonatronovibrionaceae;g_Desulfonatronovibrio;s_Desulfonatronovibrio hydrogenovorans	76.63	1.54
Desulfonatronovibrio magnus AHT22	GCF_000934755.1	4.81	134	44.86	p_Desulfobacterota;c_Desulfovibrionia;o_Desulfovibrionales;f_Desulfonatronovibrionaceae;g_Desulfonatronovibrio;s_Desulfonatronovibrio magnus	95.81	8.63
Desulfonatronovibrio sp. CSSed10_448R1	GCA_003558575.1	1.76	360	43.61	p_Desulfobacterota;c_Desulfovibrionia;o_Desulfovibrionales;f_Desulfonatronovibrionaceae;g_Desulfonatronovibrio;s_Desulfonatronovibrio sp003558575	96.13	3.55
Desulfonauticus sp. 38_4375	GCA_001507915.1	2.13	107	38.21	p_Desulfobacterota;c_Desulfovibrionia;o_Desulfovibrionales;f_Desulfonauticaceae;g_Desulfonauticus;s_Desulfonauticus sp001507915	82.99	2.90
Desulfonauticus submarinus DSM 15269	GCF_900104045.1	2.10	23	32.47	p_Desulfobacterota;c_Desulfovibrionia;o_Desulfovibrionales;f_Desulfonauticaceae;g_Desulfonauticus;s_Desulfonauticus submarinus	95.48	2.71
Desulfonema ishimotonii Tokyo 01	GCF_003851005.1	6.64	9	53.5	p_Desulfobacterota;c_Desulfobacteria;o_Desulfobacterales;f_Desulfococcaceae;g_Desulfonema;s_Desulfonema ishimotonii	97.73	4.55
Desulfopila aestuarii DSM 18488	GCF_900143695.1	6.07	103	49.63	p_Desulfobacterota;c_Desulfobulbia;o_Desulfobulbales;f_Desulfocapsaceae;g_Desulfopila;s_Desulfopila aestuarii	93.23	1.29
Desulfopila sp. IMCC35005	GCA_005116655.1	6.69	63	44.28	p_Desulfobacterota;c_Desulfobulbia;o_Desulfobulbales;f_Desulfocapsaceae;g_Desulfopila;s_Desulfopila sp005116655	96.45	0.65
Desulfopila sp. IMCC35006	GCF_005116645.1	5.62	95	49.75	p_Desulfobacterota;c_Desulfobulbia;o_Desulfobulbales;f_Desulfocapsaceae;g_Desulforhopalus;s_Desulforhopalus sp005116645	99.34	0.20
Desulforegula conservatrix Mb1Pa	GCF_000426225.1	4.47	178	42.39	p_Desulfobacterota;c_Desulfobacteria;o_Desulfobacterales;f_Desulforegulaceae;g_Desulforegula;s_Desulforegula conservatrix	98.18	0.91
Desulforhabdus sp. SDB_sulfate2	GCA_007244385.1	5.20	119	54.39	p_Desulfobacterota;c_Syntrophobacteria;o_Syntrophobacterales;f_Syntrophobacteraceae;g_Desulforhabdus;s_Desulforhabdus sp007244385	78.46	1.04
Desulforhopalus sp. IMCC35007	GCF_005116575.1	5.74	127	45.99	p_Desulfobacterota;c_Desulfobulbia;o_Desulfobulbales;f_Desulfocapsaceae;g_Desulforhopalus;s_Desulforhopalus sp005116575	66.62	1.49
Desulfosarcina cetonica JCM 12296	GCF_001311845.1	7.09	558	55.73	p_Desulfobacterota;c_Desulfobacteria;o_Desulfobacterales;f_Desulfosarcinaceae;g_Desulfosarcina;s_Desulfosarcina cetonica	98.71	0.00
Desulfosediminicola ganghwensis IMCC35004	GCA_005116675.2	5.65	1	48.43	p_Desulfobacterota;c_Desulfobulbia;o_Desulfobulbales;f_Desulfocapsaceae;g_Desulfopila;s_Desulfopila sp005116675	95.45	2.73
Desulfotalea sp. NORP6	GCA_002733995.1	4.12	52	44.41	p_Desulfobacterota;c_Desulfobulbia;o_Desulfobulbales;f_Desulfocapsaceae;g_Desulforhopalus;s_Desulforhopalus sp002733995	92.12	0.00
Desulfotignum balticum DSM 7044	GCF_000421285.1	5.12	5	51.24	p_Desulfobacterota;c_Desulfobacteria;o_Desulfobacterales;f_Desulfobacteraceae;g_Desulfotignum;s_Desulfotignum balticum	99.58	0.84
Desulfovibrio alkalitolerans DSM 16529	GCF_000422245.1	3.20	32	64.48	p_Desulfobacterota;c_Desulfovibrionia;o_Desulfovibrionales;f_Desulfovibrionaceae;g_Desulfohalovibrio;s_Desulfohalovibrio alkalitolerans	100.00	4.98
Desulfovibrio aminophilus DSM 12254	GCF_000422565.1	3.42	25	66.25	p_Desulfobacterota;c_Desulfovibrionia;o_Desulfovibrionales;f_Desulfovibrionaceae;g_Aminidesulfovibrio;s_Aminidesulfovibrio aminophilus	84.52	1.33
Desulfovibrio brasiliensis JCM 12178	GCF_001311825.1	3.57	182	59.65	p_Desulfobacterota;c_Desulfovibrionia;o_Desulfovibrionales;f_Desulfovibrionaceae;g_Pseudodesulfovibrio;s_Pseudodesulfovibrio brasiliensis	98.18	0.91
Desulfovibrio carbinolicus DSM 3852	GCF_004135975.1	4.69	6	64.62	p_Desulfobacterota;c_Desulfovibrionia;o_Desulfovibrionales;f_Desulfovibrionaceae;g_Solidesulfovibrio;s_Solidesulfovibrio carbinolicus	68.40	2.15
Desulfovibrio carbinoliphilus subsp. oakridgensis FW-101-2B	GCA_000177215.2	4.22	3	66.44	p_Desulfobacterota;c_Desulfovibrionia;o_Desulfovibrionales;f_Desulfovibrionaceae;g_Solidesulfovibrio;s_Solidesulfovibrio carbinoliphilus	74.36	2.52
Desulfovibrio cuneatus DSM 11391	GCF_000430005.1	3.36	76	53.55	p_Desulfobacterota;c_Desulfovibrionia;o_Desulfovibrionales;f_Desulfovibrionaceae;g_Frigididesulfovibrio;s_Frigididesulfovibrio cuneatus	59.35	0.65
Desulfovibrio fairfieldensis CCUG 45958	GCF_001553605.1	3.70	1	60.9	p_Desulfobacterota;c_Desulfovibrionia;o_Desulfovibrionales;f_Desulfovibrionaceae;g_Desulfovibrio;s_Desulfovibrio fairfieldensis	70.44	2.64
Desulfovibrio gracilis DSM 16080	GCF_900167125.1	3.18	53	58.43	p_Desulfobacterota;c_Desulfovibrionia;o_Desulfovibrionales;f_Desulfovibrionaceae;g_Paucidesulfovibrio;s_Paucidesulfovibrio gracilis	96.49	0.91
Desulfovibrio hydrothermalis AM13 = DSM 14728	GCF_000331025.1	3.71	2	45.15	p_Desulfobacterota;c_Desulfovibrionia;o_Desulfovibrionales;f_Desulfovibrionaceae;g_Maridesulfovibrio;s_Maridesulfovibrio hydrothermalis	95.33	0.84
Desulfovibrio legallii H1T	GCF_004309735.1	2.67	35	62.92	p_Desulfobacterota;c_Desulfovibrionia;o_Desulfovibrionales;f_Desulfovibrionaceae;g_Desulfovibrio;s_Desulfovibrio legallii	66.85	1.71
Desulfovibrio legallii KHC7	GCF_900102485.1	2.70	36	64.81	p_Desulfobacterota;c_Desulfovibrionia;o_Desulfovibrionales;f_Desulfovibrionaceae;g_Desulfovibrio;s_Desulfovibrio legallii_A	67.17	3.42
Desulfovibrio longus DSM 6739	GCF_000420485.1	3.70	19	63.66	p_Desulfobacterota;c_Desulfovibrionia;o_Desulfovibrionales;f_Desulfovibrionaceae;g_Paucidesulfovibrio;s_Paucidesulfovibrio longus	94.24	3.18
Desulfovibrio magneticus str. Maddingley MBC34	GCA_000307955.1	4.39	489	65.71	p_Desulfobacterota;c_Desulfovibrionia;o_Desulfovibrionales;f_Desulfovibrionaceae;g_Solidesulfovibrio;s_Solidesulfovibrio magneticus_A	98.99	4.73
Desulfovibrio magneticus UBA7700	GCA_002482565.1	4.22	217	65.61	p_Desulfobacterota;c_Desulfovibrionia;o_Desulfovibrionales;f_Desulfovibrionaceae;g_Solidesulfovibrio;s_Solidesulfovibrio magneticus_B	96.57	3.36
Desulfovibrio mexicanus DSM 13116	GCF_900188225.1	3.54	15	65.68	p_Desulfobacterota;c_Desulfovibrionia;o_Desulfovibrionales;f_Desulfovibrionaceae;g_Humidesulfovibrio;s_Humidesulfovibrio mexicanus	91.54	0.12
Desulfovibrio oxyclinae DSM 11498	GCF_000375485.1	3.32	32	59.12	p_Desulfobacterota;c_Desulfovibrionia;o_Desulfovibrionales;f_Desulfovibrionaceae;g_Pseudodesulfovibrio;s_Pseudodesulfovibrio oxyclinae	74.00	2.73
Desulfovibrio piger ATCC 29098	GCF_000156375.1	2.87	47	63.05	p_Desulfobacterota;c_Desulfovibrionia;o_Desulfovibrionales;f_Desulfovibrionaceae;g_Desulfovibrio;s_Desulfovibrio piger	99.91	0.63
Desulfovibrio sp. DV	GCF_001936595.1	4.85	219	63.38	p_Desulfobacterota;c_Desulfovibrionia;o_Desulfovibrionales;f_Desulfovibrionaceae;g_Solidesulfovibrio;s_Solidesulfovibrio sp001936595	98.25	4.20
Desulfovibrio sp. TomC	GCF_000801335.2	5.07	84	61.71	p_Desulfobacterota;c_Desulfovibrionia;o_Desulfovibrionales;f_Desulfovibrionaceae;g_Solidesulfovibrio;s_Solidesulfovibrio sp000801335	99.11	1.68
Desulfovibrio sp. UBA4079	GCA_002382645.1	2.86	208	68.01	p_Desulfobacterota;c_Desulfovibrionia;o_Desulfovibrionales;f_Desulfovibrionaceae;g_Aminidesulfovibrio;s_Aminidesulfovibrio sp002382645	87.10	0.86
Desulfovibrio sp. UBA6121	GCA_002422505.1	3.18	178	65.01	p_Desulfobacterota;c_Desulfovibrionia;o_Desulfovibrionales;f_Desulfovibrionaceae;g_Humidesulfovibrio;s_Humidesulfovibrio sp002422505	93.53	2.10
Desulfovibrio sp. X2	GCF_000422205.1	3.91	66	67.99	p_Desulfobacterota;c_Desulfovibrionia;o_Desulfovibrionales;f_Desulfovibrionaceae;g_Desulfohalovibrio;s_Desulfohalovibrio sp000422205	62.87	1.00
Desulfovibrio zosterae DSM 11974	GCF_000425265.1	4.09	15	41.76	p_Desulfobacterota;c_Desulfovibrionia;o_Desulfovibrionales;f_Desulfovibrionaceae;g_Maridesulfovibrio;s_Maridesulfovibrio zosterae	85.35	2.73
Desulfovibrionaceae bacterium nDJH2bin10	GCA_015233395.1	3.37	158	62.81	p_Desulfobacterota;c_Desulfovibrionia;o_Desulfovibrionales;f_Desulfovibrionaceae;g_;s_	94.49	1.19
Desulfovibrionaceae bacterium nDJH8bin10	GCA_015233065.1	3.29	255	62.71	p_Desulfobacterota;c_Desulfovibrionia;o_Desulfovibrionales;f_Desulfovibrionaceae;g_;s_	90.74	2.16
Desulfovibrionaceae bacterium UBA930	GCA_002293605.1	2.10	253	58.27	p_Desulfobacterota;c_Desulfovibrionia;o_Desulfovibrionales;f_Desulfovibrionaceae;g_Frigididesulfovibrio;s_Frigididesulfovibrio sp002293605	70.44	0.13
Desulfurivibrio alkaliphilus AHT 2	GCF_000092205.1	3.10	1	60.29	p_Desulfobacterota;c_Desulfobulbia;o_Desulfobulbales;f_Desulfurivibrionaceae;g_Desulfurivibrio;s_Desulfurivibrio alkaliphilus	93.55	1.29
Desulfurivibrio sp. SURF_16	GCA_003605035.1	4.22	118	57.21	p_Desulfobacterota;c_Desulfobulbia;o_Desulfobulbales;f_Desulfurivibrionaceae;g_SURF-16;s_SURF-16 sp003605035	92.58	1.29
Desulfuromonadaceae bacterium CSSed165cm_319	GCA_007134025.1	2.25	149	58.5	p_Desulfobacterota_F;c_Desulfuromonadia;o_Desulfuromonadales;f_Syntrophotaleaceae;g_SLLR01;s_SLLR01 sp007134025	86.13	1.61
Desulfuromonadaceae bacterium GWB2_53_15	GCA_001824455.1	1.83	189	53.47	p_Desulfobacterota_F;c_Desulfuromonadia;o_Geobacterales;f_Pseudopelobacteraceae;g_Pseudopelobacter;s_Pseudopelobacter sp001824455	95.22	0.00
Desulfuromonadaceae bacterium SZUA-401	GCA_003246035.1	1.69	120	63.06	p_Desulfobacterota_F;c_Desulfuromonadia;o_Desulfuromonadales;f_SZUA-401;g_SZUA-401;s_SZUA-401 sp003246035	97.40	3.78
Desulfuromonadaceae bacterium UBA5613	GCA_002424625.1	2.38	144	56.32	p_Desulfobacterota_F;c_Desulfuromonadia;o_Desulfuromonadales;f_Trichloromonadaceae;g_UBA2197;s_UBA2197 sp002424625	93.35	3.64
Desulfuromonadaceae bacterium UBA6124	GCA_002423365.1	3.37	399	61.42	p_Desulfobacterota_F;c_Desulfuromonadia;o_Desulfuromonadales;f_Trichloromonadaceae;g_Trichloromonas;s_Trichloromonas sp002423365	96.76	3.64

Genome name	NCBI/IMG accession	Size, Mbp	Scaff, no.	GC, %	GTDB Taxonomy	Completeness, %	Contamination, %
Desulfuromonadales bacterium C00003068	GCA_001751155.1	2.74	461	46.28	p_Desulfobacterota_F;c_Desulfuromonadia;o_Desulfuromonadales;f_Desulfuromonadaceae;g_Desulfuromonas;s_Desulfuromonas sp001751155	92.26	0.32
Desulfuromonadales bacterium CSSed11_297R1	GCA_003561875.1	2.22	239	58.23	p_Desulfobacterota_F;c_Desulfuromonadia;o_Desulfuromonadales;f_Syntrophotaleaceae;g_SLLR01;s_SLLR01 sp003561875	90.06	7.28
Desulfuromonadales bacterium GT-UBC1	GCA_003712145.1	4.04	110	59.89	p_Desulfobacterota_F;c_Desulfuromonadia;o_Geobacterales;f_Geobacteraceae;g_Geobacter;s_Geobacter sp003712145	100.00	0.60
Desulfuromonadales bacterium MAG_13126_9_058	GCA_013349325.1	3.58	72	52.01	p_Desulfobacterota_F;c_Desulfuromonadia;o_Geobacterales;f_Pseudopelobacteraceae;g_Pseudopelobacter;s_Pseudopelobacter sp013349325	65.68	0.00
Desulfuromonadales bacterium MAG_21600_9_004	GCA_013349405.1	3.43	60	51.5	p_Desulfobacterota_F;c_Desulfuromonadia;o_Geobacterales;f_Pseudopelobacteraceae;g_Pseudopelobacter;s_Pseudopelobacter sp013349405	96.13	1.19
Desulfuromonadales bacterium MAG_21601_9_030	GCA_013349415.1	2.54	232	54.11	p_Desulfobacterota_F;c_Desulfuromonadia;o_Geobacterales;f_Pseudopelobacteraceae;g_Pseudopelobacter;s_	95.83	3.01
Desulfuromonas acetexigens DSM 1397	GCF_900111775.1	3.68	41	60.34	p_Desulfobacterota_F;c_Desulfuromonadia;o_Desulfuromonadales;f_Trichloromonadaceae;g_Trichloromonas;s_Trichloromonas acetexigens	93.58	4.80
Desulfuromonas acetoxidans DSM 684	GCF_000167355.1	3.83	51	51.83	p_Desulfobacterota_F;c_Desulfuromonadia;o_Desulfuromonadales;f_Desulfuromonadaceae;g_Desulfuromonas;s_Desulfuromonas acetoxidans	94.17	0.15
Desulfuromonas sp. BM302	GCA_002868925.1	2.57	216	56.03	p_Desulfobacterota_F;c_Desulfuromonadia;o_Desulfuromonadales;f_UBA2294;g_BM707;s_BM707 sp002868925	96.14	8.18
Desulfuromonas sp. BM508	GCA_002868865.1	2.66	109	55.18	p_Desulfobacterota_F;c_Desulfuromonadia;o_Desulfuromonadales;f_Geopsychrobacteraceae;g_BM509;s_BM509 sp002868865	79.80	1.35
Desulfuromonas sp. BM509	GCA_002869695.1	3.29	219	54.98	p_Desulfobacterota_F;c_Desulfuromonadia;o_Desulfuromonadales;f_Geopsychrobacteraceae;g_BM509;s_BM509 sp002869695	81.70	0.65
Desulfuromonas sp. BM513	GCA_002869685.1	3.12	186	52.53	p_Desulfobacterota_F;c_Desulfuromonadia;o_Desulfuromonadales;f_Desulfuromonadaceae;g_Desulfuromonas;s_Desulfuromonas sp002869685	95.71	7.19
Desulfuromonas sp. BM707	GCA_002869615.1	3.08	121	53.07	p_Desulfobacterota_F;c_Desulfuromonadia;o_Desulfuromonadales;f_UBA2294;g_BM707;s_BM707 sp002869615	98.18	2.73
Desulfuromonas sp. BM709	GCA_002869605.1	3.03	226	57.94	p_Desulfobacterota_F;c_Desulfuromonadia;o_Desulfuromonadales;f_SZUA-401;g_SZUA-401;s_SZUA-401 sp002869605	90.97	2.26
Desulfuromusa kysingii DSM 7343	GCF_900107645.1	3.74	27	46.66	p_Desulfobacterota_F;c_Desulfuromonadia;o_Desulfuromonadales;f_Geopsychrobacteraceae;g_Desulfuromusa;s_Desulfuromusa kysingii	88.39	6.33
Dethiosulfatarculus sandiegensis SPR	GCF_000931935.2	5.93	82	52.06	p_Desulfobacterota;c_Desulfarculia;o_Desulfarculales;f_Desulfarculaceae;g_Dethiosulfatarculus;s_Dethiosulfatarculus sandiegensis	97.67	2.27
Dissulfurispira thermophila T55J	GCA_014701235.1	2.37	1	38.74	p_Nitrospirota;c_Thermodesulfovibrionia;o_Thermodesulfovibrionales;f_UBA9935;g_UBA665;s_	63.54	0.93
Elusimicrobia bacterium GWA2_69_24	GCA_001799695.1	4.20	132	68.59	p_Elusimicrobiota;c_Elusimicrobia;o_UBA1565;f_UBA1565;g_UBA10166;s_UBA10166 sp001799695	89.47	0.36
Elusimicrobia bacterium NORP122	GCA_002401485.1	2.91	191	54.93	p_Elusimicrobiota;c_Elusimicrobia;o_UBA1565;f_UBA1565;g_UBA1565;s_UBA1565 sp002401485	91.61	1.29
Elusimicrobia bacterium UBA10166	GCA_003505215.1	4.76	305	68.17	p_Elusimicrobiota;c_Elusimicrobia;o_UBA1565;f_UBA1565;g_UBA10166;s_UBA10166 sp001799695	91.60	1.79
Elusimicrobia bacterium UBA1565	GCA_002322475.1	3.60	408	62.79	p_Elusimicrobiota;c_Elusimicrobia;o_UBA1565;f_UBA1565;g_UBA1565;s_UBA1565 sp002790095	91.17	5.22
Elusimicrobia bacterium UBA9639	GCA_003450735.1	4.17	84	67.93	p_Elusimicrobiota;c_Elusimicrobia;o_UBA1565;f_UBA1565;g_UBA9639;s_UBA9639 sp003450735	84.82	3.65
Elusimicrobium minutum Pei191	GCF_000020145.1	1.64	1	39.95	p_Elusimicrobiota;c_Elusimicrobia;o_Elusimicrobiales;f_Elusimicrobiaceae;g_Elusimicrobium;s_Elusimicrobium minutum	94.54	1.26
Endomicrobium proavitum Rsa215	GCF_001027545.1	1.59	1	39.34	p_Elusimicrobiota;c_Endomicrobia;o_Endomicrobiales;f_Endomicrobiaceae;g_Endomicrobium;s_Endomicrobium proavitum	50.53	2.33
Fibrobacteria bacterium nGRbin1	GCA_015232855.1	5.38	350	40.73	p_Fibrobacterota;c_Fibrobacteria;o_UBA11236;f_;g_;s_	78.15	8.39
Fusobacterium periodonticum ATCC 33693	GCA_000160475.1	2.62	53	27.37	p_Fusobacteriota;c_Fusobacteriia;o_Fusobacteriales;f_Fusobacteriaceae;g_Fusobacterium;s_Fusobacterium periodonticum	90.91	3.41
Fusobacterium russii ATCC 25533 593A	GCA_000381725.1	1.94	41	28.64	p_Fusobacteriota;c_Fusobacteriia;o_Fusobacteriales;f_Fusobacteriaceae;g_Fusobacterium;s_Fusobacterium russii	71.11	1.46
Gammaproteobacteria bacterium MAG_00150_gam_010	GCA_013349855.1	2.85	486	49.09	p_Proteobacteria;c_Gammaproteobacteria;o_GCF-002020875;f_GCF-002020875;g_NIOZ-UU100;s_NIOZ-UU100 sp013349825	97.97	2.58
Gammaproteobacteria bacterium MAG_00160_gam_009	GCA_013349845.1	2.90	318	49.11	p_Proteobacteria;c_Gammaproteobacteria;o_GCF-002020875;f_GCF-002020875;g_NIOZ-UU100;s_NIOZ-UU100 sp013349825	99.41	0.59
Gammaproteobacteria bacterium MAG_00172_gam_018	GCA_013349825.1	2.87	274	48.98	p_Proteobacteria;c_Gammaproteobacteria;o_GCF-002020875;f_GCF-002020875;g_NIOZ-UU100;s_NIOZ-UU100 sp013349825	99.40	0.00
Gammaproteobacteria bacterium MAG_00188_gam_006	GCA_013349835.1	2.66	565	48.83	p_Proteobacteria;c_Gammaproteobacteria;o_GCF-002020875;f_GCF-002020875;g_NIOZ-UU100;s_NIOZ-UU100 sp013349825	98.71	0.00
Gammaproteobacteria bacterium MAG_00212_gam_1	GCA_013349805.1	2.10	955	48.4	p_Proteobacteria;c_Gammaproteobacteria;o_GCF-002020875;f_GCF-002020875;g_NIOZ-UU100;s_NIOZ-UU100 sp013349825	99.35	0.00
Gammaproteobacteria bacterium MAG_00215_gam_020	GCA_013349775.1	2.93	507	49.02	p_Proteobacteria;c_Gammaproteobacteria;o_GCF-002020875;f_GCF-002020875;g_NIOZ-UU100;s_NIOZ-UU100 sp013349825	99.08	3.67
Gammaproteobacteria bacterium nER1bin10	GCA_015232955.1	2.60	485	54.7	p_Proteobacteria;c_Gammaproteobacteria;o_Thiohalomonadales;f_Thiohalomonadaceae;g_;s_	94.49	1.82
Gammaproteobacteria bacterium NORP174	GCA_002400775.1	1.50	52	46.59	p_Proteobacteria;c_Gammaproteobacteria;o_GCA-2400775;f_GCA-2400775;g_GCA-2400775;s_GCA-2400775 sp002400775	99.68	0.65
Gammaproteobacteria bacterium nPCRbin5	GCA_015231865.1	3.54	299	34.87	p_Proteobacteria;c_Gammaproteobacteria;o_GCF-002020875;f_GRL18;g_;s_	98.06	0.65
Gammaproteobacteria bacterium nTSbin2	GCA_015231435.1	2.87	223	52.9	p_Proteobacteria;c_Gammaproteobacteria;o_Thiohalomonadales;f_Thiohalomonadaceae;g_;s_	100.00	0.00
Gammaproteobacteria bacterium nS315bin38	GCA_015231665.1	2.47	429	62.18	p_Proteobacteria;c_Gammaproteobacteria;o_Chromatiales;f_Sedimenticolaceae;g_YD12-FULL-61-37;s_	99.35	1.29
Gemmata obscuriglobus UQM 2246	GCA_000171775.1	9.15	922	67.19	p_Planctomycetota;c_Planctomycetes;o_Gemmatales;f_Gemmataceae;g_Gemmata;s_Gemmata obscuriglobus	60.34	0.00
Geoalkalibacter ferrihydriticus DSM 17813	GCF_000820505.1	3.84	23	57.95	p_Desulfobacterota_F;c_Desulfuromonadia;o_Desulfuromonadales;f_Geoalkalibacteraceae;g_Geoalkalibacter;s_Geoalkalibacter ferrihydriticus	81.29	0.65
Geoalkalibacter subterraneus Red1	GCF_000827125.1	3.72	2	56.68	p_Desulfobacterota_F;c_Desulfuromonadia;o_Desulfuromonadales;f_Geoalkalibacteraceae;g_Geoalkalibacter_A;s_Geoalkalibacter_A subterraneus	84.82	0.67
Geobacter anodireducens SD-1	GCA_001628815.1	3.67	2	61.47	p_Desulfobacterota_F;c_Desulfuromonadia;o_Geobacterales;f_Geobacteraceae;g_Geobacter;s_Geobacter anodireducens	100.00	5.45
Geobacter daltonii FRC-32	GCF_000022265.1	4.30	1	53.47	p_Desulfobacterota_F;c_Desulfuromonadia;o_Geobacterales;f_Geobacteraceae;g_Geotalea;s_Geotalea daltonii	92.92	0.91
Geobacter lovleyi SZ	GCF_000020385.1	3.99	2	54.74	p_Desulfobacterota_F;c_Desulfuromonadia;o_Geobacterales;f_Pseudopelobacteraceae;g_Trichlorobacter;s_Trichlorobacter lovleyi	85.00	0.81
Geobacter metallireducens GS-15	GCF_000012925.1	4.01	2	59.49	p_Desulfobacterota_F;c_Desulfuromonadia;o_Geobacterales;f_Geobacteraceae;g_Geobacter;s_Geobacter metallireducens	95.24	0.00
Geobacter pelophilus Drf2	GCF_002117975.1	4.34	2	61.03	p_Desulfobacterota_F;c_Desulfuromonadia;o_Geobacterales;f_Geobacteraceae;g_Geomonas;s_Geomonas pelophila	95.85	3.18
Geobacter sp. L1geo	GCA_003574895.1	4.48	39	54.15	p_Desulfobacterota_F;c_Desulfuromonadia;o_Geobacterales;f_Pseudopelobacteraceae;g_Pseudopelobacter;s_Pseudopelobacter sp003574895	90.91	2.73
Geobacter sp. palsa_1151	GCA_003151775.1	2.95	145	56.96	p_Desulfobacterota_F;c_Desulfuromonadia;o_Geobacterales;f_Pseudopelobacteraceae;g_Pseudopelobacter;s_Pseudopelobacter sp003151775	95.35	3.64
Geobacter sp. UBA1603	GCA_002322035.1	2.35	108	56.78	p_Desulfobacterota_F;c_Desulfuromonadia;o_Geobacterales;f_Pseudopelobacteraceae;g_Pseudopelobacter;s_Pseudopelobacter sp002322035	92.92	4.55
Geobacter sp. UBA698	GCA_002298925.1	4.33	152	55.97	p_Desulfobacterota_F;c_Desulfuromonadia;o_Geobacterales;f_Pseudopelobacteraceae;g_Pseudopelobacter;s_Pseudopelobacter sp002298925	99.35	0.65
Geobacter sulfurreducens PCA	GCF_000007985.2	3.81	1	60.94	p_Desulfobacterota_F;c_Desulfuromonadia;o_Geobacterales;f_Geobacteraceae;g_Geobacter;s_Geobacter sulfurreducens	99.91	0.89
Geobacter thiogenes ATCC BAA-34	GCF_900167465.1	3.57	42	52.83	p_Desulfobacterota_F;c_Desulfuromonadia;o_Geobacterales;f_Pseudopelobacteraceae;g_Trichlorobacter;s_Trichlorobacter thiogenes	70.75	2.98
Geobacter toluenoxydans JCM 15764	GCA_001311985.1	4.21	77	53.59	p_Desulfobacterota_F;c_Desulfuromonadia;o_Geobacterales;f_Geobacteraceae;g_Geotalea;s_Geotalea toluenoxydans	94.94	2.78
Geobacter uraniireducens Rf4	GCF_000016745.1	5.14	1	54.24	p_Desulfobacterota_F;c_Desulfuromonadia;o_Geobacterales;f_Geobacteraceae;g_Geotalea;s_Geotalea uraniireducens	94.29	3.69
Geobacteraceae bacterium GWC2_53_11	GCA_001802645.1	4.28	91	53	p_Desulfobacterota_F;c_Desulfuromonadia;o_Geobacterales;f_Pseudopelobacteraceae;g_Pseudopelobacter;s_Pseudopelobacter sp001802645	91.55	7.83
Geobacteraceae bacterium GWC2_55_20	GCA_001802125.1	4.55	186	54.41	p_Desulfobacterota_F;c_Desulfuromonadia;o_Geobacterales;f_Pseudopelobacteraceae;g_Pseudopelobacter;s_Pseudopelobacter sp001802125	91.36	2.73
Geomonas oryzae S43	GCF_004117875.1	4.93	18	61.24	p_Desulfobacterota_F;c_Desulfuromonadia;o_Geobacterales;f_Geobacteraceae;g_Geomonas;s_Geomonas oryzae	95.85	2.83
Geothermobacter sp. EPR-M	GCF_002093115.1	3.73	59	59.39	p_Desulfobacterota_F;c_Desulfuromonadia;o_Desulfuromonadales;f_Geothermobacteraceae;g_PPFX01;s_PPFX01 sp002093115	95.59	3.28
Geothermobacter sp. HR-1	GCF_002898515.1	3.84	73	59.23	p_Desulfobacterota_F;c_Desulfuromonadia;o_Desulfuromonadales;f_Geothermobacteraceae;g_PPFX01;s_PPFX01 sp002898515	99.26	0.65

Genome name	NCBI/IMG accession	Size, Mbp	Scaff, no.	GC, %	GTDB Taxonomy	Completeness, %	Contamination, %
Ghiorsea bivora TAG-1	GCA_000744415.1	2.16	13	42.68	p__Proteobacteria;c__Zetaproteobacteria;o__Mariprofundales;f__Mariprofundaceae;g__Ghiorsea;s__Ghiorsea bivora	99.35	1.29
Gimesia maris DSM 8797	GCA_000181475.1	7.78	125	50.45	p__Planctomycetota;c__Planctomycetes;o__Planctomycetales;f__Planctomycetaceae;g__Gimesia;s__Gimesia maris	85.91	0.45
Halodesulfovibrio aestuarii DSM 17919	GCF_000384815.1	3.55	21	45.11	p__Desulfobacterota;c__Desulfovibrionia;o__Desulfovibrionales;f__Desulfovibrionaceae;g__Halodesulfovibrio;s__Halodesulfovibrio aestuarii	96.42	1.68
Halodesulfovibrio marinisediminis DSM 17456	GCF_900129975.1	3.71	13	44.96	p__Desulfobacterota;c__Desulfovibrionia;o__Desulfovibrionales;f__Desulfovibrionaceae;g__Halodesulfovibrio;s__Halodesulfovibrio marinisediminis	80.69	2.26
Halodesulfovibrio spirochaetisodalis JC271	GCF_001672295.1	3.61	59	46.19	p__Desulfobacterota;c__Desulfovibrionia;o__Desulfovibrionales;f__Desulfovibrionaceae;g__Halodesulfovibrio;s__Halodesulfovibrio spirochaetisodalis	95.80	2.52
Ilyobacter polytropus DSM 2926	GCA_000165505.1	3.13	3	34.37	p__Fusobacteriota;c__Fusobacteriia;o__Fusobacteriales;f__Fusobacteriaceae;g__Ilyobacter;s__Ilyobacter polytropus	89.68	5.59
Isosphaera pallida ATCC 43644	GCA_000186345.1	5.53	2	62.49	p__Planctomycetota;c__Planctomycetes;o__Isosphaerales;f__Isosphaeraceae;g__Isosphaera;s__Isosphaera pallida	66.54	0.00
Latescibacteria bacterium SCGC AAA252-B13	2264867252	1.76	138	40.86	p__Latescibacterota;c__Latescibacteria;o__Latescibacterales;f__Latescibacteraceae;g__Latescibacter;s__	43.99	0.00
Leptospirillum ferriphilum DSM 14647	GCF_000755505.1	2.41	18	54.05	p__Nitrospirota_A;c__Leptospirillia;o__Leptospirillales;f__Leptospirillaceae;g__Leptospirillum_A;s__Leptospirillum_A ferriphilum	68.81	0.62
Leptospirillum ferriphilum ML-04	GCF_000299235.1	2.41	1	54.55	p__Nitrospirota_A;c__Leptospirillia;o__Leptospirillales;f__Leptospirillaceae;g__Leptospirillum_A;s__Leptospirillum_A rubarum	98.58	0.91
Leptospirillum ferrooxidans C2-3	GCF_000284315.1	2.56	1	50.05	p__Nitrospirota_A;c__Leptospirillia;o__Leptospirillales;f__Leptospirillaceae;g__Leptospirillum;s__Leptospirillum ferrooxidans	55.23	0.00
Leptotrichia buccalis C-1013-b	GCA_000023905.1	2.47	1	29.65	p__Fusobacteriota;c__Fusobacteriia;o__Fusobacteriales;f__Leptotrichiaceae;g__Leptotrichia;s__Leptotrichia buccalis	62.13	5.10
Magnetococcales bacterium DC0425bin3	GCA_002753665.1	3.70	230	65.4	p__Proteobacteria;c__Magnetococcia;o__Magnetococcales;f__DC0425bin3;g__DC0425bin3;s__DC0425bin3 sp002753665	99.35	0.65
Magnetococcales bacterium DCbin2	GCA_002753615.1	3.36	201	51.85	p__Proteobacteria;c__Magnetococcia;o__Magnetococcales;f__UBA8363;g__UBA8363;s__UBA8363 sp002753615	99.40	0.00
Magnetococcales bacterium DCbin4	GCA_002753735.1	4.52	148	54.25	p__Proteobacteria;c__Magnetococcia;o__Magnetococcales;f__UBA8363;g__UBA8363;s__UBA8363 sp002753735	99.35	0.65
Magnetococcales bacterium DH2_bin20	GCA_011089865.1	5.01	525	52.96	p__Proteobacteria;c__Magnetococcia;o__Magnetococcales;f__;g__;s__	100.00	0.00
Magnetococcales bacterium DH2_bin6	GCA_011089965.1	3.66	80	56.76	p__Proteobacteria;c__Magnetococcia;o__Magnetococcales;f__Magnetaquicoccaceae;g__JAANAU01;s__JAANAU01 sp011089965	99.35	3.39
Magnetococcales bacterium ER1bin7	GCA_002753565.1	3.87	92	52.3	p__Proteobacteria;c__Magnetococcia;o__Magnetococcales;f__UBA8363;g__GCA-2753565;s__GCA-2753565 sp002753565	98.98	0.00
Magnetococcales bacterium HA3dbin1	GCA_002753515.1	4.33	134	53.32	p__Proteobacteria;c__Magnetococcia;o__Magnetococcales;f__UBA8363;g__UBA8363;s__UBA8363 sp002753515	100.00	0.00
Magnetococcales bacterium HA3dbin3	GCA_002753495.1	2.90	316	61.72	p__Proteobacteria;c__Magnetococcia;o__Magnetococcales;f__DC0425bin3;g__HA3dbin3;s__HA3dbin3 sp002753495	98.70	0.65
Magnetococcales bacterium HAa3bin1	GCA_002753595.1	4.35	118	53.21	p__Proteobacteria;c__Magnetococcia;o__Magnetococcales;f__UBA8363;g__UBA8363;s__UBA8363 sp002753515	99.50	1.24
Magnetococcales bacterium HCHbin5	GCA_002753505.1	4.19	200	56.97	p__Proteobacteria;c__Magnetococcia;o__Magnetococcales;f__Magnetaquicoccaceae;g__HCHbin5;s__HCHbin5 sp002753505	99.88	0.00
Magnetococcales bacterium MAG_21055_mgc_1	GCA_013349385.1	3.59	930	52.41	p__Proteobacteria;c__Magnetococcia;o__Magnetococcales;f__UBA8363;g__UBA8363;s__UBA8363 sp013349385	100.00	0.59
Magnetococcales bacterium nARSLQbin1	GCA_015234115.1	3.31	161	58.83	p__Proteobacteria;c__Magnetococcia;o__Magnetococcales;f__Magnetaquicoccaceae;g__JAANAU01;s__	99.50	0.00
Magnetococcales bacterium nCLbin10	GCA_015233785.1	3.36	364	55.62	p__Proteobacteria;c__Magnetococcia;o__Magnetococcales;f__;g__;s__	99.17	1.65
Magnetococcales bacterium nCLbin6	GCA_015234045.1	3.65	117	47.56	p__Proteobacteria;c__Magnetococcia;o__Magnetococcales;f__;g__;s__	100.00	0.00
Magnetococcales bacterium nDC0425bin2	GCA_015233945.1	3.04	364	55.86	p__Proteobacteria;c__Magnetococcia;o__Magnetococcales;f__Magnetaquicoccaceae;g__HCHbin5;s__HCHbin5 sp002753505	100.00	0.75
Magnetococcales bacterium nDC0425bin4	GCA_015233965.1	3.89	165	65.39	p__Proteobacteria;c__Magnetococcia;o__Magnetococcales;f__DC0425bin3;g__DC0425bin3;s__DC0425bin3 sp002753665	100.00	0.00
Magnetococcales bacterium nDCbin4	GCA_015234015.1	4.49	149	54.13	p__Proteobacteria;c__Magnetococcia;o__Magnetococcales;f__UBA8363;g__UBA8363;s__UBA8363 sp002753735	99.35	1.61
Magnetococcales bacterium nDH2bin6	GCA_015233935.1	4.54	414	52.7	p__Proteobacteria;c__Magnetococcia;o__Magnetococcales;f__;g__;s__	99.41	0.00
Magnetococcales bacterium nDH2bin7	GCA_015233905.1	1.95	74	56.31	p__Proteobacteria;c__Magnetococcia;o__Magnetococcales;f__Magnetaquicoccaceae;g__JAANAU01;s__JAANAU01 sp011089965	100.00	1.39
Magnetococcales bacterium nDJH13bin19	GCA_015233895.1	3.71	65	41.57	p__Nitrospirota;c__Thermodesulfovibrionia;o__Thermodesulfovibrionales;f__Magnetobacteriaceae;g__;s__	79.81	1.71
Magnetococcales bacterium nDJH15bin4	GCA_015233515.1	4.20	701	51.2	p__Desulfobacterota;c__Desulfarculia_A;o__Adiutricales;f__;g__;s__	94.87	3.85
Magnetococcales bacterium nDJH8bin2	GCA_015233035.1	4.53	434	57.45	p__Proteobacteria;c__Magnetococcia;o__Magnetococcales;f__Magnetaquicoccaceae;g__HCHbin5;s__	98.82	0.30
Magnetococcales bacterium nER1bin1	GCA_015232915.1	3.66	47	52.32	p__Proteobacteria;c__Magnetococcia;o__Magnetococcales;f__UBA8363;g__GCA-2753565;s__GCA-2753565 sp002753565	98.51	0.50
Magnetococcales bacterium nER1bin6	GCA_015232865.1	3.09	102	51.04	p__Proteobacteria;c__Magnetococcia;o__Magnetococcales;f__UBA8363;g__GCA-2753565;s__	99.41	0.07
Magnetococcales bacterium nGRbin4	GCA_015232795.1	2.87	466	42	p__Proteobacteria;c__Magnetococcia;o__Magnetococcales;f__UBA8363;g__GCA-2753565;s__	100.00	0.00
Magnetococcales bacterium nHA1bin2	GCA_015232825.1	3.03	270	50.29	p__Proteobacteria;c__Magnetococcia;o__Magnetococcales;f__Magnetaquicoccaceae;g__JAANAU01;s__	99.35	2.15
Magnetococcales bacterium nHA3dbin1	GCA_015232815.1	4.47	203	52.89	p__Proteobacteria;c__Magnetococcia;o__Magnetococcales;f__UBA8363;g__UBA8363;s__UBA8363 sp002753515	99.35	0.65
Magnetococcales bacterium nHA3dbin2	GCA_015232755.1	3.55	358	61.56	p__Proteobacteria;c__Magnetococcia;o__Magnetococcales;f__DC0425bin3;g__HA3dbin3;s__HA3dbin3 sp002753495	99.33	0.00
Magnetococcales bacterium nHA4bin1	GCA_015232765.1	2.44	414	50.34	p__Proteobacteria;c__Magnetococcia;o__Magnetococcales;f__Magnetaquicoccaceae;g__JAANAU01;s__	99.35	0.65
Magnetococcales bacterium nHA4bin8	GCA_015232705.1	2.05	382	58.19	p__Proteobacteria;c__Magnetococcia;o__Magnetococcales;f__Magnetaquicoccaceae;g__JAANAU01;s__	99.70	0.00
Magnetococcales bacterium nHA5abin2	GCA_015232685.1	3.24	229	52.36	p__Proteobacteria;c__Magnetococcia;o__Magnetococcales;f__UBA8363;g__UBA8363;s__UBA8363 sp002753515	99.68	0.00
Magnetococcales bacterium nHA5abin3	GCA_015232645.1	3.39	426	54.67	p__Proteobacteria;c__Magnetococcia;o__Magnetococcales;f__UBA8363;g__UBA8363;s__	100.00	0.75
Magnetococcales bacterium nHAa3bin1	GCA_015232675.1	3.00	97	53.21	p__Proteobacteria;c__Magnetococcia;o__Magnetococcales;f__UBA8363;g__UBA8363;s__UBA8363 sp002753515	98.71	0.00
Magnetococcales bacterium nHCHbin1	GCA_015232635.1	3.81	295	56.77	p__Proteobacteria;c__Magnetococcia;o__Magnetococcales;f__Magnetaquicoccaceae;g__HCHbin5;s__HCHbin5 sp002753505	100.00	1.09
Magnetococcales bacterium nJSWbin1	GCA_015232405.1	5.86	335	42.2	p__Proteobacteria;c__Magnetococcia;o__Magnetococcales;f__UBA8363;g__GCA-2753565;s__	100.00	0.00
Magnetococcales bacterium nJSWbin2	GCA_015232395.1	4.94	170	55.33	p__Proteobacteria;c__Magnetococcia;o__Magnetococcales;f__;g__;s__	99.35	0.00
Magnetococcales bacterium nJSWbin3	GCA_015232365.1	2.84	103	44.6	p__Proteobacteria;c__Magnetococcia;o__Magnetococcales;f__UBA8363;g__GCA-2753565;s__	99.45	0.82
Magnetococcales bacterium nKLKbin4	GCA_015232265.1	4.03	225	54.24	p__Proteobacteria;c__Magnetococcia;o__Magnetococcales;f__UBA8363;g__UBA8363;s__UBA8363 sp002753735	100.00	0.62
Magnetococcales bacterium nMBPbin6	GCA_015232245.1	3.70	481	59.23	p__Proteobacteria;c__Magnetococcia;o__Magnetococcales;f__Magnetaquicoccaceae;g__JAANAU01;s__	94.17	2.73
Magnetococcales bacterium nMYbin5	GCA_015232135.1	2.25	376	56.15	p__Proteobacteria;c__Magnetococcia;o__Magnetococcales;f__DC0425bin3;g__HA3dbin3;s__	99.35	0.65
Magnetococcales bacterium nNGHbin13	GCA_015231965.1	3.82	314	63.09	p__Proteobacteria;c__Magnetococcia;o__;f__;g__;s__	99.50	0.25
Magnetococcales bacterium nNGHbin14	GCA_015231925.1	4.51	358	60.22	p__Proteobacteria;c__Magnetococcia;o__Magnetococcales;f__;g__;s__	100.00	0.20
Magnetococcales bacterium nNGHbin2	GCA_015231915.1	4.02	133	61.98	p__Proteobacteria;c__Magnetococcia;o__Magnetococcales;f__Magnetaquicoccaceae;g__WMHbin3;s__	96.82	4.77
Magnetococcales bacterium nQXH1bin1	GCA_015231795.1	3.63	23	59.95	p__Proteobacteria;c__Alphaproteobacteria;o__Rhodospirillales;f__WMHbin7;g__WMHbin7;s__	99.42	0.00
Magnetococcales bacterium nQXH2bin1	GCA_015231775.1	3.82	389	61.35	p__Proteobacteria;c__Magnetococcia;o__Magnetococcales;f__Magnetaquicoccaceae;g__JAANAU01;s__	96.13	0.65
Magnetococcales bacterium nQXH2bin5	GCA_015231755.1	3.84	44	59.16	p__Proteobacteria;c__Magnetococcia;o__Magnetococcales;f__Magnetaquicoccaceae;g__JAANAU01;s__	99.17	0.00
Magnetococcales bacterium nW5bin1	GCA_015231375.1	4.10	114	59.91	p__Proteobacteria;c__Magnetococcia;o__Magnetococcales;f__Magnetaquicoccaceae;g__JAANAU01;s__	92.42	0.91

Genome name	NCBI/IMG accession	Size, Mbp	Scaff, no.	GC, %	GTDB Taxonomy	Completeness, %	Contamination, %
Magnetococcales bacterium nW5bin3	GCA_015231295.1	3.79	353	59.17	p_Proteobacteria;c_Magnetococcia;o_Magnetococcales;f_Magnetaquicoccaceae;g_JAANAU01;s_	98.74	0.00
Magnetococcales bacterium nwagbin5	GCA_015231305.1	3.96	238	38.49	p_Proteobacteria;c_Magnetococcia;o_Magnetococcales;f_UBA8363;g_GCA-2753565;s_	96.75	4.27
Magnetococcales bacterium nwalbin5	GCA_015231265.1	3.82	459	55.87	p_Proteobacteria;c_Magnetococcia;o_;f_;g_;s_	99.35	1.29
Magnetococcales bacterium nWMHbin1	GCA_015231215.1	4.37	116	54.97	p_Proteobacteria;c_Magnetococcia;o_Magnetococcales;f_Magnetaquicoccaceae;g_Magnetaquicoccus;s_Magnetaquicoccus sp002753135	97.66	0.71
Magnetococcales bacterium nWMHbin2	GCA_015231175.1	3.81	175	57.17	p_Proteobacteria;c_Magnetococcia;o_Magnetococcales;f_DC0425bin3;g_HA3dbin3;s_	100.00	0.00
Magnetococcales bacterium nWMHbin3	GCA_015231235.1	4.97	167	61.57	p_Proteobacteria;c_Magnetococcia;o_Magnetococcales;f_Magnetaquicoccaceae;g_WMHbin3;s_WMHbin3 sp002753185	100.00	0.91
Magnetococcales bacterium nWMHbin4	GCA_015231205.1	4.16	207	54.29	p_Proteobacteria;c_Magnetococcia;o_Magnetococcales;f_UBA8363;g_UBA8363;s_UBA8363 sp002753735	99.35	2.26
Magnetococcales bacterium nWRX1bin1	GCA_015229095.1	2.63	200	59.27	p_Proteobacteria;c_Magnetococcia;o_Magnetococcales;f_Magnetaquicoccaceae;g_HCHbin5;s_	100.00	0.00
Magnetococcales bacterium nWRX1bin11	GCA_015229135.1	3.56	254	60.94	p_Proteobacteria;c_Magnetococcia;o_Magnetococcales;f_Magnetaquicoccaceae;g_JAANAU01;s_	100.00	0.00
Magnetococcales bacterium nWRX1bin12	GCA_015229105.1	2.77	199	62.96	p_Proteobacteria;c_Magnetococcia;o_Magnetococcales;f_;g_;s_	95.27	1.78
Magnetococcales bacterium nWRX1bin3	GCA_015229115.1	3.15	184	54.88	p_Proteobacteria;c_Magnetococcia;o_Magnetococcales;f_;g_;s_	100.00	0.07
Magnetococcales bacterium nWRX1bin5	GCA_015229055.1	4.55	173	54.4	p_Proteobacteria;c_Magnetococcia;o_Magnetococcales;f_UBA8363;g_UBA8363;s_UBA8363 sp002753735	100.00	1.19
Magnetococcales bacterium nWRX1bin6	GCA_015229045.1	4.08	283	57.46	p_Proteobacteria;c_Magnetococcia;o_Magnetococcales;f_DC0425bin3;g_HA3dbin3;s_	98.11	0.00
Magnetococcales bacterium nWRX2bin6	GCA_015229035.1	4.72	210	54.25	p_Proteobacteria;c_Magnetococcia;o_Magnetococcales;f_UBA8363;g_UBA8363;s_UBA8363 sp002753735	99.91	0.07
Magnetococcales bacterium nWRX3bin10	GCA_015229005.1	3.28	173	62.87	p_Proteobacteria;c_Magnetococcia;o_Magnetococcales;f_;g_;s_	99.96	0.00
Magnetococcales bacterium nWRX3bin11	GCA_015228995.1	1.96	287	53.76	p_Proteobacteria;c_Magnetococcia;o_Magnetococcales;f_;g_;s_	99.50	0.50
Magnetococcales bacterium nWRX3bin12	GCA_015228975.1	3.75	234	52.93	p_Proteobacteria;c_Magnetococcia;o_Magnetococcales;f_UBA8363;g_UBA8363;s_	99.35	0.65
Magnetococcales bacterium nWRX3bin2	GCA_015228935.1	4.71	197	54.61	p_Proteobacteria;c_Magnetococcia;o_Magnetococcales;f_DC0425bin3;g_HA3dbin3;s_	99.17	0.62
Magnetococcales bacterium nWRX3bin6	GCA_015228925.1	4.25	219	52.02	p_Proteobacteria;c_Magnetococcia;o_Magnetococcales;f_UBA8363;g_UBA8363;s_	100.00	0.78
Magnetococcales bacterium nWRX3bin7	GCA_015228895.1	2.89	501	61.11	p_Proteobacteria;c_Magnetococcia;o_Magnetococcales;f_Magnetaquicoccaceae;g_WMHbin3;s_	94.35	2.80
Magnetococcales bacterium nYD0423bin2	GCA_015228815.1	4.22	97	56.86	p_Proteobacteria;c_Magnetococcia;o_Magnetococcales;f_UBA8363;g_UBA8363;s_	98.39	1.94
Magnetococcales bacterium nYD0423bin3	GCA_015228775.1	4.50	146	55.48	p_Proteobacteria;c_Magnetococcia;o_Magnetococcales;f_Magnetaquicoccaceae;g_Magnetaquicoccus;s_Magnetaquicoccus sp002753095	98.06	4.54
Magnetococcales bacterium nYD0425bin13	GCA_015228825.1	4.22	231	55.36	p_Proteobacteria;c_Magnetococcia;o_Magnetococcales;f_Magnetaquicoccaceae;g_Magnetaquicoccus;s_Magnetaquicoccus sp002753095	95.91	6.55
Magnetococcales bacterium WMHbin1	GCA_002753215.1	4.38	242	54.3	p_Proteobacteria;c_Magnetococcia;o_Magnetococcales;f_UBA8363;g_UBA8363;s_UBA8363 sp002753735	100.00	0.00
Magnetococcales bacterium WMHbin3	GCA_002753185.1	4.60	157	61.61	p_Proteobacteria;c_Magnetococcia;o_Magnetococcales;f_Magnetaquicoccaceae;g_WMHbin3;s_WMHbin3 sp002753185	96.76	2.73
Magnetococcales bacterium WMHbinv6	GCA_002753135.1	3.84	80	55.32	p_Proteobacteria;c_Magnetococcia;o_Magnetococcales;f_Magnetaquicoccaceae;g_Magnetaquicoccus;s_Magnetaquicoccus sp002753135	99.03	3.87
Magnetococcales bacterium YD0425bin7	GCA_002753095.1	3.58	177	55.67	p_Proteobacteria;c_Magnetococcia;o_Magnetococcales;f_Magnetaquicoccaceae;g_Magnetaquicoccus;s_Magnetaquicoccus sp002753095	95.51	0.37
Magnetococcus marinus MC-1	GCA_000014865.1	4.72	1	54.17	p_Proteobacteria;c_Magnetococcia;o_Magnetococcales;f_Magnetococcaceae;g_Magnetococcus;s_Magnetococcus marinus	97.94	1.13
Magnetofaba australis IT-1	GCA_002109495.1	4.99	21	61.3	p_Proteobacteria;c_Magnetococcia;o_Magnetococcales;f_Magnetococcaceae;g_Magnetofaba;s_Magnetofaba australis	99.41	0.00
Magnetospira sp. QH-2	GCF_000968135.1	4.05	2	59.44	p_Proteobacteria;c_Alphaproteobacteria;o_Rhodospirillales;f_Magnetospiraceae;g_Magnetospira;s_Magnetospira sp000968135	98.32	1.68
Magnetospirillum caucaseum SO-1	GCF_000342045.1	4.86	236	65.99	p_Proteobacteria;c_Alphaproteobacteria;o_Rhodospirillales;f_Magnetospirillaceae;g_Phaeospirillum;s_Phaeospirillum caucaseum	96.06	2.73
Magnetospirillum gryphiswaldense MSR-1 v2	GCF_000513295.1	4.37	1	63.28	p_Proteobacteria;c_Alphaproteobacteria;o_Rhodospirillales;f_Magnetospirillaceae;g_Magnetospirillum;s_Magnetospirillum gryphiswaldense	83.95	3.23
Magnetospirillum kuznetsovii LBB-42	GCA_003284725.1	4.41	69	63.44	p_Proteobacteria;c_Alphaproteobacteria;o_Rhodospirillales;f_Magnetospirillaceae;g_Phaeospirillum;s_Phaeospirillum kuznetsovii	99.94	1.82
Magnetospirillum magneticum AMB-1	GCF_000009985.1	4.97	1	65.09	p_Proteobacteria;c_Alphaproteobacteria;o_Rhodospirillales;f_Magnetospirillaceae;g_Phaeospirillum;s_Phaeospirillum magneticum	97.05	2.58
Magnetospirillum magnetotacticum MS-1	GCF_000829825.1	4.52	36	63.56	p_Proteobacteria;c_Alphaproteobacteria;o_Rhodospirillales;f_Magnetospirillaceae;g_Phaeospirillum;s_Phaeospirillum magnetotacticum	99.35	1.29
Magnetospirillum marisnigri SP-1	GCF_001650715.1	4.62	131	64.73	p_Proteobacteria;c_Alphaproteobacteria;o_Rhodospirillales;f_Magnetospirillaceae;g_Phaeospirillum;s_Phaeospirillum marisnigri	98.06	2.74
Magnetospirillum moscoviense BB-1	GCF_001650635.1	4.16	207	65.18	p_Proteobacteria;c_Alphaproteobacteria;o_Rhodospirillales;f_Magnetospirillaceae;g_Magnetospirillum;s_Magnetospirillum moscoviense	70.17	0.00
Magnetovibrio blakemorei MV-1	GCF_001746755.1	3.64	91	54.29	p_Proteobacteria;c_Alphaproteobacteria;o_Rhodospirillales;f_Magnetovibrionaceae;g_Magnetovibrio;s_Magnetovibrio blakemorei	67.54	1.75
Magnetovibrio sp. ARS8	GCA_002686765.1	2.02	197	59.64	p_Proteobacteria;c_Alphaproteobacteria;o_Rhodospirillales;f_2-02-FULL-58-16;g_GCA-2686765;s_GCA-2686765 sp002686765	99.03	0.91
Mailhella massiliensis Marseille-P3199	GCF_900155525.1	3.47	28	59.08	p_Desulfobacterota;c_Desulfovibrionia;o_Desulfovibrionales;f_Desulfovibrionaceae;g_Mailhella;s_Mailhella massiliensis	98.32	2.94
Malonomonas rubra DSM 5091	GCF_900142125.1	3.96	42	52.46	p_Desulfobacterota_F;c_Desulfuromonadia;o_Desulfuromonadales;f_Geopsychrobacteraceae;g_Malonomonas;s_Malonomonas rubra	70.43	2.27
Malonomonas rubra SZUA-375	GCA_003247215.1	3.13	149	52.49	p_Desulfobacterota_F;c_Desulfuromonadia;o_Desulfuromonadales;f_Geopsychrobacteraceae;g_Malonomonas;s_Malonomonas rubra_A	56.35	0.97
Mariprofundus ferrinatatus CP-8	GCA_002795825.1	2.30	1	53.66	p_Proteobacteria;c_Zetaproteobacteria;o_Mariprofundales;f_Mariprofundaceae;g_Mariprofundus;s_Mariprofundus ferrinatatus	100.00	1.45
Mariprofundus ferrooxydans M34	GCA_000379405.1	2.74	36	53.93	p_Proteobacteria;c_Zetaproteobacteria;o_Mariprofundales;f_Mariprofundaceae;g_Mariprofundus;s_Mariprofundus ferrooxydans	100.00	0.46
Mariprofundus micogutta ET2	GCA_001895085.1	2.50	59	48.76	p_Proteobacteria;c_Zetaproteobacteria;o_Mariprofundales;f_Mariprofundaceae;g_Mariprofundus;s_Mariprofundus micogutta	99.39	0.00
Nitrospina gracilis 3211	GCA_000341545.2	3.08	4	56.21	p_Nitrospinota;c_Nitrospinia;o_Nitrospinales;f_Nitrospinaceae;g_Nitrospina;s_Nitrospina gracilis	85.45	0.50
Nitrospina sp. NAT278	GCA_002697825.1	1.78	90	37.06	p_Nitrospinota;c_Nitrospinia;o_Nitrospinales;f_Nitrospinaceae;g_UBA8687;s_UBA8687 sp002697825	55.59	2.41
Nitrospina sp. SCGC AAA288-L16	GCA_000372225.1	2.08	136	39.47	p_Nitrospinota;c_Nitrospinia;o_Nitrospinales;f_Nitrospinaceae;g_SCGCAAA288-L16;s_SCGCAAA288-L16 sp000372225	89.28	5.86
Nitrospina sp. SP265	GCA_002721515.1	1.64	51	36.86	p_Nitrospinota;c_Nitrospinia;o_Nitrospinales;f_Nitrospinaceae;g_UBA8687;s_UBA8687 sp002721515	95.73	5.34
Nitrospinae bacterium bin107	GCA_002238965.1	4.37	60	59.46	p_Nitrospinota_A;c_UBA8248;o_UBA8248;f_UBA8248;g_Bin107;s_Bin107 sp002238965	60.69	3.87
Nitrospinae bacterium MAG_09705_ntspn_70	GCA_013349585.1	2.02	120	42.63	p_Nitrospinota;c_Nitrospinia;o_Nitrospinales;f_Nitrospinaceae;g_UBA8687;s_UBA8687 sp013349585	78.43	5.08
Nitrospinae bacterium nARSLQbin3	GCA_015234085.1	2.64	440	54.88	p_Nitrospinota;c_UBA7883;o_UBA7883;f_;g_;s_	98.17	3.96
Nitrospinae bacterium nNGHbin12	GCA_015231995.1	3.23	358	54.84	p_Nitrospinota;c_UBA7883;o_UBA7883;f_;g_;s_	99.39	4.88
Nitrospinae bacterium NP1026	GCA_002731985.1	1.58	114	46.05	p_Nitrospinota;c_Nitrospinia;o_Nitrospinales;f_Nitrospinaceae;g_SCGCAAA288-L16;s_SCGCAAA288-L16 sp002731985	87.64	2.12
Nitrospinae bacterium nPCRbin9	GCA_015231815.1	2.65	410	37.05	p_Nitrospinota;c_UBA7883;o_;f_;g_;s_	96.95	3.05
Nitrospinae bacterium nWMHbin6	GCA_015229165.1	2.31	346	63.95	p_Nitrospinota;c_UBA7883;o_UBA7883;f_;g_;s_	95.12	4.19
Nitrospira bacterium HGW-Nitrospira-1	GCA_002839535.1	1.66	182	46.15	p_Nitrospirota;c_Thermodesulfovibrionia;o_Thermodesulfovibrionales;f_UBA6898;g_GW-Nitrospira-1;s_GW-Nitrospira-1 sp002839535	84.69	0.50
Nitrospira defluvii	GCA_000196815.1	4.32	1	59.03	p_Nitrospirota;c_Nitrospiria;o_Nitrospirales;f_Nitrospiraceae;g_Nitrospira_A;s_Nitrospira_A defluvii_A	93.50	2.19
Nitrospira japonica NJ11	GCF_900169565.1	4.08	1	58.96	p_Nitrospirota;c_Nitrospiria;o_Nitrospirales;f_Nitrospiraceae;g_Nitrospira_C;s_Nitrospira_C japonica	79.91	1.17

Genome name	NCBI/IMG accession	Size, Mbp	Scaff, no.	GC, %	GTDB Taxonomy	Completeness, %	Contamination, %
Nitrospira lenta BS10	GCF_900403705.1	3.76	22	57.88	p_Nitrospirota;c_Nitrospiria;o_Nitrospirales;f_Nitrospiraceae;g_Nitrospira_D;s_Nitrospira_D lenta	98.10	1.26
Nitrospira moscoviensis NSP M-1	GCF_001273775.1	4.59	1	61.99	p_Nitrospirota;c_Nitrospiria;o_Nitrospirales;f_Nitrospiraceae;g_Nitrospira_E;s_Nitrospira_E moscoviensis	61.21	1.72
Nitrospira sp. Bin_34_1	GCA_005239745.1	1.63	145	52.91	p_Nitrospirota;c_Nitrospiria;o_SBBL01;f_SBBL01;g_SBBL01;s_SBBL01 sp005239745	73.76	1.03
Nitrospira sp. Bin_6_1_1	GCA_005239595.1	1.88	236	50.25	p_Nitrospirota;c_Nitrospiria;o_SBBL01;f_SBBT01;g_SBBT01;s_SBBT01 sp005239595	90.84	1.79
Nitrospira sp. bin75	GCA_002238765.1	3.04	115	56.24	p_Nitrospirota;c_Nitrospiria;o_Nitrospirales;f_UBA8639;g_Bin75;s_Bin75 sp002238765	92.43	0.44
Nitrospira sp. CG24A	GCA_002869925.2	3.36	40	55.68	p_Nitrospirota;c_Nitrospiria;o_Nitrospirales;f_Nitrospiraceae;g_Palsa-1315;s_Palsa-1315 sp002869925	96.89	2.94
Nitrospira sp. CG24B	GCA_002869845.2	3.14	20	55.28	p_Nitrospirota;c_Nitrospiria;o_Nitrospirales;f_Nitrospiraceae;g_Nitrospira_F;s_Nitrospira_F sp002869845	94.12	2.10
Nitrospira sp. CG24C	GCA_002869885.2	2.99	22	56.12	p_Nitrospirota;c_Nitrospiria;o_Nitrospirales;f_Nitrospiraceae;g_Palsa-1315;s_Palsa-1315 sp002869885	97.48	2.10
Nitrospira sp. CG24D	GCA_002869855.2	3.41	87	57.84	p_Nitrospirota;c_Nitrospiria;o_Nitrospirales;f_Nitrospiraceae;g_Nitrospira_D;s_Nitrospira_D sp002869855	93.70	0.05
Nitrospira sp. CG24E	GCA_002869895.2	3.45	99	55.84	p_Nitrospirota;c_Nitrospiria;o_Nitrospirales;f_Nitrospiraceae;g_Palsa-1315;s_Palsa-1315 sp002869895	96.69	2.63
Nitrospira sp. ND1	GCF_900170025.1	4.45	6	58.87	p_Nitrospirota;c_Nitrospiria;o_Nitrospirales;f_Nitrospiraceae;g_Nitrospira_A;s_Nitrospira_A sp900170025	81.68	0.50
Nitrospira sp. OLB3	GCA_001567445.1	3.75	79	60.36	p_Nitrospirota;c_Nitrospiria;o_Nitrospirales;f_Nitrospiraceae;g_Nitrospira_A;s_Nitrospira_A sp001567445	96.77	0.52
Nitrospira sp. palsa_1310	GCA_003135435.1	4.34	254	56.94	p_Nitrospirota;c_Nitrospiria;o_Nitrospirales;f_Nitrospiraceae;g_Palsa-1315;s_Palsa-1315 sp003135435	78.85	1.59
Nitrospira sp. RCA	GCA_005239465.1	3.30	85	56.83	p_Nitrospirota;c_Nitrospiria;o_Nitrospirales;f_Nitrospiraceae;g_Nitrospira_F;s_Nitrospira_F sp005239465	73.74	0.85
Nitrospira sp. RCB	GCA_005239475.1	3.56	289	57.07	p_Nitrospirota;c_Nitrospiria;o_Nitrospirales;f_Nitrospiraceae;g_Palsa-1315;s_Palsa-1315 sp005239475	60.70	0.00
Nitrospira sp. RSF1	GCA_005116965.1	3.73	27	55.24	p_Nitrospirota;c_Nitrospiria;o_Nitrospirales;f_Nitrospiraceae;g_Nitrospira_F;s_Nitrospira_F sp005116965	75.33	5.04
Nitrospira sp. RSF12	GCA_005116955.1	3.65	8	55.15	p_Nitrospirota;c_Nitrospiria;o_Nitrospirales;f_Nitrospiraceae;g_Nitrospira_F;s_Nitrospira_F sp005116955	91.04	3.36
Nitrospira sp. RSF3	GCA_005116835.1	3.94	24	55.56	p_Nitrospirota;c_Nitrospiria;o_Nitrospirales;f_Nitrospiraceae;g_Palsa-1315;s_Palsa-1315 sp005116835	88.72	0.84
Nitrospira sp. RSF5	GCA_005116895.1	3.31	35	55.18	p_Nitrospirota;c_Nitrospiria;o_Nitrospirales;f_Nitrospiraceae;g_Nitrospira_F;s_Nitrospira_F sp005116895	58.40	1.26
Nitrospira sp. RSF6	GCA_005116885.1	3.19	49	55.43	p_Nitrospirota;c_Nitrospiria;o_Nitrospirales;f_Nitrospiraceae;g_Palsa-1315;s_Palsa-1315 sp005116885	87.17	1.26
Nitrospira sp. RSF9	GCA_005116745.1	3.86	32	54.96	p_Nitrospirota;c_Nitrospiria;o_Nitrospirales;f_Nitrospiraceae;g_Nitrospira_F;s_Nitrospira_F sp005116745	75.18	1.68
Nitrospira sp. Ru_enrich_NS	GCA_002634385.1	4.31	77	55.93	p_Nitrospirota;c_Thermodesulfovibrionia;o_Thermodesulfovibrionales;f_UBA9935;g_GCA-2634385;s_GCA-2634385 sp002634385	94.79	2.10
Nitrospira sp. SG-bin1	GCA_002083365.1	4.42	48	56.08	p_Nitrospirota;c_Nitrospiria;o_Nitrospirales;f_Nitrospiraceae;g_Nitrospira_F;s_Nitrospira_F sp002083365	95.63	0.00
Nitrospira sp. SG-bin2	GCA_002083405.1	3.66	63	56.77	p_Nitrospirota;c_Nitrospiria;o_Nitrospirales;f_Nitrospiraceae;g_Nitrospira_F;s_Nitrospira_F sp002083405	78.88	3.85
Nitrospira sp. ST-bin4	GCA_002083565.1	2.94	117	57.02	p_Nitrospirota;c_Nitrospiria;o_Nitrospirales;f_Nitrospiraceae;g_Nitrospira_F;s_Nitrospira_F sp002083565	65.43	0.00
Nitrospira sp. ST-bin5	GCA_002083555.1	4.01	29	58.02	p_Nitrospirota;c_Nitrospiria;o_Nitrospirales;f_Nitrospiraceae;g_Nitrospira_D;s_Nitrospira_D sp002083555	57.14	0.00
Nitrospira sp. UBA2082	GCA_002331335.1	3.56	37	57.89	p_Nitrospirota;c_Nitrospiria;o_Nitrospirales;f_Nitrospiraceae;g_Nitrospira_F;s_Nitrospira_F sp002331335	97.48	2.10
Nitrospira sp. UBA5698	GCA_002420115.1	4.38	73	55.06	p_Nitrospirota;c_Nitrospiria;o_Nitrospirales;f_Nitrospiraceae;g_Nitrospira_F;s_Nitrospira_F sp002420115	83.19	5.04
Nitrospira sp. UBA5699	GCA_002420105.1	3.44	124	62.41	p_Nitrospirota;c_Nitrospiria;o_Nitrospirales;f_Nitrospiraceae;g_Nitrospira_C;s_Nitrospira_C sp002420105	96.64	2.10
Nitrospira sp. UW-LDO-01	GCA_002254365.1	3.91	230	54.93	p_Nitrospirota;c_Nitrospiria;o_Nitrospirales;f_Nitrospiraceae;g_Nitrospira_F;s_Nitrospira_F sp002254365	58.62	0.00
Nitrospiraceae bacterium fen_1308	GCA_003170655.1	2.83	254	49.38	p_Nitrospirota;c_Thermodesulfovibrionia;o_Thermodesulfovibrionales;f_UBA9935;g_Fen-1308;s_Fen-1308 sp003170655	88.29	2.15
Nitrospiraceae bacterium SURF_11	GCA_003599505.1	2.64	87	47.98	p_Nitrospirota;c_Thermodesulfovibrionia;o_UBA6902;f_UBA6902;g_SURF-11;s_SURF-11 sp003599505	76.53	3.36
Nitrospiraceae bacterium SURF_45	GCA_003599275.1	3.05	74	47.56	p_Nitrospirota;c_Thermodesulfovibrionia;o_UBA6902;f_UBA6902;g_SURF-45;s_SURF-45 sp003599275	88.69	1.10
Nitrospiraceae bacterium UBA2194	GCA_002328665.1	2.20	132	47.95	p_Nitrospirota;c_Thermodesulfovibrionia;o_Thermodesulfovibrionales;f_SM23-35;g_UBA2194;s_UBA2194 sp002328665	90.48	0.00
Nitrospiraceae bacterium UBA2600	GCA_002339325.1	1.90	53	36.78	p_Nitrospirota;c_Thermodesulfovibrionia;o_Thermodesulfovibrionales;f_Thermodesulfovibrionaceae;g_Thermodesulfovibrio;s__sp002339325	90.55	1.68
Nitrospiraceae bacterium UBA665	GCA_002299835.1	2.02	173	40.35	p_Nitrospirota;c_Thermodesulfovibrionia;o_Thermodesulfovibrionales;f_UBA9935;g_UBA665;s_UBA665 sp002299835	86.97	3.78
Nitrospiraceae bacterium UBA6902	GCA_002451135.1	3.05	160	46.5	p_Nitrospirota;c_Thermodesulfovibrionia;o_UBA6902;f_UBA6902;g_UBA6902;s_UBA6902 sp002451135	73.79	1.72
Nitrospiraceae bacterium UBA9159	GCA_003453735.1	4.66	540	48.26	p_Nitrospirota;c_Thermodesulfovibrionia;o_Thermodesulfovibrionales;f_UBA9159;g_UBA9159;s_UBA9159 sp003453735	95.65	1.97
Nitrospiraceae bacterium UBA9217	GCA_003454665.1	3.01	388	54.65	p_Nitrospirota;c_UBA9217;o_UBA9217;f_UBA9217;g_UBA9217;s_UBA9217 sp003454665	73.13	0.97
Nitrospirae bacterium Baikal-G1	GCA_002737345.1	1.67	38	57.92	p_Nitrospirota;c_Nitrospiria;o_Nitrospirales;f_Nitrospiraceae;g_Palsa-1315;s_Palsa-1315 sp002737345	95.38	0.84
Nitrospirae bacterium BMS3Abin08	GCA_002897935.1	2.61	123	47.88	p_Nitrospirota;c_Thermodesulfovibrionia;o_Thermodesulfovibrionales;f_JdFR-85;g_BMS3Abin08;s_BMS3Abin08 sp002897935	96.77	1.75
Nitrospirae bacterium BMS3Bbin05	GCA_002897855.1	2.46	89	44.67	p_Nitrospirota;c_Thermodesulfovibrionia;o_Thermodesulfovibrionales;f_BMS3Bbin05;g_BMS3Bbin05;s_BMS3Bbin05 sp002897855	94.65	0.33
Nitrospirae bacterium BMS3Bbin08	GCA_002897775.1	2.71	67	45.24	p_Nitrospirota;c_Thermodesulfovibrionia;o_UBA6902;f_BMS3Bbin08;g_BMS3Bbin08;s_BMS3Bbin08 sp002897775	56.19	0.00
Nitrospirae bacterium CG02_land_8_20_14_3_00_41_53	GCA_002780895.1	2.01	159	40.63	p_Nitrospirota;c_Thermodesulfovibrionia;o_Thermodesulfovibrionales;f_SM23-35;g_0-14-3-00-41-53;s_0-14-3-00-41-53 sp002780895	78.36	1.75
Nitrospirae bacterium CG1_02_44_142	GCA_001871685.1	1.79	138	43.94	p_Nitrospirota;c_Thermodesulfovibrionia;o_Thermodesulfovibrionales;f_UBA1546;g_UBA1546;s_UBA1546 sp001871685	95.67	1.29
Nitrospirae bacterium DC0425bin1	GCA_002753685.1	4.02	107	49.04	p_Nitrospirota;c_Thermodesulfovibrionia;o_Thermodesulfovibrionales;f_Magnetobacteriaceae;g_Magnetobacterium;s_Magnetobacterium sp002753685	97.06	0.91
Nitrospirae bacterium DP16D_bin.35	GCA_004321915.1	3.63	145	48.51	p_Nitrospirota;c_Thermodesulfovibrionia;o_Thermodesulfovibrionales;f_UBA9935;g_MYbin3;s_MYbin3 sp004321915	53.73	0.05
Nitrospirae bacterium DP16D_bin.48	GCA_004321925.1	3.23	120	54.48	p_Nitrospirota;c_Thermodesulfovibrionia;o_Thermodesulfovibrionales;f_UBA6898;g_PALSA-1316;s_PALSA-1316 sp004321925	82.29	0.81
Nitrospirae bacterium FW300_bin.22	GCA_004298625.1	2.34	416	45.48	p_Nitrospirota;c_Thermodesulfovibrionia;o_Thermodesulfovibrionales;f_UBA1546;g_SCSY01;s_SCSY01 sp004298625	81.55	0.25
Nitrospirae bacterium FW300_bin.52	GCA_004298335.1	2.66	250	63.01	p_Nitrospirota;c_Nitrospiria;o_Nitrospirales;f_NS-4;g_SCTG01;s_SCTG01 sp004298335	97.42	1.29
Nitrospirae bacterium Glo_13	GCA_003354025.1	4.17	642	42.23	p_Nitrospirota;c_Thermodesulfovibrionia;o_UBA6902;f_UBA6902;g_Glo-13;s_Glo-13 sp003354025	96.57	2.94
Nitrospirae bacterium GW715_bin.13	GCA_004297235.1	4.17	613	57.81	p_Nitrospirota;c_Nitrospiria;o_SBBL01;f_SCUR01;g_Manganitrophus;s_Manganitrophus sp004297235	73.57	1.94
Nitrospirae bacterium GW928_bin.16	GCA_004296885.1	3.26	23	61.97	p_Nitrospirota;c_Nitrospiria;o_Nitrospirales;f_Nitrospiraceae;g_SYGV01;s_SYGV01 sp004296885	61.77	1.47
Nitrospirae bacterium GW928_bin.19	GCA_004296865.1	2.71	613	61.63	p_Nitrospirota;c_Nitrospiria;o_Nitrospirales;f_Nitrospiraceae;g_SCVQ01;s_SCVQ01 sp004296865	97.78	1.68
Nitrospirae bacterium GWB2_47_37	GCA_001803635.1	2.47	81	46.39	p_Nitrospirota;c_Thermodesulfovibrionia;o_Thermodesulfovibrionales;f_UBA9935;g_GWB2-47-37;s_GWB2-47-37 sp001803635	70.28	1.68
Nitrospirae bacterium GWC2_56_14	GCA_001803705.1	3.16	259	56.44	p_Nitrospirota;c_UBA9217;o_UBA9217;f_UBA9217;g_GWC2-56-14;s_GWC2-56-14 sp001803705	93.82	0.00
Nitrospirae bacterium GWC2_57_13	GCA_001805055.1	3.32	105	57.15	p_Nitrospirota;c_UBA9217;o_UBA9217;f_UBA9217;g_GWC2-57-13;s_GWC2-57-13 sp001805055	99.11	1.68
Nitrospirae bacterium GWF2_44_13	GCA_001805105.1	1.99	18	43.69	p_Nitrospirota;c_Thermodesulfovibrionia;o_Thermodesulfovibrionales;f_UBA1546;g_UBA1546;s_UBA1546 sp001805105	99.68	1.61
Nitrospirae bacterium HCH-1	GCA_001541255.1	3.59	152	45.37	p_Nitrospirota;c_Thermodesulfovibrionia;o_Thermodesulfovibrionales;f_Magnetobacteriaceae;g_HCH-1;s_HCH-1 sp001541255	91.07	2.68

Genome name	NCBI/IMG accession	Size, Mbp	Scaff, no.	GC, %	GTDB Taxonomy	Completeness, %	Contamination, %
Nitrospirae bacterium HCHbin1	GCA_002753435.1	3.69	94	45.24	p_Nitrospirota;c_Thermodesulfovibrionia;o_Thermodesulfovibrionales;f_Magnetobacteriaceae;g_HCH-1;s_HCH-1 sp001541255	95.66	0.00
Nitrospirae bacterium J017	GCA_003695915.1	3.71	135	54.77	p_Nitrospirota;c_Nitrospiria;o_Nitrospirales;f_UBA8639;g_J017;s_J017 sp003695915	78.06	5.22
Nitrospirae bacterium J031	GCA_003696975.1	2.77	273	53.77	p_Nitrospirota;c_Nitrospiria;o_Nitrospirales;f_UBA8639;g_J031;s_J031 sp003696975	58.65	0.00
Nitrospirae bacterium JdFR-81	GCA_002011735.1	2.05	95	48.33	p_Nitrospirota;c_Thermodesulfovibrionia;o_UBA6902;f_JdFR-81;g_JdFR-81;s_JdFR-81 sp002011735	61.47	1.68
Nitrospirae bacterium JdFR-85	GCA_002011745.1	2.33	47	41.54	p_Nitrospirota;c_Thermodesulfovibrionia;o_Thermodesulfovibrionales;f_JdFR-85;g_JdFR-85;s_JdFR-85 sp002011745	68.97	0.00
Nitrospirae bacterium JdFR-86	GCA_002011815.1	2.10	25	45.26	p_Nitrospirota;c_Thermodesulfovibrionia;o_Thermodesulfovibrionales;f_JdFR-86;g_JdFR-86;s_JdFR-86 sp002011815	63.44	1.08
Nitrospirae bacterium JdFR-88	GCA_002011795.1	1.86	22	62.9	p_Nitrospirota;c_Thermodesulfovibrionia;o_Thermodesulfovibrionales;f_JdFR-88;g_JdFR-88;s_JdFR-88 sp002011795	84.62	0.16
Nitrospirae bacterium MAG_10313_ntr_31	GCA_013349595.1	1.93	344	35.33	p_Nitrospirota;c_Thermodesulfovibrionia;o_Thermodesulfovibrionales;f_DUZI01;g_DUZI01;s_DUZI01 sp013349595	88.39	0.32
Nitrospirae bacterium MYbin2	GCA_002753455.1	3.07	89	48.79	p_Nitrospirota;c_Thermodesulfovibrionia;o_Thermodesulfovibrionales;f_Magnetobacteriaceae;g_Magnetobacterium;s_Magnetobacterium casensis	85.64	5.45
Nitrospirae bacterium MYbin3	GCA_002753335.1	2.93	66	44.36	p_Nitrospirota;c_Thermodesulfovibrionia;o_Thermodesulfovibrionales;f_UBA9935;g_MYbin3;s_MYbin3 sp002753335	81.86	1.68
Nitrospirae bacterium MYbin6	GCA_002753305.1	3.60	218	47.78	p_Nitrospirota;c_Thermodesulfovibrionia;o_Thermodesulfovibrionales;f_Magnetobacteriaceae;g_HCH-1;s_HCH-1 sp002753305	83.77	1.79
Nitrospirae bacterium MYbinv3	GCA_002753395.1	3.71	175	44.44	p_Nitrospirota;c_Thermodesulfovibrionia;o_Thermodesulfovibrionales;f_Magnetobacteriaceae;g_Magnetobacterium;s_Magnetobacterium sp002753395	59.65	0.00
Nitrospirae bacterium nDC0425bin1	GCA_015233985.1	4.04	178	49.09	p_Nitrospirota;c_Thermodesulfovibrionia;o_Thermodesulfovibrionales;f_Magnetobacteriaceae;g_Magnetobacterium;s_Magnetobacterium sp002753685	67.50	1.08
Nitrospirae bacterium nDJH13bin1	GCA_015233725.1	3.32	168	49.07	p_Nitrospirota;c_Thermodesulfovibrionia;o_Thermodesulfovibrionales;f_Magnetobacteriaceae;g_Magnetobacterium;s_	99.50	0.50
Nitrospirae bacterium nDJH13bin15	GCA_015233685.1	2.03	121	48.07	p_Nitrospirota;c_Thermodesulfovibrionia;o_Thermodesulfovibrionales;f_Magnetobacteriaceae;g_HCH-1;s_	94.12	2.10
Nitrospirae bacterium nDJH13bin21	GCA_015233655.1	3.29	262	47.98	p_Nitrospirota;c_Thermodesulfovibrionia;o_Thermodesulfovibrionales;f_UBA9935;g_GCA-2634385;s_	98.18	0.91
Nitrospirae bacterium nDJH13bin3	GCA_015233615.1	4.01	220	47.81	p_Nitrospirota;c_Thermodesulfovibrionia;o_Thermodesulfovibrionales;f_Magnetobacteriaceae;g_;s_	98.51	0.50
Nitrospirae bacterium nDJH14bin5	GCA_015233555.1	3.59	69	41.5	p_Nitrospirota;c_Thermodesulfovibrionia;o_Thermodesulfovibrionales;f_Magnetobacteriaceae;g_;s_	92.24	1.68
Nitrospirae bacterium nDJH14bin7	GCA_015233485.1	4.24	565	44.94	p_Nitrospirota;c_Thermodesulfovibrionia;o_Thermodesulfovibrionales;f_Magnetobacteriaceae;g_HCH-1;s_	96.47	3.36
Nitrospirae bacterium nDJH14bin9	GCA_015233465.1	3.13	75	35.21	p_Nitrospirota;c_Thermodesulfovibrionia;o_Thermodesulfovibrionales;f_;g_;s_	98.71	1.36
Nitrospirae bacterium nDJH15bin2	GCA_015233455.1	3.58	245	49.18	p_Nitrospirota;c_Thermodesulfovibrionia;o_Thermodesulfovibrionales;f_Magnetobacteriaceae;g_Magnetobacterium;s_	76.25	0.50
Nitrospirae bacterium nDJH15bin8	GCA_015233365.1	2.18	62	48.65	p_Nitrospirota;c_Thermodesulfovibrionia;o_Thermodesulfovibrionales;f_UBA9935;g_GCA-2634385;s_	96.00	0.00
Nitrospirae bacterium nDJH5bin4	GCA_015233265.1	2.54	344	47.41	p_Nitrospirota;c_Thermodesulfovibrionia;o_Thermodesulfovibrionales;f_Magnetobacteriaceae;g_HCH-1;s_	90.84	0.00
Nitrospirae bacterium nDJH6bin1	GCA_015233245.1	3.47	318	41.13	p_Nitrospirota;c_Thermodesulfovibrionia;o_Thermodesulfovibrionales;f_Magnetobacteriaceae;g_;s_	98.62	2.52
Nitrospirae bacterium nDJH8bin13	GCA_015233045.1	2.61	190	47.66	p_Nitrospirota;c_Thermodesulfovibrionia;o_Thermodesulfovibrionales;f_Magnetobacteriaceae;g_HCH-1;s_	93.38	0.75
Nitrospirae bacterium nDJH8bin6	GCA_015233015.1	3.78	149	46.6	p_Nitrospirota;c_Thermodesulfovibrionia;o_Thermodesulfovibrionales;f_Magnetobacteriaceae;g_HCH-1;s_	99.58	0.84
Nitrospirae bacterium nDJH8bin7	GCA_015232945.1	1.91	354	35.14	p_Nitrospirota;c_Thermodesulfovibrionia;o_Thermodesulfovibrionales;f_;g_;s_	88.80	0.56
Nitrospirae bacterium nDJH8bin8	GCA_015232995.1	4.19	111	41.49	p_Nitrospirota;c_Thermodesulfovibrionia;o_Thermodesulfovibrionales;f_Magnetobacteriaceae;g_;s_	51.04	0.00
Nitrospirae bacterium nHCHbin2	GCA_015232615.1	3.70	99	45.24	p_Nitrospirota;c_Thermodesulfovibrionia;o_Thermodesulfovibrionales;f_Magnetobacteriaceae;g_HCH-1;s_HCH-1 sp001541255	88.82	1.20
Nitrospirae bacterium nMYbin1	GCA_015232195.1	2.91	48	44.36	p_Nitrospirota;c_Thermodesulfovibrionia;o_Thermodesulfovibrionales;f_UBA9935;g_MYbin3;s_MYbin3 sp002753335	84.03	4.62
Nitrospirae bacterium nMYbin2	GCA_015232185.1	3.49	127	49	p_Nitrospirota;c_Thermodesulfovibrionia;o_Thermodesulfovibrionales;f_Magnetobacteriaceae;g_Magnetobacterium;s_Magnetobacterium casensis	52.67	0.42
Nitrospirae bacterium nMYbin3	GCA_015232165.1	1.92	278	49.89	p_Nitrospirota;c_Thermodesulfovibrionia;o_Thermodesulfovibrionales;f_Magnetobacteriaceae;g_Magnetobacterium;s_	80.97	0.65
Nitrospirae bacterium nMYbin4	GCA_015232145.1	3.93	213	44.04	p_Nitrospirota;c_Thermodesulfovibrionia;o_Thermodesulfovibrionales;f_Magnetobacteriaceae;g_Magnetobacterium;s_Magnetobacterium sp002753395	90.08	2.73
Nitrospirae bacterium nMYbin6	GCA_015232115.1	3.40	171	47.77	p_Nitrospirota;c_Thermodesulfovibrionia;o_Thermodesulfovibrionales;f_Magnetobacteriaceae;g_HCH-1;s_HCH-1 sp002753305	70.68	0.75
Nitrospirae bacterium NS_7	GCA_005877775.1	3.06	309	58.5	p_Nitrospirota;c_Nitrospiria;o_Nitrospirales;f_Nitrospiraceae;g_NS-7;s_NS-7 sp005877775	97.01	0.00
Nitrospirae bacterium NS_8	GCA_005877565.1	2.23	265	62.11	p_Nitrospirota;c_Nitrospiria;o_Nitrospirales;f_NS-4;g_NS-12;s_NS-12 sp005877565	93.01	1.08
Nitrospirae bacterium NS_9	GCA_005887945.1	3.54	366	56.73	p_Nitrospirota;c_Nitrospiria;o_Nitrospirales;f_Nitrospiraceae;g_Palsa-1315;s_Palsa-1315 sp005887945	68.82	0.18
Nitrospirae bacterium RBG_13_39_12	GCA_001805125.1	2.93	176	39.49	p_Nitrospirota;c_Thermodesulfovibrionia;o_Thermodesulfovibrionales;f_SM23-35;g_RBG-13-39-12;s_RBG-13-39-12 sp001805125	80.66	2.94
Nitrospirae bacterium RIFCSPLOWO2_02_FULL_62_14	GCA_001805245.1	2.66	76	61.66	p_Nitrospirota;c_Nitrospiria;o_Nitrospirales;f_Nitrospiraceae;g_2-02-FULL-62-14;s_2-02-FULL-62-14 sp001805245	100.00	0.91
Nitrospirae bacterium SZUA-452	GCA_003252075.1	2.86	146	52.15	p_Nitrospirota;c_Thermodesulfovibrionia;o_Thermodesulfovibrionales;f_UBA6898;g_UBA6898;s_UBA6898 sp003252075	73.59	2.21
Oligoflexales bacterium nHA4bin4	GCA_015232735.1	4.29	942	43.67	p_Bdellovibrionota;c_Oligoflexia;o_Oligoflexales;f_Oligoflexaceae;g_;s_	100.00	0.00
Oligoflexia bacterium nKLKbin12	GCA_015232355.1	5.63	96	32.16	p_Bdellovibrionota;c_Bacteriovoracia;o_Bacteriovoracales;f_;g_;s_	98.88	1.12
Oligoflexia bacterium nKLKbin5	GCA_015232255.1	4.59	212	41.45	p_Bdellovibrionota;c_Bacteriovoracia;o_Bacteriovoracales;f_;g_;s_	100.00	1.69
Oligoflexia bacterium nN3bin16	GCA_015232015.1	4.91	413	31.52	p_Bdellovibrionota;c_Bacteriovoracia;o_Bacteriovoracales;f_;g_;s_	100.00	1.14
Oligoflexia bacterium nN3bin31	GCA_015231955.1	5.61	114	40.9	p_Bdellovibrionota;c_Bacteriovoracia;o_Bacteriovoracales;f_;g_;s_	98.32	0.84
Oligoflexia bacterium nW3bin19	GCA_015231325.1	4.37	470	38.41	p_Bdellovibrionota;c_Bacteriovoracia;o_Bacteriovoracales;f_Bacteriovoracaceae;g_;s_	98.78	0.00
Omnitrophica bacterium GWA2_52_8	GCA_001804025.1	2.20	146	51.7	p_Omnitrophota;c_Omnitrophia;o_Omnitrophales;f_GWA2-52-8;g_GWA2-52-8;s_GWA2-52-8 sp001804025	85.56	0.45
Omnitrophica bacterium SCGC AG-290-C17	3300015153	1.71	171	48.60	p_Omnitrophota;c_Omnitrophia;o_Omnitrophales;f_GWA2-52-8;g_;s_	62.84	0.00
Omnitrophica WOR_2 bacterium GWA2_45_18	GCA_001804395.1	2.34	24	46.36	p_Omnitrophota;c_Koll11;o_UBA10015;f_Kpj58rc;g_UBA10174;s_UBA10174 sp003528115	62.54	1.08
Omnitrophica WOR_2 bacterium GWC2_45_7	GCA_001805405.1	1.34	98	45.06	p_Omnitrophota;c_Koll11;o_UBA10015;f_Kpj58rc;g_UBA10174;s_UBA10174 sp003528115	72.63	1.06
Pelobacter propionicus DSM 2379	GCF_000015045.1	4.24	3	58.48	p_Desulfobacterota_F;c_Desulfuromonadia;o_Geobacterales;f_Pseudopelobacteraceae;g_Pseudopelobacter;s_Pseudopelobacter propionicus	94.03	2.73
Pelobacter seleniigenes DSM 18267	GCA_000711225.1	5.09	6	54.19	p_Desulfobacterota_F;c_Desulfuromonadia;o_Desulfuromonadales;f_Geopsychrobacteraceae;g_Seleniibacterium;s_Seleniibacterium seleniigenes	96.48	3.18
Pelobacter sp. UBA5610	GCA_002424645.1	2.70	168	53.45	p_Desulfobacterota_F;c_Desulfuromonadia;o_Desulfuromonadales;f_Syntrophotaleaceae;g_Syntrophotalea;s_Syntrophotalea sp002424645	63.39	1.22
Pelobacter sp. UBA6812	GCA_002452265.1	3.02	90	52.83	p_Desulfobacterota_F;c_Desulfuromonadia;o_Desulfuromonadales;f_Syntrophotaleaceae;g_Syntrophotalea;s_Syntrophotalea sp002452265	91.27	2.38
Phaeospirillum molischianum DSM 120	GCF_000294655.1	3.81	61	61.51	p_Proteobacteria;c_Alphaproteobacteria;o_Rhodospirillales;f_Magnetospirillaceae;g_Phaeospirillum;s_Phaeospirillum molischianum	58.23	0.81
Phycisphaera mikurensis NBRC 102666	GCA_000284115.1	3.88	2	73.22	p_Planctomycetota;c_Phycisphaerae;o_Phycisphaerales;f_Phycisphaeraceae;g_Phycisphaera;s_Phycisphaera mikurensis	95.85	0.91
Phycisphaerae bacterium ST-NAGAB-D1	GCA_002007645.1	4.25	1	51.98	p_Planctomycetota;c_Phycisphaerae;o_Sedimentisphaerales;f_Anaerohalophaeraceae;g_Anaerohalosphaera;s_Anaerohalosphaera lusitana	96.00	1.33
Phycisphaerales bacterium UBA1845	GCA_002338715.1	5.25	148	57	p_Planctomycetota;c_Phycisphaerae;o_UBA1845;f_UBA1845;g_UBA1845;s_UBA1845 sp002338715	85.99	2.25
Pirellula staleyi DSM 6068	GCA_000025185.1	6.20	1	57.46	p_Planctomycetota;c_Planctomycetes;o_Pirellulales;f_Pirellulaceae;g_Pirellula;s_Pirellula staleyi	54.51	0.00

Genome name	NCBI/IMG accession	Size, Mbp	Scaff, no.	GC, %	GTDB Taxonomy	Completeness, %	Contamination, %
Planctomycetes bacterium MAG_11118_pl_115	GCA_013349615.1	3.77	157	48.98	p_Planctomycetota;c_Phycisphaerae;o_Sedimentisphaerales;f_SG8-4;g_CS2-K091;s_CS2-K091 sp013349615	56.21	0.00
Planctomycetes bacterium MAG_17991_pl_60	GCA_013349525.1	1.31	145	49.53	p_Planctomycetota;c_Phycisphaerae;o_Sedimentisphaerales;f_Anaerohalophaeraceae;g_QNBT01;s_QNBT01 sp013349435	77.94	1.82
Planctomycetes bacterium MAG_18080_pl_157	GCA_013349435.1	3.14	139	48.44	p_Planctomycetota;c_Phycisphaerae;o_Sedimentisphaerales;f_Anaerohalophaeraceae;g_QNBT01;s_QNBT01 sp013349435	73.86	1.33
Planctomycetes bacterium nTSbin13	GCA_015231495.1	3.00	642	56.47	p_Planctomycetota;c_UBA11346;o_;f_;g_;s_	88.29	2.15
Planctomycetes bacterium nwagbin3	GCA_015231345.1	4.14	588	41.73	p_Planctomycetota;c_UBA11346;o_;f_;g_;s_	75.50	1.29
Planctomycetes bacterium SCGC JGI090-P21	2264265205	1.23	242	49.20	p_Planctomycetota;c_Phycisphaerae;o_UBA1845;f_PWPN01;g_;s_	38.87	2.19
Planctomycetes bacterium SM23_25	GCA_001303605.1	3.77	418	66.68	p_Planctomycetota;c_Phycisphaerae;o_FEN-1346;f_FEN-1346;g_;s_	60.62	0.32
Pseudomonas aeruginosa JCM 5962	GCA_000615485.1	6.07	1198	66.28	p_Proteobacteria;c_Gammaproteobacteria;o_Pseudomonadales;f_Pseudomonadaceae;g_Pseudomonas;s_Pseudomonas aeruginosa	99.04	0.00
Rhodospirillaceae bacterium MAG_01419_mvb_30	3300001419	2.81	477	55.72	p_Proteobacteria;c_Alphaproteobacteria;o_Rhodospirillales;f_Magnetovibrionaceae;g_Magnetovibrio;s_	94.58	4.10
Rhodospirillaceae bacterium MAG_04806_tlms_2	GCA_013349735.1	2.09	309	57.51	p_Proteobacteria;c_Alphaproteobacteria;o_Rhodospirillales;f_Magnetospirillaceae;g_Telmatospirillum;s_Telmatospirillum sp013349735	94.56	1.08
Rhodospirillaceae bacterium MAG_05422_2-02_14	GCA_013349695.1	2.28	255	61.09	p_Proteobacteria;c_Alphaproteobacteria;o_Rhodospirillales;f_2-02-FULL-58-16;g_GCA-2686765;s_GCA-2686765 sp013349695	71.32	1.79
Rhodospirillaceae bacterium MAG_05596_2-02_51	GCA_013349685.1	1.83	329	61.19	p_Proteobacteria;c_Alphaproteobacteria;o_Rhodospirillales;f_2-02-FULL-58-16;g_GCA-2686765;s_GCA-2686765 sp013349695	80.77	2.10
Rhodospirillaceae bacterium MAG_06104_tlms_034	GCA_013349665.1	3.19	353	64.25	p_Proteobacteria;c_Alphaproteobacteria;o_Rhodospirillales;f_Magnetospirillaceae;g_Telmatospirillum;s_Telmatospirillum sp013349665	99.41	0.59
Rhodospirillaceae bacterium MAG_22225_2-02_112	GCA_013349335.1	2.55	147	61.01	p_Proteobacteria;c_Alphaproteobacteria;o_Rhodospirillales;f_2-02-FULL-58-16;g_GCA-2686765;s_GCA-2686765 sp013349695	54.48	0.00
Rhodospirillum rubrum ATCC 11170	GCF_000013085.1	4.41	2	65.38	p_Proteobacteria;c_Alphaproteobacteria;o_Rhodospirillales;f_Rhodospirillaceae;g_Rhodospirillum;s_Rhodospirillum rubrum	98.06	1.29
SAR324 cluster bacterium nKLKbin6	GCA_015232315.1	5.25	88	45.21	p_SAR324;c_SAR324;o_SAR324;f_;g_;s_	100.00	0.65
SAR324 cluster bacterium nPCRbin10	GCA_015231885.1	5.13	241	42.00	p_SAR324;c_SAR324;o_SAR324;f_GCA-2753255;g_GCA-2753255;s_GCA-2753255 sp002753255	95.97	2.15
SAR324 cluster bacterium nPCRbin7	GCA_015231825.1	5.80	81	44.77	p_SAR324;c_SAR324;o_SAR324;f_GCA-2753255;g_;s_	96.82	3.92
SAR324 cluster bacterium nTSbin10	GCA_015231535.1	5.76	344	44.87	p_SAR324;c_SAR324;o_SAR324;f_;g_;s_	100.00	0.00
Sebaldella termitidis ATCC 33386	GCA_000024405.1	4.49	3	33.38	p_Fusobacteriota;c_Fusobacteriia;o_Fusobacteriales;f_Leptotrichiaceae;g_Sebaldella;s_Sebaldella termitidis	64.20	0.00
Smithella sp. D17	GCA_000753945.1	1.64	271	43.19	p_Desulfobacterota;c_Syntrophia;o_Syntrophales;f_Smithellaceae;g_Smithella;s_Smithella sp000753945	97.42	3.21
Smithella sp. M82	GCA_001683985.1	2.75	236	42.83	p_Desulfobacterota;c_Syntrophia;o_Syntrophales;f_Smithellaceae;g_Smithella;s_Smithella sp001683985	98.71	2.72
Smithella sp. PtaU1.Bin162	GCA_002068015.1	3.60	191	45.33	p_Desulfobacterota;c_Syntrophia;o_Syntrophales;f_Smithellaceae;g_Fen-1166;s_Fen-1166 sp002068015	96.59	0.91
Smithella sp. SCADC	GCA_000747625.2	3.19	244	43.97	p_Desulfobacterota;c_Syntrophia;o_Syntrophales;f_Smithellaceae;g_Smithella;s_Smithella sp000747625	96.97	0.00
Smithella sp. SDB_sulfate1 SDB_sulfate2	GCA_007244375.1	3.10	58	41.44	p_Desulfobacterota;c_Syntrophia;o_Syntrophales;f_Smithellaceae;g_Smithella;s_Smithella sp001412345	96.77	1.94
Smithella sp. UBA2180	GCA_002327415.1	1.75	189	42.54	p_Desulfobacterota;c_Syntrophia;o_Syntrophales;f_Smithellaceae;g_Smithella;s_Smithella sp002327415	96.45	0.97
Streptobacillus moniliformis DSM 12112	GCA_000024565.1	1.67	2	26.28	p_Fusobacteriota;c_Fusobacteriia;o_Fusobacteriales;f_Leptotrichiaceae;g_Streptobacillus;s_Streptobacillus moniliformis	60.01	1.16
Sulfurivirga caldicuralii DSM 17737	GCF_900141795.1	1.67	7	59.19	p_Proteobacteria;c_Gammaproteobacteria;o_Thiomicrospirales;f_Thiomicrospiraceae;g_Sulfurivirga;s_Sulfurivirga caldicuralii	99.50	0.75
Syntrophaceae bacterium bog_1110	GCA_003139775.1	3.42	206	43.25	p_Desulfobacterota;c_Syntrophia;o_Syntrophales;f_Smithellaceae;g_Smithella;s_Smithella sp003139775	88.28	1.29
Syntrophaceae bacterium bog_1155	GCA_003165115.1	3.91	521	58.82	p_Desulfobacterota_G;c_Syntrophorhabdia;o_Syntrophorhabdales;f_WCHB1-27;g_BOG-1155;s_BOG-1155 sp003165115	91.76	10.88
Syntrophaceae bacterium CG2_30_49_12	GCA_001873675.1	2.03	223	48.47	p_Desulfobacterota;c_Syntrophia;o_Syntrophales;f_CG2-30-49-12;g_CG2-30-49-12;s_CG2-30-49-12 sp001873675	84.26	2.15
Syntrophaceae bacterium CG2_30_58_14	GCA_001873745.1	3.04	204	57.77	p_Desulfobacterota;c_Syntrophia;o_Syntrophales;f_UBA5619;g_UBA5619;s_UBA5619 sp001873745	95.82	6.47
Syntrophaceae bacterium fen_1087	GCA_003161855.1	1.81	197	50.66	p_Desulfobacterota;c_Syntrophia;o_Syntrophales;f_Fen-1087;g_Fen-1087;s_Fen-1087 sp003161855	95.81	0.65
Syntrophaceae bacterium fen_1141	GCA_003154025.1	5.73	574	56.46	p_Desulfobacterota;c_SM23-61;o_SM23-61;f_SM23-61;g_SM23-61;s_SM23-61 sp003154025	96.97	1.82
Syntrophaceae bacterium fen_1145	GCA_003153775.1	3.13	98	49.99	p_Desulfobacterota;c_Syntrophia;o_Syntrophales;f_UBA2185;g_Fen-1135;s_Fen-1135 sp003153775	91.82	0.91
Syntrophaceae bacterium fen_1160	GCA_003142835.1	2.49	188	44.35	p_Desulfobacterota;c_Syntrophia;o_Syntrophales;f_Smithellaceae;g_FEN-1160;s_FEN-1160 sp003142835	87.74	3.44
Syntrophaceae bacterium fen_1164	GCA_003142635.1	3.47	260	49.56	p_Desulfobacterota;c_Syntrophia;o_Syntrophales;f_UBA2185;g_Fen-1135;s_Fen-1135 sp003142635	91.82	0.00
Syntrophaceae bacterium fen_1165	GCA_003141615.1	3.54	218	43.67	p_Desulfobacterota;c_Syntrophia;o_Syntrophales;f_Smithellaceae;g_Smithella;s_Smithella sp003141615	97.42	3.23
Syntrophaceae bacterium fen_1166	GCA_003142535.1	2.93	70	43.42	p_Desulfobacterota;c_Syntrophia;o_Syntrophales;f_Smithellaceae;g_Fen-1166;s_Fen-1166 sp003142535	99.58	0.84
Syntrophaceae bacterium fen_1168	GCA_003142295.1	3.28	250	48	p_Desulfobacterota;c_Syntrophia;o_Syntrophales;f_UBA2185;g_Fen-1135;s_Fen-1135 sp003142295	96.36	3.64
Syntrophaceae bacterium fen_1170	GCA_003141195.1	3.27	108	57.46	p_Desulfobacterota;c_Syntrophia;o_Syntrophales;f_UBA5619;g_UBA5619;s_UBA5619 sp003141195	65.48	2.26
Syntrophaceae bacterium PtaB.Bin038	GCA_002067855.1	2.55	433	64.54	p_Desulfobacterota;c_Syntrophia;o_Syntrophales;f_UBA4778;g_UBA2192;s_UBA2192 sp002067855	93.55	1.29
Syntrophaceae bacterium UBA10514	GCA_003508565.1	2.25	298	45.94	p_Desulfobacterota;c_Syntrophia;o_Syntrophales;f_UBA2185;g_UBA2185;s_UBA2185 sp003508565	94.35	3.25
Syntrophaceae bacterium UBA1062	GCA_002316295.1	3.13	295	58.13	p_Desulfobacterota;c_Desulfomonilia;o_UBA1062;f_UBA1062;g_UBA1062;s_UBA1062 sp002316295	80.27	4.03
Syntrophaceae bacterium UBA1411	GCA_002304995.1	3.42	148	57.94	p_Desulfobacterota;c_Syntrophia;o_Syntrophales;f_Smithellaceae;g_UBA8904;s_UBA8904 sp002304995	93.44	7.74
Syntrophaceae bacterium UBA2188	GCA_002327985.1	3.98	186	45.72	p_Desulfobacterota;c_BSN033;o_BSN033;f_UBA1163;g_UBA1163;s_UBA1163 sp002327985	100.00	0.00
Syntrophaceae bacterium UBA2192	GCA_002327315.1	3.66	227	59.42	p_Desulfobacterota;c_Syntrophia;o_Syntrophales;f_UBA4778;g_UBA2192;s_UBA2192 sp002327315	92.74	2.17
Syntrophaceae bacterium UBA2210	GCA_002327865.1	2.21	60	48.11	p_Desulfobacterota;c_Syntrophia;o_Syntrophales;f_UBA2210;g_UBA2210;s_UBA2210 sp002327865	96.45	1.33
Syntrophaceae bacterium UBA2251	GCA_002347815.1	2.73	85	62.98	p_Desulfobacterota;c_Syntrophia;o_Syntrophales;f_UBA2251;g_UBA2251;s_UBA2251 sp002347815	93.91	5.22
Syntrophaceae bacterium UBA2280	GCA_002347685.1	3.87	71	51.55	p_Desulfobacterota;c_Syntrophia;o_Syntrophales;f_Smithellaceae;g_UBA8904;s_UBA8904 sp002347685	99.35	0.65
Syntrophaceae bacterium UBA3084	GCA_002367335.1	2.10	237	42.36	p_Desulfobacterota;c_Syntrophia;o_Syntrophales;f_UBA6807;g_UBA3084;s_UBA3084 sp002367335	91.17	8.06
Syntrophaceae bacterium UBA4054	GCA_002383495.1	2.66	67	53.14	p_Desulfobacterota;c_Syntrophia;o_Syntrophales;f_UBA4778;g_UBA4054;s_UBA4054 sp002383495	98.06	1.29
Syntrophaceae bacterium UBA4059	GCA_002382045.1	2.95	229	49.21	p_Desulfobacterota;c_Syntrophia;o_Syntrophales;f_Smithellaceae;g_UBA8904;s_UBA8904 sp002382045	95.38	1.94
Syntrophaceae bacterium UBA4767	GCA_002403385.1	1.83	82	45.92	p_Desulfobacterota;c_Syntrophia;o_Syntrophales;f_UBA4767;g_UBA4767;s_UBA4767 sp002403385	92.42	4.30
Syntrophaceae bacterium UBA4778	GCA_002403175.1	3.44	342	46.45	p_Desulfobacterota;c_Syntrophia;o_Syntrophales;f_UBA4778;g_UBA4778;s_UBA4778 sp002403175	88.50	5.16
Syntrophaceae bacterium UBA4810	GCA_002402545.1	2.31	109	44.61	p_Desulfobacterota;c_Syntrophia;o_Syntrophales;f_Smithellaceae;g_UBA4810;s_UBA4810 sp002402545	94.03	1.51
Syntrophaceae bacterium UBA5619	GCA_002424545.1	2.60	135	58.82	p_Desulfobacterota;c_Syntrophia;o_Syntrophales;f_UBA5619;g_UBA5619;s_UBA5619 sp002424545	96.22	0.84
Syntrophaceae bacterium UBA5744	GCA_002419215.1	3.47	154	60.36	p_Desulfobacterota;c_Syntrophia;o_Syntrophales;f_UBA4778;g_UBA2192;s_UBA2192 sp002419215	86.03	1.61
Syntrophaceae bacterium UBA5761	GCA_002418885.1	2.03	89	55.6	p_Desulfobacterota;c_Syntrophia;o_Syntrophales;f_UBA4778;g_UBA2207;s_UBA2207 sp002418885	87.13	0.65

Genome name	NCBI/IMG accession	Size, Mbp	Scaff, no.	GC, %	GTDB Taxonomy	Completeness, %	Contamination, %
Syntrophaceae bacterium UBA5764	GCA_002418825.1	2.12	98	45.52	p_Desulfobacterota;c_Syntrophia;o_Syntrophales;f_Smithellaceae;g_UBA4810;s_UBA4810 sp002418825	98.81	0.00
Syntrophaceae bacterium UBA6078	GCA_002428735.1	3.00	82	54.29	p_Desulfobacterota;c_Syntrophia;o_Syntrophales;f_UBA2210;g_UBA6078;s_UBA6078 sp002428735	95.48	3.44
Syntrophaceae bacterium UBA6109	GCA_002428445.1	4.19	98	58.99	p_Desulfobacterota;c_Syntrophia;o_Syntrophales;f_UBA6109;g_UBA6109;s_UBA6109 sp002428445	95.81	3.15
Syntrophaceae bacterium UBA6112	GCA_002423465.1	3.18	87	56.34	p_Desulfobacterota;c_Syntrophia;o_Syntrophales;f_UBA5619;g_UBA5619;s_UBA5619 sp002423465	91.72	0.91
Syntrophaceae bacterium UBA6252	GCA_002441265.1	3.31	70	60.78	p_Desulfobacterota;c_Syntrophia;o_Syntrophales;f_UBA6807;g_UBA6807;s_UBA6807 sp002441265	80.16	2.41
Syntrophaceae bacterium UBA6255	GCA_002441205.1	3.19	304	43.49	p_Desulfobacterota;c_Syntrophia;o_Syntrophales;f_Smithellaceae;g_Smithella;s_Smithella sp002441205	96.13	1.94
Syntrophaceae bacterium UBA7520	GCA_002478265.1	2.34	40	47.73	p_Desulfobacterota;c_Syntrophia;o_Syntrophales;f_Smithellaceae;g_UBA4810;s_UBA4810 sp002478265	98.13	1.64
Syntrophaceae bacterium UBA7544	GCA_002479215.1	2.98	305	43.29	p_Desulfobacterota;c_Syntrophia;o_Syntrophales;f_Smithellaceae;g_UBA4810;s_UBA4810 sp002479215	84.52	1.81
Syntrophaceae bacterium UBA8930	GCA_003506745.1	1.81	308	51.47	p_Desulfobacterota;c_Syntrophia;o_Syntrophales;f_Smithellaceae;g_UBA8904;s_UBA8904 sp003506745	93.07	2.73
Syntrophobacter fumaroxidans MPOB	GCF_000014965.1	4.99	1	59.95	p_Desulfobacterota;c_Syntrophobacteria;o_Syntrophobacterales;f_Syntrophobacteraceae;g_Syntrophobacter;s_Syntrophobacter fumaroxidans	85.47	0.97
Syntrophobacter sp. DG_60	GCA_001304365.1	1.36	128	39.12	p_Desulfobacterota;c_Desulfofervidia;o_Desulfofervidales;f_DG-60;g_DG-60;s_DG-60 sp001304365	93.55	2.96
Syntrophobacter sp. SbD1	GCA_900290365.1	4.67	182	52.45	p_Desulfobacterota;c_Syntrophobacteria;o_Syntrophobacterales;f_Syntrophobacteraceae;g_SbD1;s_SbD1 sp900290365	92.06	5.06
Syntrophobacter sp. SbD2	GCA_900290385.1	1.81	216	53.27	p_Desulfobacterota;c_Syntrophobacteria;o_Syntrophobacterales;f_Syntrophobacteraceae;g_SbD1;s_SbD1 sp900290385	77.51	2.26
Syntrophobacteraceae bacterium bog_1154	GCA_003165235.1	7.07	424	52.86	p_Desulfobacterota;c_Syntrophobacteria;o_Syntrophobacterales;f_Syntrophobacteraceae;g_SbD1;s_SbD1 sp003165235	65.50	0.65
Syntrophobacteraceae bacterium CSSed162cmB_493R1	GCA_007125915.1	2.35	269	54.87	p_Desulfobacterota;c_Syntrophobacteria;o_Syntrophobacterales;f_Syntrophobacteraceae;g_SLCH01;s_SLCH01 sp007125915	82.46	1.75
Syntrophobacteraceae bacterium T3Sed10_239	GCA_003566995.1	3.04	159	55.34	p_Desulfobacterota;c_Syntrophobacteria;o_Syntrophobacterales;f_Syntrophobacteraceae;g_SLCH01;s_SLCH01 sp003566995	76.12	0.00
Syntrophobacterales bacterium Delta_01	GCA_001603845.1	4.29	525	59.72	p_Desulfobacterota;c_Syntrophobacteria;o_Syntrophobacterales;f_Syntrophobacteraceae;g_Delta-01;s_Delta-01 sp001603845	90.74	0.77
Syntrophobacterales bacterium GWC2_56_13	GCA_001830835.1	2.60	119	56.37	p_Desulfobacterota;c_Syntrophia;o_Syntrophales;f_UBA5619;g_UBA5619;s_UBA5619 sp001830835	77.98	3.36
Syntrophorhabdaceae bacterium PtaU1.Bin034	GCA_002067405.1	5.02	407	53.82	p_Desulfobacterota_G;c_Syntrophorhabdia;o_Syntrophorhabdales;f_WCHB1-27;g_BOG-1155;s_BOG-1155 sp002067405	75.74	0.00
Syntrophorhabdus sp. PtaB.Bin047	GCA_002067235.1	3.64	269	57.63	p_Desulfobacterota_G;c_Syntrophorhabdia;o_Syntrophorhabdales;f_Syntrophorhabdaceae;g_Delta-02;s_Delta-02 sp002067235	99.19	1.45
Syntrophus aciditrophicus SB	GCF_000013405.1	3.18	1	51.46	p_Desulfobacterota;c_Syntrophia;o_Syntrophales;f_Syntrophaceae;g_Syntrophus;s_Syntrophus aciditrophicus	92.09	2.73
Syntrophus gentianae DSM 8423	GCA_900109885.1	3.72	71	52.59	p_Desulfobacterota;c_Syntrophia;o_Syntrophales;f_Syntrophaceae;g_Syntrophus;s_Syntrophus gentianae	93.98	4.09
Syntrophus sp. (in: Bacteria) UBA8958	GCA_003451635.1	2.79	449	52.27	p_Desulfobacterota;c_Syntrophia;o_Syntrophales;f_UBA8958;g_UBA8958;s_UBA8958 sp003451635	89.09	2.73
Syntrophus sp. GWC2_56_31	GCA_001829875.1	2.06	136	55.77	p_Desulfobacterota;c_Syntrophia;o_Syntrophales;f_UBA5619;g_UBA5619;s_UBA5619 sp001829875	82.20	0.22
Syntrophus sp. PtaB.Bin001	GCA_002067815.1	2.84	582	48.91	p_Desulfobacterota;c_Syntrophia;o_Syntrophales;f_Syntrophaceae;g_Syntrophus;s_Syntrophus sp002067815	89.30	2.67
Syntrophus sp. PtaU1.Bin005	GCA_002067745.1	2.90	165	55.11	p_Desulfobacterota;c_Syntrophia;o_Syntrophales;f_Syntrophaceae;g_Syntrophus;s_Syntrophus sp002067745	94.03	2.73
Telmatospirillum siberiense 26-4b1	GCF_002845745.1	6.20	81	62.33	p_Proteobacteria;c_Alphaproteobacteria;o_Rhodospirillales;f_Magnetospirillaceae;g_Telmatospirillum;s_Telmatospirillum siberiense	89.28	2.09
Terasakiella pusilla DSM 6293	GCF_000688235.1	4.05	85	50.02	p_Proteobacteria;c_Alphaproteobacteria;o_Rhodospirillales;f_Terasakiellaceae;g_Terasakiella;s_Terasakiella pusilla	99.35	0.00
Thalassospira mesophila JCM 18969	GCF_002115755.1	4.93	94	54.25	p_Proteobacteria;c_Alphaproteobacteria;o_Rhodospirillales;f_Thalassospiraceae;g_Thalassospira;s_Thalassospira mesophila	99.00	0.00
Thermodesulfatator autotrophicus S606	GCF_001642325.1	2.27	129	43.08	p_Desulfobacterota;c_Thermodesulfobacteria;o_Thermodesulfobacteriales;f_Thermodesulfatatoraceae;g_Thermodesulfatator;s_autotrophicus	74.37	1.11
Thermodesulfatator indicus DSM 15286	GCF_000217795.1	2.32	1	42.43	p_Desulfobacterota;c_Thermodesulfobacteria;o_Thermodesulfobacteriales;f_Thermodesulfatatoraceae;g_Thermodesulfatator;s_Thermodesulfatator indicus	96.42	2.26
Thermodesulfobacteriaceae bacterium UBA6232	GCA_002441555.1	1.66	46	37.32	p_Desulfobacterota;c_Thermodesulfobacteria;o_Thermodesulfobacteriales;f_Thermodesulfobacteriaceae;g_Caldimicrobium;s_Caldimicrobium sp002441555	95.84	1.29
Thermodesulfobacterium commune DSM 2178	GCF_000734015.1	1.76	1	36.98	p_Desulfobacterota;c_Thermodesulfobacteria;o_Thermodesulfobacteriales;f_Thermodesulfobacteriaceae;g_Thermodesulfobacterium;s_commune	70.48	0.00
Thermodesulfobacterium hveragerdense DSM 12571	GCF_000423845.1	1.73	42	37.1	p_Desulfobacterota;c_Thermodesulfobacteria;o_Thermodesulfobacteriales;f_Thermodesulfobacteriaceae;g_Thermodesulfobacterium;s_hveragerdense	78.55	2.58
Thermodesulfobacterium thermophilum DSM 1276	GCF_000421605.1	1.79	46	37.07	p_Desulfobacterota;c_Thermodesulfobacteria;o_Thermodesulfobacteriales;f_Thermodesulfobacteriaceae;g_Thermodesulfobacterium;s_thermophilum	90.80	4.09
Thermodesulfovibrio aggregans TGE-P1	GCF_001514535.1	2.00	3	34.89	p_Nitrospirota;c_Thermodesulfovibrionia;o_Thermodesulfovibrionales;f_Thermodesulfovibrionaceae;g_Thermodesulfovibrio;s_aggregans	94.53	0.00
Thermodesulfovibrio hydrogeniphilus DSM_18151	2574179746	2.12	104	35.81	p_Nitrospirota;c_Thermodesulfovibrionia;o_Thermodesulfovibrionales;f_Thermodesulfovibrionaceae;g_;s_	99.03	0.00
Thermodesulfovibrio sp. N1	GCF_001707915.1	1.92	116	32.99	p_Nitrospirota;c_Thermodesulfovibrionia;o_Thermodesulfovibrionales;f_Thermodesulfovibrionaceae;g_Thermodesulfovibrio;s_sp001707915	90.62	0.89
Thermodesulfovibrio thiophilus DSM 17215	GCF_000423865.1	1.87	18	34.43	p_Nitrospirota;c_Thermodesulfovibrionia;o_Thermodesulfovibrionales;f_Thermodesulfovibrionaceae;g_Thermodesulfovibrio;s_thiophilus	99.16	3.64
Thermodesulfovibrio yellowstonii DSM 11347	GCF_000020985.1	2.00	1	34.13	p_Nitrospirota;c_Thermodesulfovibrionia;o_Thermodesulfovibrionales;f_Thermodesulfovibrionaceae;g_Thermodesulfovibrio;s_yellowstonii	85.14	1.68
Thiomicrospira pelophila DSM 1534	GCF_000711195.1	2.11	1	44.46	p_Proteobacteria;c_Gammaproteobacteria;o_Thiomicrospirales;f_Thiomicrospiraceae;g_Thiomicrospira;s_Thiomicrospira pelophila	94.14	1.82
Unclassified Nitrospina Bin 25	2651870060	4.16	431	37.69	p_Nitrospinota;c_Nitrospinia;o_Nitrospinales;f_;g_;s_	92.31	4.27
uncultured Desulfobacteraceae bacterium CR-1	GCA_900659855.1	3.25	52	54.03	p_Desulfobacterota;c_Desulfobacteria;o_Desulfobacterales;f_CR-1;g_CR-1;s_CR-1 sp900659855	87.88	0.65
uncultured Desulfobacterium sp. TRIP AH-1	GCA_900258555.1	5.43	28	47.01	p_Desulfobacterota;c_Desulfobacteria;o_Desulfatiglandales;f_Desulfatiglandaceae;g_UBA5623;s_UBA5623 sp900258555	83.55	2.26
uncultured Desulfovibrio sp. RUG514	GCA_900319575.1	2.14	253	59.19	p_Desulfobacterota;c_Desulfovibrionia;o_Desulfovibrionales;f_Desulfovibrionaceae;g_Desulfovibrio;s_Desulfovibrio sp900319575	91.31	0.91
uncultured Desulfovibrio sp. UMGS1330	GCA_900550745.1	2.38	425	61.53	p_Desulfobacterota;c_Desulfovibrionia;o_Desulfovibrionales;f_Desulfovibrionaceae;g_Bilophila;s_Bilophila sp900550745	88.39	0.65
uncultured Desulfovibrio sp. UMGS1580	GCA_900553065.1	1.92	70	58.43	p_Desulfobacterota;c_Desulfovibrionia;o_Desulfovibrionales;f_Desulfovibrionaceae;g_Mailhella;s_Mailhella sp900553065	96.13	0.84
uncultured Desulfovibrio sp. UMGS1890	GCA_900555975.1	1.51	319	59.28	p_Desulfobacterota;c_Desulfovibrionia;o_Desulfovibrionales;f_Desulfovibrionaceae;g_Mailhella;s_Mailhella sp900555975	77.14	0.96
uncultured Desulfovibrio sp. UMGS1966	GCA_900556755.1	1.38	32	62.94	p_Desulfobacterota;c_Desulfovibrionia;o_Desulfovibrionales;f_Desulfovibrionaceae;g_Desulfovibrio;s_Desulfovibrio sp900556755	64.52	0.00
uncultured Desulfovibrio sp. UMGS250	GCA_900540515.1	3.43	68	61.58	p_Desulfobacterota;c_Desulfovibrionia;o_Desulfovibrionales;f_Desulfovibrionaceae;g_Desulfovibrio;s_Desulfovibrio sp900540515	95.70	5.47
uncultured Desulfovibrio sp. UMGS847	GCA_900546145.1	2.46	72	58.52	p_Desulfobacterota;c_Desulfovibrionia;o_Desulfovibrionales;f_Desulfovibrionaceae;g_Desulfovibrio;s_Desulfovibrio sp900546145	73.21	0.00
Uncultured microorganism SbSrfc.SA12.01.D19	3300022116	2.50	175	52.60	p_Desulfobacterota;c_Desulfobulbia;o_Desulfobulbales;f_BM004;g_;s_	49.13	0.00
uncultured Nitrosospira sp. NOB2.fa.gz	GCA_900696505.1	3.25	413	58.19	p_Nitrospirota;c_Nitrospiria;o_Nitrospirales;f_Nitrospiraceae;g_Nitrospira_A;s_Nitrospira_A sp900696505	98.94	1.26
Zetaproteobacteria bacterium nPCbin1	GCA_015231855.1	2.02	68	47.46	p_Proteobacteria;c_Zetaproteobacteria;o_Mariprofundales;f_Mariprofundaceae;g_GCA-2753275;s_GCA-2753275 sp002753275	99.35	0.97
Zetaproteobacteria bacterium PCbin4	GCA_002753275.1	1.86	55	47.58	p_Proteobacteria;c_Zetaproteobacteria;o_Mariprofundales;f_Mariprofundaceae;g_GCA-2753275;s_GCA-2753275 sp002753275	98.81	0.00
Zetaproteobacteria bacterium SZUA-181	GCA_003228735.1	1.88	100	55.29	p_Proteobacteria;c_Zetaproteobacteria;o_Mariprofundales;f_Mariprofundaceae;g_SZUA-181;s_SZUA-181 sp003228735	99.35	0.00

Supplementary table S2. Results of reconciliations for protein trees and concatenated protein tree obtained by Notung and Ranger-DTL tools. LCMA – last common magnetotactic ancestor, LCDA – last common *Dissulfurispiraceae* ancestor, HGT – horizontal gene transfer.

Analysis	*Magnetobacteriaceae* group	*Dissulfurispiraceae* group	*man*-containing *Thermodesulfobacteriota* group
Man1 Ranger-DTL	Vertical inheritance from LCMA	HGT from *Magnetobacteriaceae* group	-
Man1 Notung	Vertical inheritance from LCMA	HGT from *Magnetobacteriaceae* group	-
Man2 Ranger-DTL	Vertical inheritance from LCMA	HGT from *Magnetobacteriaceae* group	HGT from *Magnetobacteriaceae* group
Man2 Notung	Vertical inheritance from LCMA	HGT from *Magnetobacteriaceae* group	Vertical inheritance from LCMA
Man3 Ranger-DTL	A reconciliation was not done due to the sequences shortness, which makes it difficult to avoid misinterpreting the results		
Man3 Notung			
Man4 Ranger-DTL	Vertical inheritance from LCMA	HGT from *Magnetobacteriaceae* group	-
Man4 Notung	Vertical inheritance from LCMA	HGT from *Magnetobacteriaceae* group	-
Man5 Ranger-DTL	Vertical inheritance from LCMA	HGT from *Magnetobacteriaceae* group	-
Man5 Notung	Vertical inheritance from LCMA	Vertical inheritance from LCMA	-
Man6 Ranger-DTL	HGT from LCDA	Vertical inheritance from LCMA	HGT from LCDA
Man6 Notung	HGT from LCDA	Vertical inheritance from LCMA	HGT from LCDA
Mad2 Ranger-DTL	Vertical inheritance from LCMA	HGT from *Magnetobacteriaceae* group	HGT from *Magnetobacteriaceae* group
Mad2 Notung	Vertical inheritance from LCMA	HGT from *Magnetobacteriaceae* group	HGT from LCDA
Mad10 Ranger-DTL	Vertical inheritance from LCMA	HGT from *Magnetobacteriaceae* group	HGT from *Magnetobacteriaceae* group
Mad10 Notung	Vertical inheritance from LCMA	HGT from *Magnetobacteriaceae* group	HGT from *Magnetobacteriaceae* group
Mad23 Ranger-DTL	HGT from *Bdellovibrionota*	HGT from *Magnetobacteriaceae* group	HGT from *Magnetobacteriaceae* group
Mad23 Notung	HGT from *Thermodesulfobacteriota*	HGT from *Magnetobacteriaceae* group	HGT from *Magnetobacteriaceae* group
Mad24 Ranger-DTL	Vertical inheritance from LCMA	HGT from *Magnetobacteriaceae* group	HGT from *Magnetobacteriaceae* group
Mad24 Notung	HGT from *Thermodesulfobacteriota*	HGT from *Magnetobacteriaceae* group	Vertical inheritance from LCMA
Mad25 Ranger-DTL	Vertical inheritance from LCMA	HGT from *Magnetobacteriaceae* group	HGT from *Magnetobacteriaceae* group
Mad25 Notung	Vertical inheritance from LCMA	HGT from *Magnetobacteriaceae* group	HGT from *Thermodesulfobacteriota*
Mad26 Ranger-DTL	HGT from *Thermodesulfobacteriota*	HGT from *Magnetobacteriaceae* group	HGT from *Magnetobacteriaceae* group
Mad26 Notung	HGT from *Thermodesulfobacteriota*	HGT from *Magnetobacteriaceae* group	HGT from *Magnetobacteriaceae* group

**Analysis**	***Magnetobacteriaceae* group**	***Dissulfurispiraceae* group**	***man*-containing *Thermodesulfobacteriota* group**
Mad31 Ranger-DTL	Vertical inheritance from LCMA	HGT from *Magnetobacteriaceae* group	HGT from *Magnetobacteriaceae* group
Mad31 Notung	Vertical inheritance from LCMA	HGT from *Magnetobacteriaceae* group	HGT from *Magnetobacteriaceae* group
MamA Ranger-DTL	HGT from *Bdellovibrionota*/ *Hydrogenedentota*	HGT from *Magnetobacteriaceae* group	HGT from *Magnetobacteriaceae* group
MamA Notung	HGT from *Bdellovibrionota*	HGT from *Magnetobacteriaceae* group	HGT from *Magnetobacteriaceae* group
MamB Ranger-DTL	HGT from *man*-containing *Thermodesulfobacteriota* group	HGT from *Magnetobacteriaceae* group	HGT from *Riflebactetria*
MamB Notung	HGT from *Riflebactetria*	HGT from *Magnetobacteriaceae* group	HGT from *Magnetobacteriaceae* group
MamK Ranger-DTL	HGT from *Thermodesulfobacteriota*	HGT from *Magnetobacteriaceae* group	HGT from *Magnetobacteriaceae* group
MamK Notung	HGT from *Thermodesulfobacteriota*	HGT from *Magnetobacteriaceae* group	HGT from *Magnetobacteriaceae* group
MamM Ranger-DTL	HGT from *man*-containing *Thermodesulfobacteriota* group	HGT from *Magnetobacteriaceae* group	HGT from *Planctomycetota*
MamM Notung	HGT from *man*-containing *Thermodesulfobacteriota* group	HGT from *Magnetobacteriaceae* group	HGT from *Thermodesulfobacteriota*
MamP Ranger-DTL	HGT from *Placntomycetota*	HGT from *Magnetobacteriaceae* group	HGT from *Magnetobacteriaceae* group
MamP Notung	HGT from *Omnitrophota*	HGT from *Magnetobacteriaceae* group	HGT from *Magnetobacteriaceae* group
MamQ Ranger-DTL	HGT from *Riflebacteria*	HGT from *Magnetobacteriaceae* group	HGT from *Magnetobacteriaceae* group
MamQ Notung	HGT from *man*-containing *Thermodesulfobacteriota* group	HGT from *Magnetobacteriaceae* group	HGT from *Planctomycetota*
MamI Ranger-DTL	HGT from *man*-containing *Thermodesulfobacteriota* group	HGT from *Magnetobacteriaceae* group	HGT from *Omnitrophota*
MamI Notung	HGT from *Thermodesulfobacteriota*	HGT from *Magnetobacteriaceae* group	HGT from *Magnetobacteriaceae* group
MamE Ranger-DTL	HGT from *man*-containing *Thermodesulfobacteriota* group	HGT from *Magnetobacteriaceae* group	HGT from *Omnitrophota*
MamE Notung	HGT from *man*-containing *Thermodesulfobacteriota* group	HGT from *Magnetobacteriaceae* group	HGT from *Nitrospinota*
MamO-Cter Ranger-DTL	HGT from *Bdellovibrionota*	HGT from *Magnetobacteriaceae* group	HGT from *Magnetobacteriaceae* group
MamO-Cter Notung	HGT from *man*-containing *Thermodesulfobacteriota* group	HGT from *Magnetobacteriaceae* group	HGT from *Planctomycetota*
MamQ-2 Ranger-DTL	Vertical inheritance from LCMA	HGT from *Magnetobacteriaceae* group	HGT from *Magnetobacteriaceae* group
MamQ-2 Notung	Vertical inheritance from LCMA	HGT from *Magnetobacteriaceae* group	HGT from *Magnetobacteriaceae* group
**Concatenated Ranger-DTL**	HGT from *Bdellovibrionota*	HGT from *Magnetobacteriaceae* group	HGT from *Magnetobacteriaceae* group
**Concatenated Notung**	HGT from *man*-containing *Thermodesulfobacteriota* group	HGT from *Magnetobacteriaceae* group	HGT from *Bdellovibrionota*

Supplementary table S3. Reconstructed genomes statistics.

Attribute	LBB01		LBB02		LBB04	
	Value	% of Total	Value	% of Total	Value	% of Total
Genome size, bp	3,273,455	100.0	3,471,208	100.0	4,495,917	100.0
DNA coding, bp	2,939,340	89.8	3,089,132	89.0	3,786,226	84.2
DNA G+C, bp	1,373,588	42.0	1,629,784	47.0	2,267,373	50.4
DNA scaffolds	1	100.0	142	100.0	2,129	100.0
Total genes	3,132	100.0	3,355	100.0	5,601	100.0
Protein coding genes	3,021	96.5	3,298	98.3	5,379	96.0
RNA genes	56	1.8	37	1.1	34	0.6
Pseudo genes	55	1.8	20	0.6	188	3.4
Genes with function prediction	2,474	79.0	2,521	75.1	3,825	68.3
Genes assigned to COGs	2,373	75.8	2,445	72.9	4,051	72.3
Genes with Pfam domains	2,473	79.0	2,576	76.8	3,844	68.6
Genes with signal peptides	305	9.7	412	12.3	512	9.1
Genes with transmembrane helices	807	25.8	943	28.1	1284	22.9
CRISPR repeats	6	-	11	-	8	-
N_{50}, bp	3,273,455		44,214		2,222	
CheckM completeness, %	99.03		91.52		60.18	
CheckM contamination, %	0.91		0		0	
**GTDB affiliations**						
Phylum	*Nitrospirota*		*Nitrospirota*		*Desulfobacterota*	
Class	*Thermodesulfovibrionia*		*Thermodesulfovibrionia*		*Syntrophia*	
Order	*Thermodesulfovibrionales*		*Thermodesulfovibrionales*		*Syntrophales*	
Family	*Ca*. Magnetobacteriaceae		*Ca*. Magnetobacteriaceae		UBA2185	
Genus	-		HCH-1		-	
Species	-		-		-	

Supplementary table S4. AAI and POCP values between *Nitrospirota* genomes.

			POCP																																			
	№	Genome	1	2	3	4	5	6	7	8	9	10	11	12	13	14	15	16	17	18	19	20	21	22	23	24	25	26	27	28	29	30	31	32	33	34	35	36	37
	1	*Ca* . Magnetomonas plexicatena LBB01	100.0	76.7	82.0	79.4	81.8	59.0	60.4	60.9	61.3	61.4	46.4	51.1	48.3	58.3	57.9	51.3	53.8	55.4	55.2	52.9	43.3	54.8	55.5	55.2	49.6	54.6	54.0	51.6	52.2	33.1	45.4	40.0	33.0	32.6	34.0	33.9	32.9
	2	*Ca* . Magnetomonas sp. nDJH6bin1	85.4	100.0	82.4	79.5	81.9	55.4	57.2	56.5	56.7	56.8	44.0	48.1	45.7	52.7	52.2	48.0	50.2	51.3	51.5	49.7	40.3	50.6	51.0	50.7	45.8	50.4	49.5	49.1	48.0	31.7	42.2	37.2	31.1	30.4	32.2	31.5	30.8
	3	*Ca* . Magnetomonas sp. nDJH13bin19	86.3	94.3	100.0	94.3	97.3	59.8	61.9	59.5	59.9	60.0	47.0	50.2	48.4	57.2	56.9	51.1	53.1	54.3	55.6	53.4	42.1	54.6	55.1	54.7	48.3	53.8	53.0	53.1	51.1	32.5	44.9	40.2	33.0	32.3	34.0	34.0	32.5
	4	*Ca* . Magnetomonas sp. nDJH8bin8	86.2	94.1	99.9	100.0	93.4	58.4	61.0	58.6	58.8	59.0	50.4	48.7	46.8	55.8	55.8	49.3	52.9	53.2	54.1	52.0	40.7	54.2	54.0	53.6	47.0	52.5	51.7	52.7	49.8	31.3	43.5	38.3	31.4	30.5	32.2	32.1	30.6
	5	*Ca* . Magnetomonas sp. nDJH14bin5	86.2	94.2	100.0	99.9	100.0	59.4	61.0	59.0	59.4	59.5	46.3	49.7	47.8	56.6	56.3	50.7	52.5	53.9	54.7	52.7	42.7	53.8	54.6	54.2	47.9	53.3	52.3	52.8	50.4	32.4	44.1	39.7	32.7	32.3	33.6	33.8	32.4
	6	*Ca* . Magnetominusculus linsii LBB02	60.8	60.3	61.1	61.0	60.9	100.0	76.1	73.5	73.9	74.2	55.7	63.7	58.3	66.6	66.0	62.2	52.6	53.1	52.9	50.4	42.5	52.2	53.0	52.6	46.7	52.2	50.6	48.4	48.0	30.6	41.7	37.1	29.9	29.6	31.0	31.0	30.0
	7	*Ca* . Magnetominusculus sp. nDJH13bin15	61.5	61.7	62.3	62.1	62.1	77.3	100.0	73.9	74.6	74.8	61.0	65.4	61.5	66.4	66.4	64.0	54.0	54.8	55.7	53.1	41.8	53.6	54.4	54.0	48.6	53.5	52.2	52.2	50.0	31.2	44.0	39.3	31.8	31.5	33.3	32.9	32.0
	8	*Ca* . Magnetominusculus xianensis HCH-1	61.9	61.3	61.6	61.6	61.3	76.8	80.1	100.0	96.9	97.2	57.7	65.4	60.8	70.8	71.0	64.8	54.8	55.9	54.4	51.8	43.3	55.0	56.4	56.2	50.0	54.8	54.1	49.9	50.5	30.4	42.4	39.1	31.3	31.8	33.0	32.4	31.7
	9	*Ca.* Magnetominusculus xianensis nHCHbin2	61.8	61.2	61.6	61.4	61.3	76.8	80.2	100.0	100.0	99.2	58.1	65.8	60.7	71.5	71.7	65.3	54.8	55.7	54.4	51.7	43.2	55.1	56.5	56.3	50.0	54.8	54.0	50.3	50.5	30.4	42.4	39.0	31.0	31.6	32.8	32.3	31.7
	10	*Ca* . Magnetominusculus xianensis HCHbin1	61.7	61.1	61.5	61.4	61.3	76.8	80.1	100.0	100.0	100.0	58.2	66.0	61.0	71.5	71.9	65.6	54.8	55.8	54.4	51.7	43.3	55.2	56.6	56.3	50.0	54.9	54.1	50.2	50.7	30.4	42.5	38.9	31.1	31.6	32.8	32.3	31.6
	11	*Ca* . Magnetominusculus sp. nDJH14bin7	61.5	61.5	62.1	65.2	61.9	76.7	81.4	79.7	79.8	79.7	100.0	51.6	49.5	51.0	51.8	49.7	42.1	42.0	42.7	40.7	30.4	42.2	41.8	41.5	36.8	40.5	39.9	40.9	38.7	22.8	32.5	30.2	24.1	23.3	24.8	24.9	23.6
	12	*Ca* . Magnetominusculus sp. nDJH8bin13	60.7	60.4	60.9	60.8	60.8	76.1	80.0	78.7	78.9	78.9	86.5	100.0	60.9	58.6	59.4	58.3	44.9	45.7	45.5	44.5	39.0	43.8	44.8	44.6	40.8	44.8	44.4	43.0	40.8	27.8	35.9	33.4	26.3	26.4	27.4	27.7	26.5
	13	*Ca* . Magnetominusculus sp. nDJH13bin15	61.8	61.1	62.2	62.0	61.9	76.9	81.4	79.6	79.6	79.6	87.5	92.0	100.0	53.3	53.2	54.5	42.5	43.9	44.6	43.1	36.8	42.0	43.1	42.8	40.8	43.3	43.6	40.6	40.4	27.2	35.0	33.8	27.4	27.4	28.8	28.9	27.6
	14	*Ca* . Magnetominusculus sp. nMYbin6	61.5	60.5	61.2	60.9	61.0	73.9	76.6	77.1	77.2	77.2	76.6	75.8	76.5	100.0	92.8	58.8	53.3	54.2	51.4	48.7	42.5	52.7	53.6	53.2	47.7	53.3	52.2	47.6	46.7	29.2	39.2	36.9	29.8	29.4	31.1	30.7	29.7
	15	*Ca* . Magnetominusculus sp. MYbin6	61.8	60.7	61.4	61.1	61.2	74.0	76.7	77.1	77.1	77.1	76.5	75.7	76.5	99.9	100.0	58.0	53.1	53.6	51.3	48.5	42.0	52.8	53.6	53.2	47.7	53.0	51.8	47.5	46.5	29.2	39.7	36.4	28.7	28.5	30.2	29.9	28.5
	16	*Ca* . Magnetominusculus sp. nDJH5bin4	61.1	60.6	61.4	61.3	61.2	75.7	78.4	79.9	80.1	80.1	77.9	76.8	77.3	74.5	74.6	100.0	44.2	45.1	46.5	44.0	37.4	44.4	45.5	45.2	41.4	45.0	43.9	41.7	43.2	27.6	36.4	33.5	26.7	27.2	28.2	28.1	26.6
	17	*Ca* . Magnetobacterium sp. nMYbin4	59.7	59.6	60.0	60.1	59.8	59.9	60.7	60.5	60.4	60.4	60.2	58.9	60.5	60.4	60.4	59.6	100.0	90.8	69.2	66.5	54.8	71.3	71.4	71.3	64.8	70.8	69.8	49.6	50.3	29.3	40.9	37.6	30.8	29.9	31.8	31.1	29.8
	18	*Ca* . Magnetobacterium sp. MYbinv3	60.0	59.8	60.2	60.1	60.1	60.0	60.8	60.7	60.6	60.6	60.4	59.2	60.7	60.6	60.6	59.9	99.8	100.0	71.7	68.8	56.9	72.1	73.4	72.8	66.6	72.9	72.2	51.1	51.8	30.0	41.5	38.5	32.0	31.2	33.1	32.6	31.1
AAI	19	*Ca* . Magnetobacterium sp. nDJH15bin2	59.8	59.5	59.9	59.9	59.5	59.6	60.8	60.3	60.3	60.2	60.6	59.1	60.7	59.8	59.9	60.1	75.7	75.8	100.0	88.1	58.9	73.3	75.2	74.5	67.9	73.8	71.7	53.1	51.4	30.5	41.6	37.9	31.2	29.9	32.2	31.4	30.6
	20	*Ca* . Magnetobacterium sp. nDJH13bin1	60.0	59.7	60.1	60.2	59.9	59.7	61.0	60.7	60.6	60.6	60.6	59.3	61.0	60.0	60.0	60.1	75.5	75.7	99.2	100.0	57.9	70.5	72.2	71.6	64.7	70.7	69.2	50.8	49.2	28.9	39.6	36.8	30.0	28.6	31.2	30.4	29.7
	21	*Ca* . Magnetobacterium sp. nMYbin3	58.1	58.2	58.0	58.1	57.8	58.9	59.0	59.1	59.1	59.1	58.7	58.4	58.5	59.0	59.2	57.7	74.2	74.4	80.9	80.5	100.0	57.7	59.4	59.1	56.6	61.6	58.0	38.9	39.6	25.7	30.8	28.9	25.0	24.8	25.1	24.5	24.6
	22	*Ca* . Magnetobacterium cryptolimnobacter XYR	60.0	59.3	59.8	59.8	59.6	60.0	60.7	60.7	60.6	60.6	60.2	59.2	60.7	60.2	60.4	59.8	74.8	75.1	80.5	80.5	83.7	100.0	94.3	93.7	75.2	80.8	79.4	49.6	50.1	29.0	39.7	36.8	28.9	28.7	30.5	30.0	29.0
	23	*Ca* . Magnetobacterium sp. DC0425bin1	60.1	59.4	59.8	59.7	59.6	60.1	60.6	60.7	60.6	60.6	60.3	59.3	60.7	60.3	60.4	59.7	74.8	74.8	80.5	80.4	83.5	99.5	100.0	97.8	76.4	82.4	80.7	50.8	50.9	29.6	40.2	37.3	29.7	29.7	31.3	30.7	29.8
	24	*Ca* . Magnetobacterium sp. nDC0425bin1	60.1	59.4	59.8	59.7	59.6	60.1	60.6	60.6	60.6	60.5	60.2	59.2	60.7	60.2	60.3	59.7	74.8	74.8	80.6	80.5	83.7	99.4	100.0	100.0	76.3	82.0	80.3	50.4	50.7	29.6	39.9	37.2	29.6	29.6	31.2	30.7	29.6
	25	*Ca* . Magnetobacterium sp. MYbin2	60.2	59.2	59.9	59.7	59.6	60.1	60.5	60.5	60.4	60.4	60.6	59.0	60.6	60.0	59.9	59.8	75.0	74.9	80.7	80.4	83.1	92.1	92.0	92.0	100.0	85.0	84.7	44.9	45.7	28.3	36.8	32.6	26.9	26.3	27.6	27.5	26.8
	26	*Ca* . Magnetobacterium sp. nMYbin2	59.8	59.2	59.6	59.5	59.4	59.8	60.5	60.4	60.3	60.3	60.1	59.2	60.2	60.0	59.9	59.5	75.0	75.1	80.8	80.5	83.7	92.5	92.5	92.5	99.5	100.0	89.5	49.6	49.5	29.9	39.9	35.8	29.1	28.9	30.3	29.7	28.8
	27	*Ca* . Magnetobacterium casensis MYR-1	60.3	59.8	60.2	60.0	60.0	60.4	60.9	61.1	61.0	61.0	60.8	59.7	61.1	60.4	60.4	60.1	75.1	75.2	80.9	80.7	83.7	92.5	92.4	92.5	99.7	99.8	100.0	49.0	48.9	29.5	39.7	36.4	29.9	29.6	31.1	30.7	29.8
	28	Magnetobacteriaceae bacterium nDJH13bin3	60.2	59.8	60.3	60.8	60.1	59.6	61.2	60.5	60.2	60.3	61.0	59.7	60.6	59.8	60.2	59.7	61.7	61.8	61.3	61.5	59.6	61.3	61.2	61.2	61.4	61.2	61.8	100.0	45.0	27.7	38.4	34.9	27.8	27.1	28.8	28.7	27.3
	29	Ca. Magnetomicrobium cryptolimnococcus XYC	57.8	57.0	57.6	57.4	57.3	56.6	57.6	57.5	57.5	57.4	57.1	56.2	57.4	56.8	56.9	56.9	57.5	57.8	57.4	57.4	55.3	57.2	57.2	57.2	57.2	57.1	57.5	57.1	100.0	31.2	42.1	38.1	31.9	31.7	33.4	33.1	32.6
	30	Thermodesulfovibrionales bacterium nDJH8bin7	55.4	54.9	55.2	55.2	55.0	55.4	55.5	55.5	55.5	55.5	55.0	53.9	54.7	54.8	54.8	55.5	55.5	55.7	55.0	55.1	53.8	54.8	55.0	55.0	55.0	54.8	55.0	54.2	54.6	100.0	71.6	29.3	23.3	24.0	25.0	24.9	23.9
	31	Thermodesulfovibrionales bacterium nDJH14bin9	56.1	55.9	56.2	56.1	55.9	55.9	56.5	56.6	56.5	56.5	56.0	54.6	55.8	55.5	55.5	55.9	56.3	56.6	56.4	56.3	54.3	56.0	56.1	56.1	56.1	55.8	56.2	55.6	55.5	99.6	100.0	39.7	32.1	32.1	33.8	33.6	32.9
	32	*Dissulfurispira thermophila* T55J	56.6	55.9	56.3	56.1	56.0	55.8	56.8	56.4	56.4	56.3	56.2	55.5	55.8	56.0	56.1	55.8	56.4	56.6	56.1	56.3	54.4	56.5	56.4	56.4	56.4	56.2	56.5	55.8	56.0	54.3	55.8	100.0	53.2	52.9	56.6	55.1	52.7
	33	*Thermodesulfovibrio hydrogeniphilus* DSM 18151	53.1	53.2	53.2	53.1	53.0	52.3	53.2	53.2	53.0	53.1	52.6	51.7	53.1	52.2	52.1	52.0	52.9	53.0	52.5	52.8	51.2	52.6	52.5	52.4	52.8	52.4	52.9	52.2	52.7	51.4	52.7	58.8	100.0	77.2	80.2	80.8	74.7
	34	*Thermodesulfovibrio* sp. N1	53.2	53.1	53.4	53.2	53.3	52.6	53.4	53.2	53.2	53.1	52.8	51.7	52.5	52.7	52.6	51.8	53.2	53.2	52.5	52.6	51.4	52.6	52.6	52.5	52.9	52.6	53.1	52.6	52.6	51.5	52.6	59.2	73.8	100.0	79.4	80.3	75.5
	35	*Thermodesulfovibrio yellowstonii* DSM 11347	52.9	52.9	53.1	52.8	52.8	52.4	53.1	52.9	53.0	52.9	52.7	51.9	52.9	52.4	52.4	51.7	53.2	53.3	52.5	52.6	51.4	52.7	52.6	52.6	52.6	52.2	52.9	52.3	52.7	51.2	52.7	59.1	74.9	76.8	100.0	84.2	78.9
	36	*Thermodesulfovibrio aggregans* TGE-P1	53.4	53.3	53.6	53.3	53.3	52.8	53.6	53.2	53.2	53.2	53.1	51.9	53.1	52.9	52.9	52.2	53.2	53.3	52.8	53.0	51.5	53.0	53.0	53.0	53.1	52.7	53.3	52.9	53.1	51.6	52.9	59.0	74.2	77.2	80.4	100.0	78.8
	37	*Thermodesulfovibrio thiophilus* DSM 17215	52.6	52.6	52.8	52.5	52.6	52.0	52.6	52.5	52.6	52.5	51.9	51.0	52.1	52.0	51.9	51.4	52.3	52.5	52.1	52.3	51.0	52.3	52.3	52.2	52.6	52.0	52.7	51.9	52.4	51.2	52.2	58.6	72.6	74.6	77.7	76.3	100.0

Supplementary table S5. ANI and dDDH values between LBB01 and closely related genomes.

Query genome	Reference genome	ANI, %	dDDH, %
Ca. Magnetomonas plexicatena LBB01	*Ca*. Magnetomonas sp. nDJH13bin19	83.1	27.3
Ca. Magnetomonas plexicatena LBB01	*Ca*. Magnetomonas sp. nDJH14bin5	83.1	27.3
Ca. Magnetomonas plexicatena LBB01	*Ca*. Magnetomonas sp. nDJH8bin8	83.0	27.3
Ca. Magnetomonas plexicatena LBB01	*Ca*. Magnetomonas sp. nDJH6bin1	82.7	27.3
Ca. Magnetomonas plexicatena LBB01	*Ca*. Magnetominusculus xianensis nHCHbin2	76.8	19.4
Ca. Magnetomonas plexicatena LBB01	*Ca*. Magnetominusculus xianensis HCH-1	76.7	20.0
Ca. Magnetomonas plexicatena LBB01	*Ca*. Magnetominusculus sp. nDJH8bin6	76.5	27.5
Ca. Magnetomonas plexicatena LBB01	*Ca*. Magnetominusculus xianensis HCHbin1	76.4	18.6
Ca. Magnetomonas plexicatena LBB01	*Ca*. Magnetominusculus sp. nMYbin6	76.1	18.9
Ca. Magnetomonas plexicatena LBB01	*Ca*. Magnetominusculus sp. MYbin6	76.0	19.8
Ca. Magnetomonas plexicatena LBB01	*Ca*. Magnetominusculus linsii LBB02	<75.0	17.1
Ca. Magnetomonas plexicatena LBB01	*Ca*. Magnetominusculus sp. nDJH14bin7	<75.0	24.3
Ca. Magnetomonas plexicatena LBB01	*Ca*. Magnetominusculus sp. nDJH5bin4	<75.0	23.3
Ca. Magnetomonas plexicatena LBB01	*Ca*. Magnetominusculus sp. nDJH13bin15	<75.0	18.6
Ca. Magnetomonas plexicatena LBB01	*Ca*. Magnetominusculus sp. nDJH8bin13	<75.0	17.9
Ca. Magnetomonas plexicatena LBB01	*Ca*. Magnetobacterium sp. MYbin2	<75.0	36.5
Ca. Magnetomonas plexicatena LBB01	*Ca*. Magnetobacterium casensis MYR-1	<75.0	31.0
Ca. Magnetomonas plexicatena LBB01	*Ca*. Magnetobacterium sp. nMYbin2	<75.0	29.8
Ca. Magnetomonas plexicatena LBB01	*Ca*. Magnetobacterium sp. nDJH15bin2	<75.0	28.8
Ca. Magnetomonas plexicatena LBB01	*Ca*. Magnetobacterium sp. nDC0425bin1	<75.0	26.7
Ca. Magnetomonas plexicatena LBB01	*Ca*. Magnetobacterium sp. nDJH13bin1	<75.0	26.7
Ca. Magnetomonas plexicatena LBB01	*Ca*. Magnetobacterium cryptolimnobacter XYR	<75.0	26.3
Ca. Magnetomonas plexicatena LBB01	*Ca*. Magnetobacterium sp. DC0425bin1	<75.0	26.0
Ca. Magnetomonas plexicatena LBB01	*Ca*. Magnetobacterium sp. nMYbin4	<75.0	20.9
Ca. Magnetomonas plexicatena LBB01	*Ca*. Magnetobacterium sp. MYbinv3	<75.0	19.5
Ca. Magnetomonas plexicatena LBB01	*Ca*. Magnetobacterium sp. nMYbin3	<75.0	16.5

Supplementary table S6. ANI and dDDH values between LBB02 and closely related genomes.

Query genome	Reference genome	ANI, %	dDDH, %
Ca. Magnetominusculus linsii LBB02	*Ca*. Magnetominusculus sp. nDJH8bin6	79.1	20.8
Ca. Magnetominusculus linsii LBB02	*Ca*. Magnetominusculus sp. nDJH13bin15	78.9	20.9
Ca. Magnetominusculus linsii LBB02	*Ca*. Magnetominusculus sp. nDJH14bin7	78.8	21.2
Ca. Magnetominusculus linsii LBB02	*Ca*. Magnetominusculus xianensis HCH-1	78.6	20.1
Ca. Magnetominusculus linsii LBB02	*Ca*. Magnetominusculus xianensis HCHbin1	78.6	20.1
Ca. Magnetominusculus linsii LBB02	*Ca*. Magnetominusculus xianensis nHCHbin2	78.5	20
Ca. Magnetominusculus linsii LBB02	*Ca*. Magnetominusculus sp. nDJH8bin13	78.5	20.3
Ca. Magnetominusculus linsii LBB02	*Ca*. Magnetominusculus sp. nDJH5bin4	78.0	19.8
Ca. Magnetominusculus linsii LBB02	*Ca*. Magnetominusculus sp. nMYbin6	77.3	18.9
Ca. Magnetominusculus linsii LBB02	*Ca*. Magnetominusculus sp. MYbin6	77.3	19.1
Ca. Magnetominusculus linsii LBB02	*Ca*. Magnetomonas sp. nDJH8bin8	76.2	18.5
Ca. Magnetominusculus linsii LBB02	*Ca*. Magnetomonas sp. nDJH14bin5	75.7	16.2
Ca. Magnetominusculus linsii LBB02	*Ca*. Magnetomonas sp. nDJH13bin19	75.7	16.3
Ca. Magnetominusculus linsii LBB02	*Ca*. Magnetomonas plexicatena LBB01	<75.0	17.1
Ca. Magnetominusculus linsii LBB02	*Ca*. Magnetomonas sp. nDJH6bin1	<75.0	17.4
Ca. Magnetominusculus linsii LBB02	*Ca*. Magnetobacterium sp. DC0425bin1	<75.0	17.3
Ca. Magnetominusculus linsii LBB02	*Ca*. Magnetobacterium sp. MYbin2	<75.0	19.4
Ca. Magnetominusculus linsii LBB02	*Ca*. Magnetobacterium sp. MYbinv3	<75.0	19.6
Ca. Magnetominusculus linsii LBB02	*Ca*. Magnetobacterium casensis MYR-1	<75.0	17
Ca. Magnetominusculus linsii LBB02	*Ca*. Magnetobacterium sp. nDC0425bin1	<75.0	17.1
Ca. Magnetominusculus linsii LBB02	*Ca*. Magnetobacterium sp. nDJH13bin1	<75.0	19
Ca. Magnetominusculus linsii LBB02	*Ca*. Magnetobacterium sp. nDJH15bin2	<75.0	15.9
Ca. Magnetominusculus linsii LBB02	*Ca*. Magnetobacterium sp. nMYbin2	<75.0	16.4
Ca. Magnetominusculus linsii LBB02	*Ca*. Magnetobacterium sp. nMYbin3	<75.0	14.7
Ca. Magnetominusculus linsii LBB02	*Ca*. Magnetobacterium sp. nMYbin4	<75.0	17.2
Ca. Magnetominusculus linsii LBB02	*Ca*. Magnetobacterium cryptolimnobacter XYR	<75.0	17.3

Supplementary table S7. Genomes used for the MGC genes search in the *Nitrospirota* phylum

ID	NCBI Organism Name	GTDB Taxonomy	GTDB species representative	NCBI type material
GCF_000284315.1	Leptospirillum ferrooxidans C2-3	p_Nitrospirota; c_Leptospirillia; o_Leptospirillales; f_Leptospirillaceae; g_Leptospirillum; s_Leptospirillum ferrooxidans	yes	no
GCF_000755505.1	Leptospirillum ferriphilum	p_Nitrospirota; c_Leptospirillia; o_Leptospirillales; f_Leptospirillaceae; g_Leptospirillum_A; s_Leptospirillum_A ferriphilum	yes	yes
GCF_000299235.1	Leptospirillum ferriphilum ML-04	p_Nitrospirota; c_Leptospirillia; o_Leptospirillales; f_Leptospirillaceae; g_Leptospirillum_A; s_Leptospirillum_A rubarum	yes	no
GCA_002387725.1	Nitrospirae bacterium UBA4572	p_Nitrospirota; c_Leptospirillia; o_Leptospirillales; f_Leptospirillaceae; g_UBA4572; s_UBA4572 sp002387725	yes	no
GCA_001803875.1	Nitrospirae bacterium RIFCSPHIGHO2_01_FULL_66_17	p_Nitrospirota; c_Nitrospiria; o_2-01-FULL-66-17; f_2-01-FULL-66-17; g_2-01-FULL-66-17; s_2-01-FULL-66-17 sp001803875	yes	no
GCA_001805245.1	Nitrospirae bacterium RIFCSPLOWO2_02_FULL_62_14	p_Nitrospirota; c_Nitrospiria; o_Nitrospirales; f_Nitrospiraceae; g_2-02-FULL-62-14; s_2-02-FULL-62-14 sp001805245	yes	no
GCA_001914955.1	Nitrospirae bacterium 13_2_20CM_2_62_8	p_Nitrospirota; c_Nitrospiria; o_Nitrospirales; f_Nitrospiraceae; g_40CM-3-62-11; s_40CM-3-62-11 sp001914955	yes	no
GCF_001458695.1	Candidatus Nitrospira inopinata	p_Nitrospirota; c_Nitrospiria; o_Nitrospirales; f_Nitrospiraceae; g_Nitrospira; s_Nitrospira inopinata	yes	no
GCF_001273775.1	Nitrospira moscoviensis	p_Nitrospirota; c_Nitrospiria; o_Nitrospirales; f_Nitrospiraceae; g_Nitrospira; s_Nitrospira moscoviensis	yes	yes
GCF_001458775.1	Candidatus Nitrospira nitrificans	p_Nitrospirota; c_Nitrospiria; o_Nitrospirales; f_Nitrospiraceae; g_Nitrospira; s_Nitrospira nitrificans	yes	no
GCF_001458735.1	Candidatus Nitrospira nitrosa	p_Nitrospirota; c_Nitrospiria; o_Nitrospirales; f_Nitrospiraceae; g_Nitrospira; s_Nitrospira nitrosa	yes	no
GCA_001464735.1	Nitrospira sp. Ga0074138	p_Nitrospirota; c_Nitrospiria; o_Nitrospirales; f_Nitrospiraceae; g_Nitrospira; s_Nitrospira sp001464735	yes	no
GCA_002083365.1	Nitrospira sp. SG-bin1	p_Nitrospirota; c_Nitrospiria; o_Nitrospirales; f_Nitrospiraceae; g_Nitrospira; s_Nitrospira sp002083365	yes	no
GCA_002083565.1	Nitrospira sp. ST-bin4	p_Nitrospirota; c_Nitrospiria; o_Nitrospirales; f_Nitrospiraceae; g_Nitrospira; s_Nitrospira sp002083565	yes	no
GCA_002254365.1	Nitrospira sp. UW-LDO-01	p_Nitrospirota; c_Nitrospiria; o_Nitrospirales; f_Nitrospiraceae; g_Nitrospira; s_Nitrospira sp002254365	yes	no
GCA_002331335.1	Nitrospira sp. UBA2082	p_Nitrospirota; c_Nitrospiria; o_Nitrospirales; f_Nitrospiraceae; g_Nitrospira; s_Nitrospira sp002331335	yes	no
GCA_002331625.1	Nitrospira sp. UBA2083	p_Nitrospirota; c_Nitrospiria; o_Nitrospirales; f_Nitrospiraceae; g_Nitrospira; s_Nitrospira sp002331625	yes	no
GCA_002420115.1	Nitrospira sp. UBA5698	p_Nitrospirota; c_Nitrospiria; o_Nitrospirales; f_Nitrospiraceae; g_Nitrospira; s_Nitrospira sp002420115	yes	no
GCA_002451055.1	Nitrospira sp. UBA6909	p_Nitrospirota; c_Nitrospiria; o_Nitrospirales; f_Nitrospiraceae; g_Nitrospira; s_Nitrospira sp002451055	yes	no
GCA_002869845.2	Nitrospira sp. CG24B	p_Nitrospirota; c_Nitrospiria; o_Nitrospirales; f_Nitrospiraceae; g_Nitrospira; s_Nitrospira sp002869845	yes	no
GCA_005116745.1	Nitrospira sp.	p_Nitrospirota; c_Nitrospiria; o_Nitrospirales; f_Nitrospiraceae; g_Nitrospira; s_Nitrospira sp005116745	yes	no
GCA_005116895.1	Nitrospira sp.	p_Nitrospirota; c_Nitrospiria; o_Nitrospirales; f_Nitrospiraceae; g_Nitrospira; s_Nitrospira sp005116895	yes	no
GCA_005116955.1	Nitrospira sp.	p_Nitrospirota; c_Nitrospiria; o_Nitrospirales; f_Nitrospiraceae; g_Nitrospira; s_Nitrospira sp005116955	yes	no
GCA_005116965.1	Nitrospira sp.	p_Nitrospirota; c_Nitrospiria; o_Nitrospirales; f_Nitrospiraceae; g_Nitrospira; s_Nitrospira sp005116965	yes	no
GCA_005239465.1	Nitrospira sp.	p_Nitrospirota; c_Nitrospiria; o_Nitrospirales; f_Nitrospiraceae; g_Nitrospira; s_Nitrospira sp005239465	yes	no
GCA_005793285.1	Nitrospiraceae bacterium	p_Nitrospirota; c_Nitrospiria; o_Nitrospirales; f_Nitrospiraceae; g_Nitrospira; s_Nitrospira sp005793285	yes	no
GCF_000196815.1	Nitrospira defluvii	p_Nitrospirota; c_Nitrospiria; o_Nitrospirales; f_Nitrospiraceae; g_Nitrospira_A; s_Nitrospira_A defluvii	yes	no
GCA_001567445.1	Nitrospira sp. OLB3	p_Nitrospirota; c_Nitrospiria; o_Nitrospirales; f_Nitrospiraceae; g_Nitrospira_A; s_Nitrospira_A sp001567445	yes	no
GCA_003456605.1	Nitrospira sp.	p_Nitrospirota; c_Nitrospiria; o_Nitrospirales; f_Nitrospiraceae; g_Nitrospira_A; s_Nitrospira_A sp003456605	yes	no
GCF_900170025.1	Nitrospira sp. ND1	p_Nitrospirota; c_Nitrospiria; o_Nitrospirales; f_Nitrospiraceae; g_Nitrospira_A; s_Nitrospira_A sp900170025	yes	no
GCA_900696505.1	uncultured Nitrosospira sp.	p_Nitrospirota; c_Nitrospiria; o_Nitrospirales; f_Nitrospiraceae; g_Nitrospira_A; s_Nitrospira_A sp900696505	yes	no
GCF_900169565.1	Nitrospira japonica	p_Nitrospirota; c_Nitrospiria; o_Nitrospirales; f_Nitrospiraceae; g_Nitrospira_C; s_Nitrospira_C japonica	yes	no
GCA_002420105.1	Nitrospira sp. UBA5699	p_Nitrospirota; c_Nitrospiria; o_Nitrospirales; f_Nitrospiraceae; g_Nitrospira_C; s_Nitrospira_C sp002420105	yes	no
GCF_900403705.1	Nitrospira lenta	p_Nitrospirota; c_Nitrospiria; o_Nitrospirales; f_Nitrospiraceae; g_Nitrospira_D; s_Nitrospira_D lenta	yes	no
GCA_002083555.1	Nitrospira sp. ST-bin5	p_Nitrospirota; c_Nitrospiria; o_Nitrospirales; f_Nitrospiraceae; g_Nitrospira_D; s_Nitrospira_D sp002083555	yes	no
GCA_002435325.1	Nitrospira sp. UBA6493	p_Nitrospirota; c_Nitrospiria; o_Nitrospirales; f_Nitrospiraceae; g_Nitrospira_D; s_Nitrospira_D sp002435325	yes	no
GCA_002869855.2	Nitrospira sp. CG24D	p_Nitrospirota; c_Nitrospiria; o_Nitrospirales; f_Nitrospiraceae; g_Nitrospira_D; s_Nitrospira_D sp002869855	yes	no
GCA_005877775.1	Nitrospirae bacterium	p_Nitrospirota; c_Nitrospiria; o_Nitrospirales; f_Nitrospiraceae; g_NS-7; s_NS-7 sp005877775	yes	no
GCA_002737345.1	Nitrospirae bacterium	p_Nitrospirota; c_Nitrospiria; o_Nitrospirales; f_Nitrospiraceae; g_Palsa-1315; s_Palsa-1315 sp002737345	yes	no
GCA_002869885.2	Nitrospira sp. CG24C	p_Nitrospirota; c_Nitrospiria; o_Nitrospirales; f_Nitrospiraceae; g_Palsa-1315; s_Palsa-1315 sp002869885	yes	no
GCA_002869895.2	Nitrospira sp. CG24E	p_Nitrospirota; c_Nitrospiria; o_Nitrospirales; f_Nitrospiraceae; g_Palsa-1315; s_Palsa-1315 sp002869895	yes	no
GCA_002869925.2	Nitrospira sp. CG24A	p_Nitrospirota; c_Nitrospiria; o_Nitrospirales; f_Nitrospiraceae; g_Palsa-1315; s_Palsa-1315 sp002869925	yes	no
GCA_003135435.1	Nitrospira sp.	p_Nitrospirota; c_Nitrospiria; o_Nitrospirales; f_Nitrospiraceae; g_Palsa-1315; s_Palsa-1315 sp003135435	yes	no
GCA_005116775.1	Nitrospira sp.	p_Nitrospirota; c_Nitrospiria; o_Nitrospirales; f_Nitrospiraceae; g_Palsa-1315; s_Palsa-1315 sp005116775	yes	no
GCA_005116815.1	Nitrospira sp.	p_Nitrospirota; c_Nitrospiria; o_Nitrospirales; f_Nitrospiraceae; g_Palsa-1315; s_Palsa-1315 sp005116815	yes	no
GCA_005116835.1	Nitrospira sp.	p_Nitrospirota; c_Nitrospiria; o_Nitrospirales; f_Nitrospiraceae; g_Palsa-1315; s_Palsa-1315 sp005116835	yes	no
GCA_005116885.1	Nitrospira sp.	p_Nitrospirota; c_Nitrospiria; o_Nitrospirales; f_Nitrospiraceae; g_Palsa-1315; s_Palsa-1315 sp005116885	yes	no
GCA_005116945.1	Nitrospira sp.	p_Nitrospirota; c_Nitrospiria; o_Nitrospirales; f_Nitrospiraceae; g_Palsa-1315; s_Palsa-1315 sp005116945	yes	no
GCA_005239475.1	Nitrospira sp.	p_Nitrospirota; c_Nitrospiria; o_Nitrospirales; f_Nitrospiraceae; g_Palsa-1315; s_Palsa-1315 sp005239475	yes	no
GCA_005800255.1	Nitrospiraceae bacterium	p_Nitrospirota; c_Nitrospiria; o_Nitrospirales; f_Nitrospiraceae; g_Palsa-1315; s_Palsa-1315 sp005800255	yes	no
GCA_005877535.1	Nitrospirae bacterium	p_Nitrospirota; c_Nitrospiria; o_Nitrospirales; f_Nitrospiraceae; g_Palsa-1315; s_Palsa-1315 sp005877535	yes	no
GCA_005887945.1	Nitrospirae bacterium	p_Nitrospirota; c_Nitrospiria; o_Nitrospirales; f_Nitrospiraceae; g_Palsa-1315; s_Palsa-1315 sp005887945	yes	no
GCA_005116795.1	Nitrospira sp.	p_Nitrospirota; c_Nitrospiria; o_Nitrospirales; f_Nitrospiraceae; g_RSF151; s_RSF151 sp005116795	yes	no
GCA_004296865.1	Nitrospirae bacterium	p_Nitrospirota; c_Nitrospiria; o_Nitrospirales; f_Nitrospiraceae; g_SCVQ01; s_SCVQ01 sp004296865	yes	no
GCA_004296885.1	Nitrospirae bacterium	p_Nitrospirota; c_Nitrospiria; o_Nitrospirales; f_Nitrospiraceae; g_SYGV01; s_SYGV01 sp004296885	yes	no
GCA_005799365.1	Nitrospiraceae bacterium	p_Nitrospirota; c_Nitrospiria; o_Nitrospirales; f_Nitrospiraceae; g_SYGV01; s_SYGV01 sp005799365	yes	no

ID	NCBI Organism Name	GTDB Taxonomy	GTDB species representative	NCBI type material
GCA_005877525.1	Nitrospirae bacterium	p_Nitrospirota; c_Nitrospiria; o_Nitrospirales; f_NS-4; g_NS-11; s_NS-11 sp005877525	yes	no
GCA_005877505.1	Nitrospirae bacterium	p_Nitrospirota; c_Nitrospiria; o_Nitrospirales; f_NS-4; g_NS-12; s_NS-12 sp005877505	yes	no
GCA_005877565.1	Nitrospirae bacterium	p_Nitrospirota; c_Nitrospiria; o_Nitrospirales; f_NS-4; g_NS-12; s_NS-12 sp005877565	yes	no
GCA_005877615.1	Nitrospirae bacterium	p_Nitrospirota; c_Nitrospiria; o_Nitrospirales; f_NS-4; g_NS-12; s_NS-12 sp005877615	yes	no
GCA_005877855.1	Nitrospirae bacterium	p_Nitrospirota; c_Nitrospiria; o_Nitrospirales; f_NS-4; g_NS-12; s_NS-12 sp005877855	yes	no
GCA_005877815.1	Nitrospirae bacterium	p_Nitrospirota; c_Nitrospiria; o_Nitrospirales; f_NS-4; g_NS-4; s_NS-4 sp005877815	yes	no
GCA_004298335.1	Nitrospirae bacterium	p_Nitrospirota; c_Nitrospiria; o_Nitrospirales; f_NS-4; g_SCTG01; s_SCTG01 sp004298335	yes	no
GCA_002328825.1	Nitrospiraceae bacterium UBA2166	p_Nitrospirota; c_Nitrospiria; o_Nitrospirales; f_UBA2166; g_UBA2166; s_UBA2166 sp002328825	yes	no
GCA_002726235.1	Nitrospiraceae bacterium	p_Nitrospirota; c_Nitrospiria; o_Nitrospirales; f_UBA2166; g_UBA2166; s_UBA2166 sp002726235	yes	no
GCA_002238765.1	Nitrospira sp. bin75	p_Nitrospirota; c_Nitrospiria; o_Nitrospirales; f_UBA8639; g_Bin75; s_Bin75 sp002238765	yes	no
GCA_003695915.1	Nitrospirae bacterium	p_Nitrospirota; c_Nitrospiria; o_Nitrospirales; f_UBA8639; g_J017; s_J017 sp003695915	yes	no
GCA_003696975.1	Nitrospirae bacterium	p_Nitrospirota; c_Nitrospiria; o_Nitrospirales; f_UBA8639; g_J031; s_J031 sp003696975	yes	no
GCA_001643555.1	Nitrospirae bacterium SPGG5	p_Nitrospirota; c_Nitrospiria; o_Nitrospirales; f_UBA8639; g_SPGG5; s_SPGG5 sp001643555	yes	no
GCA_003228495.1	Nitrospirae bacterium	p_Nitrospirota; c_Nitrospiria; o_Nitrospirales; f_UBA8639; g_UBA8639; s_UBA8639 sp003228495	yes	no
GCA_003233615.1	Nitrospirae bacterium	p_Nitrospirota; c_Nitrospiria; o_Nitrospirales; f_UBA8639; g_UBA8639; s_UBA8639 sp003233615	yes	no
GCA_003235785.1	Nitrospirae bacterium	p_Nitrospirota; c_Nitrospiria; o_Nitrospirales; f_UBA8639; g_UBA8639; s_UBA8639 sp003235785	yes	no
GCA_003523945.1	Nitrospiraceae bacterium	p_Nitrospirota; c_Nitrospiria; o_Nitrospirales; f_UBA8639; g_UBA8639; s_UBA8639 sp003523945	yes	no
GCA_005239925.1	Nitrospira sp.	p_Nitrospirota; c_Nitrospiria; o_SBBL01; f_SBBA01; g_SBBA01; s_SBBA01 sp005239925	yes	no
GCA_005239745.1	Nitrospira sp.	p_Nitrospirota; c_Nitrospiria; o_SBBL01; f_SBBL01; g_SBBL01; s_SBBL01 sp005239745	yes	no
GCA_005239595.1	Nitrospira sp.	p_Nitrospirota; c_Nitrospiria; o_SBBL01; f_SBBT01; g_SBBT01; s_SBBT01 sp005239595	yes	no
GCA_004297235.1	Nitrospirae bacterium	p_Nitrospirota; c_Nitrospiria; o_SBBL01; f_SCUR01; g_SCUR01; s_SCUR01 sp004297235	yes	no
GCA_001803815.1	Nitrospirae bacterium RBG_19FT_COMBO_42_15	p_Nitrospirota; c_Nitrospiria_A; o_9FT-COMBO-42-15; f_9FT-COMBO-42-15; g_9FT-COMBO-42-15; s_9FT-COMBO-42-15 sp001803815	yes	no
GCA_001805205.1	Nitrospirae bacterium RIFCSPLOW2_12_42_9	p_Nitrospirota; c_Nitrospiria_A; o_HDB-SIOI813; f_HDB-SIOI813; g_HDB-SIOI813; s_HDB-SIOI813 sp001805205	yes	no
GCA_001805165.1	Nitrospirae bacterium RBG_16_43_11	p_Nitrospirota; c_Nitrospiria_A; o_HDB-SIOI813; f_HDB-SIOI813; g_RBG-16-43-11; s_RBG-16-43-11 sp001805165	yes	no
GCA_001803795.1	Nitrospirae bacterium RBG_16_64_22	p_Nitrospirota; c_RBG-16-64-22; o_RBG-16-64-22; f_RBG-16-64-22; g_RBG-16-64-22; s_RBG-16-64-22 sp001803795	yes	no
GCA_003235715.1	Nitrospirae bacterium	p_Nitrospirota; c_Thermodesulfovibrionia; o_SZUA-242; f_SZUA-242; g_SZUA-242; s_SZUA-242 sp003235715	yes	no
GCA_002897855.1	bacterium BMS3Bbin05	p_Nitrospirota; c_Thermodesulfovibrionia; o_Thermodesulfovibrionales; f_BMS3Bbin05; g_BMS3Bbin05; s_BMS3Bbin05 sp002897855	yes	no
GCA_002897935.1	bacterium BMS3Abin08	p_Nitrospirota; c_Thermodesulfovibrionia; o_Thermodesulfovibrionales; f_JdFR-85; g_BMS3Abin08; s_BMS3Abin08 sp002897935	yes	no
GCA_002898135.1	bacterium BMS3Bbin07	p_Nitrospirota; c_Thermodesulfovibrionia; o_Thermodesulfovibrionales; f_JdFR-85; g_BMS3Bbin07; s_BMS3Bbin07 sp002898135	yes	no
GCA_003232715.1	Nitrospirae bacterium	p_Nitrospirota; c_Thermodesulfovibrionia; o_Thermodesulfovibrionales; f_JdFR-85; g_BMS3Bbin07; s_BMS3Bbin07 sp003232715	yes	no
GCA_002011745.1	Nitrospirae bacterium JdFR-85	p_Nitrospirota; c_Thermodesulfovibrionia; o_Thermodesulfovibrionales; f_JdFR-85; g_JdFR-85; s_JdFR-85 sp002011745	yes	no
GCA_002011815.1	Nitrospirae bacterium JdFR-86	p_Nitrospirota; c_Thermodesulfovibrionia; o_Thermodesulfovibrionales; f_JdFR-86; g_JdFR-86; s_JdFR-86 sp002011815	yes	no
GCA_002011795.1	Nitrospirae bacterium JdFR-88	p_Nitrospirota; c_Thermodesulfovibrionia; o_Thermodesulfovibrionales; f_JdFR-88; g_JdFR-88; s_JdFR-88 sp002011795	yes	no
GCF_001541255.1	Nitrospirae bacterium HCH-1	p_Nitrospirota; c_Thermodesulfovibrionia; o_Thermodesulfovibrionales; f_Magnetobacteriaceae; g_HCH-1; s_HCH-1 sp001541255	yes	no
GCA_002753305.1	Nitrospirae bacterium MYbin6	p_Nitrospirota; c_Thermodesulfovibrionia; o_Thermodesulfovibrionales; f_Magnetobacteriaceae; g_HCH-1; s_HCH-1 sp002753305	yes	no
GCF_000714715.1	Candidatus Magnetobacterium casensis	p_Nitrospirota; c_Thermodesulfovibrionia; o_Thermodesulfovibrionales; f_Magnetobacteriaceae; g_Magnetobacterium; s_Magnetobacterium casensis	yes	no
GCA_002753395.1	Nitrospirae bacterium MYbinv3	p_Nitrospirota; c_Thermodesulfovibrionia; o_Thermodesulfovibrionales; f_Magnetobacteriaceae; g_Magnetobacterium; s_Magnetobacterium sp002753395	yes	no
GCA_002753685.1	Nitrospirae bacterium	p_Nitrospirota; c_Thermodesulfovibrionia; o_Thermodesulfovibrionales; f_Magnetobacteriaceae; g_Magnetobacterium; s_Magnetobacterium sp002753685	yes	no
GCA_002780895.1	Nitrospirae bacterium CG02_land_8_20_14_3_00_41_53	p_Nitrospirota; c_Thermodesulfovibrionia; o_Thermodesulfovibrionales; f_SM23-35; g_0-14-3-00-41-53; s_0-14-3-00-41-53 sp002780895	yes	no
GCA_001805125.1	Nitrospirae bacterium RBG_13_39_12	p_Nitrospirota; c_Thermodesulfovibrionia; o_Thermodesulfovibrionales; f_SM23-35; g_RBG-13-39-12; s_RBG-13-39-12 sp001805125	yes	no
GCA_002328665.1	Nitrospiraceae bacterium UBA2194	p_Nitrospirota; c_Thermodesulfovibrionia; o_Thermodesulfovibrionales; f_SM23-35; g_UBA2194; s_UBA2194 sp002328665	yes	no
GCF_001514535.1	Thermodesulfovibrio aggregans	p_Nitrospirota; c_Thermodesulfovibrionia; o_Thermodesulfovibrionales; f_Thermodesulfovibrionaceae; g_Thermodesulfovibrio; s_aggregans	yes	yes
GCA_002878055.1	Thermodesulfovibrio aggregans	p_Nitrospirota; c_Thermodesulfovibrionia; o_Thermodesulfovibrionales; f_Thermodesulfovibrionaceae; g_Thermodesulfovibrio; s_aggregans_A	yes	no
GCF_001707915.1	Thermodesulfovibrio sp. N1	p_Nitrospirota; c_Thermodesulfovibrionia; o_Thermodesulfovibrionales; f_Thermodesulfovibrionaceae; g_Thermodesulfovibrio; s_sp001707915	yes	no
GCA_002339325.1	Nitrospiraceae bacterium UBA2600	p_Nitrospirota; c_Thermodesulfovibrionia; o_Thermodesulfovibrionales; f_Thermodesulfovibrionaceae; g_Thermodesulfovibrio; s_sp002339325	yes	no
GCF_000423865.1	Thermodesulfovibrio thiophilus DSM 17215	p_Nitrospirota; c_Thermodesulfovibrionia; o_Thermodesulfovibrionales; f_Thermodesulfovibrionaceae; g_Thermodesulfovibrio; s_thiophilus	yes	yes
GCF_000020985.1	Thermodesulfovibrio yellowstonii DSM 11347	p_Nitrospirota; c_Thermodesulfovibrionia; o_Thermodesulfovibrionales; f_Thermodesulfovibrionaceae; g_Thermodesulfovibrio; s_yellowstonii	yes	yes
GCA_004298625.1	Nitrospirae bacterium	p_Nitrospirota; c_Thermodesulfovibrionia; o_Thermodesulfovibrionales; f_UBA1546; g_SCSY01; s_SCSY01 sp004298625	yes	no
GCA_001805105.1	Nitrospirae bacterium GWF2_44_13	p_Nitrospirota; c_Thermodesulfovibrionia; o_Thermodesulfovibrionales; f_UBA1546; g_UBA1546; s_UBA1546 sp001805105	yes	no
GCA_001871685.1	Nitrospirae bacterium CG1_02_44_142	p_Nitrospirota; c_Thermodesulfovibrionia; o_Thermodesulfovibrionales; f_UBA1546; g_UBA1546; s_UBA1546 sp001871685	yes	no
GCA_003535475.1	Nitrospiraceae bacterium	p_Nitrospirota; c_Thermodesulfovibrionia; o_Thermodesulfovibrionales; f_UBA1546; g_UBA1546; s_UBA1546 sp003535475	yes	no
GCA_003599795.1	Nitrospiraceae bacterium	p_Nitrospirota; c_Thermodesulfovibrionia; o_Thermodesulfovibrionales; f_UBA1546; g_UBA1546; s_UBA1546 sp003599795	yes	no
GCA_001803645.1	Nitrospirae bacterium GWC2_42_7	p_Nitrospirota; c_Thermodesulfovibrionia; o_Thermodesulfovibrionales; f_UBA6898; g_GWC2-42-7; s_GWC2-42-7 sp001803645	yes	no
GCA_002839535.1	Nitrospira bacterium HGW-Nitrospira-1	p_Nitrospirota; c_Thermodesulfovibrionia; o_Thermodesulfovibrionales; f_UBA6898; g_GW-Nitrospira-1; s_GW-Nitrospira-1 sp002839535	yes	no
GCA_003153275.1	Nitrospiraceae bacterium	p_Nitrospirota; c_Thermodesulfovibrionia; o_Thermodesulfovibrionales; f_UBA6898; g_PALSA-1316; s_PALSA-1316 sp003153275	yes	no
GCA_004321925.1	Nitrospirae bacterium	p_Nitrospirota; c_Thermodesulfovibrionia; o_Thermodesulfovibrionales; f_UBA6898; g_PALSA-1316; s_PALSA-1316 sp004321925	yes	no
GCF_900302705.1	Candidatus Sulfobium mesophilum	p_Nitrospirota; c_Thermodesulfovibrionia; o_Thermodesulfovibrionales; f_UBA6898; g_Sulfobium; s_Sulfobium mesophilum	yes	no
GCA_002448975.1	Nitrospiraceae bacterium UBA6898	p_Nitrospirota; c_Thermodesulfovibrionia; o_Thermodesulfovibrionales; f_UBA6898; g_UBA6898; s_UBA6898 sp002448975	yes	no

ID	NCBI Organism Name	GTDB Taxonomy	GTDB species representative	NCBI type material
GCA_002451165.1	Nitrospiraceae bacterium UBA6905	p_Nitrospirota; c_Thermodesulfovibrionia; o_Thermodesulfovibrionales; f_UBA6898; g_UBA6898; s_UBA6898 sp002451165	yes	no
GCA_003252075.1	Nitrospirae bacterium	p_Nitrospirota; c_Thermodesulfovibrionia; o_Thermodesulfovibrionales; f_UBA6898; g_UBA6898; s_UBA6898 sp003252075	yes	no
GCA_003453735.1	Nitrospiraceae bacterium	p_Nitrospirota; c_Thermodesulfovibrionia; o_Thermodesulfovibrionales; f_UBA9159; g_UBA9159; s_UBA9159 sp003453735	yes	no
GCA_003170655.1	Nitrospiraceae bacterium	p_Nitrospirota; c_Thermodesulfovibrionia; o_Thermodesulfovibrionales; f_UBA9935; g_Fen-1308; s_Fen-1308 sp003170655	yes	no
GCA_002634385.1	Nitrospira sp.	p_Nitrospirota; c_Thermodesulfovibrionia; o_Thermodesulfovibrionales; f_UBA9935; g_GCA-2634385; s_GCA-2634385 sp002634385	yes	no
GCA_001803635.1	Nitrospirae bacterium GWB2_47_37	p_Nitrospirota; c_Thermodesulfovibrionia; o_Thermodesulfovibrionales; f_UBA9935; g_GWB2-47-37; s_GWB2-47-37 sp001803635	yes	no
GCA_002753335.1	Nitrospirae bacterium MYbin3	p_Nitrospirota; c_Thermodesulfovibrionia; o_Thermodesulfovibrionales; f_UBA9935; g_MYbin3; s_MYbin3 sp002753335	yes	no
GCA_004321915.1	Nitrospirae bacterium	p_Nitrospirota; c_Thermodesulfovibrionia; o_Thermodesulfovibrionales; f_UBA9935; g_MYbin3; s_MYbin3 sp004321915	yes	no
GCA_002299835.1	Nitrospiraceae bacterium UBA665	p_Nitrospirota; c_Thermodesulfovibrionia; o_Thermodesulfovibrionales; f_UBA9935; g_UBA665; s_UBA665 sp002299835	yes	no
GCA_002897775.1	bacterium BMS3Bbin08	p_Nitrospirota; c_Thermodesulfovibrionia; o_UBA6902; f_BMS3Bbin08; g_BMS3Bbin08; s_BMS3Bbin08 sp002897775	yes	no
GCA_002011735.1	Nitrospirae bacterium JdFR-81	p_Nitrospirota; c_Thermodesulfovibrionia; o_UBA6902; f_JdFR-81; g_JdFR-81; s_JdFR-81 sp002011735	yes	no
GCA_002897895.1	bacterium BMS3Abin06	p_Nitrospirota; c_Thermodesulfovibrionia; o_UBA6902; f_UBA6902; g_BMS3ABIN06; s_BMS3ABIN06 sp002897895	yes	no
GCA_002897915.1	bacterium BMS3Bbin09	p_Nitrospirota; c_Thermodesulfovibrionia; o_UBA6902; f_UBA6902; g_BMS3Abin09; s_BMS3Abin09 sp002897915	yes	no
GCA_003354025.1	Nitrospirae bacterium	p_Nitrospirota; c_Thermodesulfovibrionia; o_UBA6902; f_UBA6902; g_Glo-13; s_Glo-13 sp003354025	yes	no
GCA_003599505.1	Nitrospiraceae bacterium	p_Nitrospirota; c_Thermodesulfovibrionia; o_UBA6902; f_UBA6902; g_SURF-11; s_SURF-11 sp003599505	yes	no
GCA_003599425.1	Nitrospiraceae bacterium	p_Nitrospirota; c_Thermodesulfovibrionia; o_UBA6902; f_UBA6902; g_SURF-23; s_SURF-23 sp003599425	yes	no
GCA_003599275.1	Nitrospiraceae bacterium	p_Nitrospirota; c_Thermodesulfovibrionia; o_UBA6902; f_UBA6902; g_SURF-45; s_SURF-45 sp003599275	yes	no
GCA_002451135.1	Nitrospiraceae bacterium UBA6902	p_Nitrospirota; c_Thermodesulfovibrionia; o_UBA6902; f_UBA6902; g_UBA6902; s_UBA6902 sp002451135	yes	no
GCA_003599025.1	Nitrospiraceae bacterium	p_Nitrospirota; c_Thermodesulfovibrionia; o_UBA6902; f_UBA6902; g_UBA6902; s_UBA6902 sp003599025	yes	no
GCA_001803705.1	Nitrospirae bacterium GWC2_56_14	p_Nitrospirota; c_UBA9217; o_UBA9217; f_UBA9217; g_GWC2-56-14; s_GWC2-56-14 sp001803705	yes	no
GCA_001805055.1	Nitrospirae bacterium GWC2_57_13	p_Nitrospirota; c_UBA9217; o_UBA9217; f_UBA9217; g_GWC2-57-13; s_GWC2-57-13 sp001805055	yes	no
GCA_003454665.1	Nitrospiraceae bacterium	p_Nitrospirota; c_UBA9217; o_UBA9217; f_UBA9217; g_UBA9217; s_UBA9217 sp003454665	yes	no
GCF_900198525.1	Leptospirillum ferriphilum	p_Nitrospirota; c_Leptospirillia; o_Leptospirillales; f_Leptospirillaceae; g_Leptospirillum_A; s_Leptospirillum_A ferriphilum	no	no
GCA_000205145.2	Leptospirillum rubarum	p_Nitrospirota; c_Leptospirillia; o_Leptospirillales; f_Leptospirillaceae; g_Leptospirillum_A; s_Leptospirillum_A rubarum	no	no
GCA_000262365.1	Leptospirillum sp. Group II 'C75'	p_Nitrospirota; c_Leptospirillia; o_Leptospirillales; f_Leptospirillaceae; g_Leptospirillum_A; s_Leptospirillum_A rubarum	no	no
GCA_002386985.1	Leptospirillum ferriphilum	p_Nitrospirota; c_Leptospirillia; o_Leptospirillales; f_Leptospirillaceae; g_Leptospirillum_A; s_Leptospirillum_A rubarum	no	no
GCA_002420195.1	Leptospirillum ferriphilum	p_Nitrospirota; c_Leptospirillia; o_Leptospirillales; f_Leptospirillaceae; g_Leptospirillum_A; s_Leptospirillum_A rubarum	no	no
GCA_002455015.1	Leptospirillum ferriphilum	p_Nitrospirota; c_Leptospirillia; o_Leptospirillales; f_Leptospirillaceae; g_Leptospirillum_A; s_Leptospirillum_A rubarum	no	no
GCA_002455035.1	Leptospirillum ferriphilum	p_Nitrospirota; c_Leptospirillia; o_Leptospirillales; f_Leptospirillaceae; g_Leptospirillum_A; s_Leptospirillum_A rubarum	no	no
GCA_002455075.1	Leptospirillum ferriphilum	p_Nitrospirota; c_Leptospirillia; o_Leptospirillales; f_Leptospirillaceae; g_Leptospirillum_A; s_Leptospirillum_A rubarum	no	no
GCA_002455095.1	Leptospirillum ferriphilum	p_Nitrospirota; c_Leptospirillia; o_Leptospirillales; f_Leptospirillaceae; g_Leptospirillum_A; s_Leptospirillum_A rubarum	no	no
GCA_002455445.1	Leptospirillum ferriphilum	p_Nitrospirota; c_Leptospirillia; o_Leptospirillales; f_Leptospirillaceae; g_Leptospirillum_A; s_Leptospirillum_A rubarum	no	no
GCA_002469705.1	Leptospirillum ferriphilum	p_Nitrospirota; c_Leptospirillia; o_Leptospirillales; f_Leptospirillaceae; g_Leptospirillum_A; s_Leptospirillum_A rubarum	no	no
GCA_002470195.1	Leptospirillum ferriphilum	p_Nitrospirota; c_Leptospirillia; o_Leptospirillales; f_Leptospirillaceae; g_Leptospirillum_A; s_Leptospirillum_A rubarum	no	no
GCA_002470665.1	Leptospirillum ferriphilum	p_Nitrospirota; c_Leptospirillia; o_Leptospirillales; f_Leptospirillaceae; g_Leptospirillum_A; s_Leptospirillum_A rubarum	no	no
GCA_002500075.1	Leptospirillum ferriphilum	p_Nitrospirota; c_Leptospirillia; o_Leptospirillales; f_Leptospirillaceae; g_Leptospirillum_A; s_Leptospirillum_A rubarum	no	no
GCA_002500715.1	Leptospirillum ferriphilum	p_Nitrospirota; c_Leptospirillia; o_Leptospirillales; f_Leptospirillaceae; g_Leptospirillum_A; s_Leptospirillum_A rubarum	no	no
GCF_000695975.1	Leptospirillum ferriphilum YSK	p_Nitrospirota; c_Leptospirillia; o_Leptospirillales; f_Leptospirillaceae; g_Leptospirillum_A; s_Leptospirillum_A rubarum	no	no
GCF_001186405.1	Leptospirillum sp. Group II 'CF-1'	p_Nitrospirota; c_Leptospirillia; o_Leptospirillales; f_Leptospirillaceae; g_Leptospirillum_A; s_Leptospirillum_A rubarum	no	no
GCF_001280545.1	Leptospirillum ferriphilum	p_Nitrospirota; c_Leptospirillia; o_Leptospirillales; f_Leptospirillaceae; g_Leptospirillum_A; s_Leptospirillum_A rubarum	no	no
GCF_002002505.1	Leptospirillum ferriphilum	p_Nitrospirota; c_Leptospirillia; o_Leptospirillales; f_Leptospirillaceae; g_Leptospirillum_A; s_Leptospirillum_A rubarum	no	no
GCF_002002665.1	Leptospirillum ferriphilum	p_Nitrospirota; c_Leptospirillia; o_Leptospirillales; f_Leptospirillaceae; g_Leptospirillum_A; s_Leptospirillum_A rubarum	no	no
GCA_002386965.1	Nitrospirae bacterium UBA4574	p_Nitrospirota; c_Leptospirillia; o_Leptospirillales; f_Leptospirillaceae; g_UBA4572; s_UBA4572 sp002387725	no	no
GCA_002420165.1	Nitrospirae bacterium UBA5696	p_Nitrospirota; c_Leptospirillia; o_Leptospirillales; f_Leptospirillaceae; g_UBA4572; s_UBA4572 sp002387725	no	no
GCA_002454995.1	Nitrospirae bacterium UBA6678	p_Nitrospirota; c_Leptospirillia; o_Leptospirillales; f_Leptospirillaceae; g_UBA4572; s_UBA4572 sp002387725	no	no
GCA_002455055.1	Nitrospirae bacterium UBA6675	p_Nitrospirota; c_Leptospirillia; o_Leptospirillales; f_Leptospirillaceae; g_UBA4572; s_UBA4572 sp002387725	no	no
GCA_002470685.1	bacterium UBA7390	p_Nitrospirota; c_Leptospirillia; o_Leptospirillales; f_Leptospirillaceae; g_UBA4572; s_UBA4572 sp002387725	no	no
GCA_002500645.1	Nitrospirae bacterium UBA7871	p_Nitrospirota; c_Leptospirillia; o_Leptospirillales; f_Leptospirillaceae; g_UBA4572; s_UBA4572 sp002387725	no	no
GCA_001805225.1	Nitrospirae bacterium RIFCSPLOWO2_01_FULL_62_17	p_Nitrospirota; c_Nitrospiria; o_Nitrospirales; f_Nitrospiraceae; g_2-02-FULL-62-14; s_2-02-FULL-62-14 sp001805245	no	no
GCA_001914545.1	Nitrospirae bacterium 13_2_20CM_62_7	p_Nitrospirota; c_Nitrospiria; o_Nitrospirales; f_Nitrospiraceae; g_40CM-3-62-11; s_40CM-3-62-11 sp001914955	no	no
GCA_001918075.1	Nitrospirae bacterium 13_1_40CM_4_62_6	p_Nitrospirota; c_Nitrospiria; o_Nitrospirales; f_Nitrospiraceae; g_40CM-3-62-11; s_40CM-3-62-11 sp001914955	no	no
GCA_001918505.1	Nitrospirae bacterium 13_1_40CM_3_62_11	p_Nitrospirota; c_Nitrospiria; o_Nitrospirales; f_Nitrospiraceae; g_40CM-3-62-11; s_40CM-3-62-11 sp001914955	no	no
GCA_001920515.1	Nitrospirae bacterium 13_1_20CM_2_62_14	p_Nitrospirota; c_Nitrospiria; o_Nitrospirales; f_Nitrospiraceae; g_40CM-3-62-11; s_40CM-3-62-11 sp001914955	no	no
GCA_005877575.1	Nitrospirae bacterium	p_Nitrospirota; c_Nitrospiria; o_Nitrospirales; f_Nitrospiraceae; g_40CM-3-62-11; s_40CM-3-62-11 sp001914955	no	no
GCA_005877605.1	Nitrospirae bacterium	p_Nitrospirota; c_Nitrospiria; o_Nitrospirales; f_Nitrospiraceae; g_40CM-3-62-11; s_40CM-3-62-11 sp001914955	no	no
GCA_002420045.1	Nitrospira sp. UBA5702	p_Nitrospirota; c_Nitrospiria; o_Nitrospirales; f_Nitrospiraceae; g_Nitrospira; s_Nitrospira sp002420115	no	no
GCA_003529185.1	Nitrospira sp.	p_Nitrospirota; c_Nitrospiria; o_Nitrospirales; f_Nitrospiraceae; g_Nitrospira; s_Nitrospira sp002420115	no	no
GCA_002083355.1	Nitrospira sp. HN-bin3	p_Nitrospirota; c_Nitrospiria; o_Nitrospirales; f_Nitrospiraceae; g_Nitrospira; s_Nitrospira sp900078515	no	no

ID	NCBI Organism Name	GTDB Taxonomy	GTDB species representative	NCBI type material
GCA_002483475.1	Nitrospira sp. UBA7655	p_Nitrospirota; c_Nitrospiria; o_Nitrospirales; f_Nitrospiraceae; g_Nitrospira; s_Nitrospira sp900078515	no	no
GCA_900696515.1	uncultured Nitrosospira sp.	p_Nitrospirota; c_Nitrospiria; o_Nitrospirales; f_Nitrospiraceae; g_Nitrospira; s_Nitrospira sp900078515	no	no
GCA_002083405.1	Nitrospira sp. SG-bin2	p_Nitrospirota; c_Nitrospiria; o_Nitrospirales; f_Nitrospiraceae; g_Nitrospira; s_Nitrospira sp900078535	no	no
GCA_003576955.1	Nitrospira sp.	p_Nitrospirota; c_Nitrospiria; o_Nitrospirales; f_Nitrospiraceae; g_Nitrospira_A; s_Nitrospira_A sp001567445	no	no
GCA_900696565.1	uncultured Nitrosospira sp.	p_Nitrospirota; c_Nitrospiria; o_Nitrospirales; f_Nitrospiraceae; g_Nitrospira_A; s_Nitrospira_A sp001567445	no	no
GCA_002254325.1	Nitrospira sp. UW-LDO-02	p_Nitrospirota; c_Nitrospiria; o_Nitrospirales; f_Nitrospiraceae; g_Nitrospira_A; s_Nitrospira_A sp900170025	no	no
GCA_002299405.1	Nitrospira sp. UBA667	p_Nitrospirota; c_Nitrospiria; o_Nitrospirales; f_Nitrospiraceae; g_Nitrospira_A; s_Nitrospira_A sp900170025	no	no
GCA_002380995.1	Nitrospira sp. UBA4129	p_Nitrospirota; c_Nitrospiria; o_Nitrospirales; f_Nitrospiraceae; g_Nitrospira_A; s_Nitrospira_A sp900170025	no	no
GCA_002473245.1	Nitrospira sp. UBA7240	p_Nitrospirota; c_Nitrospiria; o_Nitrospirales; f_Nitrospiraceae; g_Nitrospira_A; s_Nitrospira_A sp900170025	no	no
GCA_003456185.1	Nitrospira sp.	p_Nitrospirota; c_Nitrospiria; o_Nitrospirales; f_Nitrospiraceae; g_Nitrospira_A; s_Nitrospira_A sp900170025	no	no
GCA_005116935.1	Nitrospira sp.	p_Nitrospirota; c_Nitrospiria; o_Nitrospirales; f_Nitrospiraceae; g_Nitrospira_A; s_Nitrospira_A sp900170025	no	no
GCA_002435405.1	Nitrospira sp. UBA6488	p_Nitrospirota; c_Nitrospiria; o_Nitrospirales; f_Nitrospiraceae; g_Nitrospira_D; s_Nitrospira_D sp002435325	no	no
GCA_005116865.1	Nitrospira sp.	p_Nitrospirota; c_Nitrospiria; o_Nitrospirales; f_Nitrospiraceae; g_Nitrospira_D; s_Nitrospira_D sp002869855	no	no
GCA_003569385.1	Nitrospirae bacterium	p_Nitrospirota; c_Nitrospiria; o_Nitrospirales; f_Nitrospiraceae; g_Palsa-1315; s_Palsa-1315 sp002737345	no	no
GCA_005788665.1	Nitrospiraceae bacterium	p_Nitrospirota; c_Nitrospiria; o_Nitrospirales; f_Nitrospiraceae; g_Palsa-1315; s_Palsa-1315 sp002737345	no	no
GCA_005793845.1	Nitrospiraceae bacterium	p_Nitrospirota; c_Nitrospiria; o_Nitrospirales; f_Nitrospiraceae; g_Palsa-1315; s_Palsa-1315 sp002737345	no	no
GCA_005798745.1	Nitrospiraceae bacterium	p_Nitrospirota; c_Nitrospiria; o_Nitrospirales; f_Nitrospiraceae; g_Palsa-1315; s_Palsa-1315 sp002737345	no	no
GCA_005116825.1	Nitrospira sp.	p_Nitrospirota; c_Nitrospiria; o_Nitrospirales; f_Nitrospiraceae; g_Palsa-1315; s_Palsa-1315 sp002869885	no	no
GCA_003152135.1	Nitrospira sp.	p_Nitrospirota; c_Nitrospiria; o_Nitrospirales; f_Nitrospiraceae; g_Palsa-1315; s_Palsa-1315 sp003135435	no	no
GCA_001803925.1	Nitrospirae bacterium RIFCSPHIGHO2_02_FULL_42_12	p_Nitrospirota; c_Nitrospiria_A; o_HDB-SIOI813; f_HDB-SIOI813; g_HDB-SIOI813; s_HDB-SIOI813 sp001805205	no	no
GCA_001805025.1	Nitrospirae bacterium GWA2_42_11	p_Nitrospirota; c_Nitrospiria_A; o_HDB-SIOI813; f_HDB-SIOI813; g_HDB-SIOI813; s_HDB-SIOI813 sp001805205	no	no
GCA_001805235.1	Nitrospirae bacterium RIFCSPLOWO2_02_42_7	p_Nitrospirota; c_Nitrospiria_A; o_HDB-SIOI813; f_HDB-SIOI813; g_HDB-SIOI813; s_HDB-SIOI813 sp001805205	no	no
GCA_003477265.1	Nitrospiraceae bacterium	p_Nitrospirota; c_Nitrospiria_A; o_HDB-SIOI813; f_HDB-SIOI813; g_HDB-SIOI813; s_HDB-SIOI813 sp001805205	no	no
GCA_003508715.1	Nitrospiraceae bacterium	p_Nitrospirota; c_Nitrospiria_A; o_HDB-SIOI813; f_HDB-SIOI813; g_HDB-SIOI813; s_HDB-SIOI813 sp001805205	no	no
GCA_002897815.1	bacterium BMS3Abin07	p_Nitrospirota; c_Thermodesulfovibrionia; o_Thermodesulfovibrionales; f_BMS3Bbin05; g_BMS3Bbin05; s_BMS3Bbin05 sp002897855	no	no
GCA_002897875.1	bacterium BMS3Bbin06	p_Nitrospirota; c_Thermodesulfovibrionia; o_Thermodesulfovibrionales; f_JdFR-85; g_BMS3Abin08; s_BMS3Abin08 sp002897935	no	no
GCA_002010755.1	Nitrospirae bacterium JdFR-87	p_Nitrospirota; c_Thermodesulfovibrionia; o_Thermodesulfovibrionales; f_JdFR-88; g_JdFR-88; s_JdFR-88 sp002011795	no	no
GCA_002376155.1	Nitrospiraceae bacterium UBA3562	p_Nitrospirota; c_Thermodesulfovibrionia; o_Thermodesulfovibrionales; f_JdFR-88; g_JdFR-88; s_JdFR-88 sp002011795	no	no
GCA_002376445.1	Nitrospiraceae bacterium UBA3568	p_Nitrospirota; c_Thermodesulfovibrionia; o_Thermodesulfovibrionales; f_JdFR-88; g_JdFR-88; s_JdFR-88 sp002011795	no	no
GCA_002753435.1	Nitrospirae bacterium	p_Nitrospirota; c_Thermodesulfovibrionia; o_Thermodesulfovibrionales; f_Magnetobacteriaceae; g_HCH-1; s_HCH-1 sp001541255	no	no
GCA_002753455.1	Nitrospirae bacterium	p_Nitrospirota; c_Thermodesulfovibrionia; o_Thermodesulfovibrionales; f_Magnetobacteriaceae; g_Magnetobacterium; s_Magnetobacterium casensis	no	no
GCA_001873265.1	Nitrospirae bacterium CG2_30_41_42	p_Nitrospirota; c_Thermodesulfovibrionia; o_Thermodesulfovibrionales; f_SM23-35; g_0-14-3-00-41-53; s_0-14-3-00-41-53 sp002780895	no	no
GCA_002782165.1	Nitrospirae bacterium CG_4_8_14_3_um_filter_41_47	p_Nitrospirota; c_Thermodesulfovibrionia; o_Thermodesulfovibrionales; f_SM23-35; g_0-14-3-00-41-53; s_0-14-3-00-41-53 sp002780895	no	no
GCA_002785245.1	Nitrospirae bacterium CG_4_10_14_0_8_um_filter_41_23	p_Nitrospirota; c_Thermodesulfovibrionia; o_Thermodesulfovibrionales; f_SM23-35; g_0-14-3-00-41-53; s_0-14-3-00-41-53 sp002780895	no	no
GCA_002787195.1	Nitrospirae bacterium CG11_big_fil_rev_8_21_14_0_20_41_14	p_Nitrospirota; c_Thermodesulfovibrionia; o_Thermodesulfovibrionales; f_SM23-35; g_0-14-3-00-41-53; s_0-14-3-00-41-53 sp002780895	no	no
GCA_002790775.1	Nitrospirae bacterium CG_4_9_14_3_um_filter_41_27	p_Nitrospirota; c_Thermodesulfovibrionia; o_Thermodesulfovibrionales; f_SM23-35; g_0-14-3-00-41-53; s_0-14-3-00-41-53 sp002780895	no	no
GCA_001312085.1	Thermodesulfovibrio aggregans JCM 13213	p_Nitrospirota; c_Thermodesulfovibrionia; o_Thermodesulfovibrionales; f_Thermodesulfovibrionaceae; g_Thermodesulfovibrio; s_aggregans	no	yes
GCF_000482825.1	Thermodesulfovibrio islandicus DSM 12570	p_Nitrospirota; c_Thermodesulfovibrionia; o_Thermodesulfovibrionales; f_Thermodesulfovibrionaceae; g_Thermodesulfovibrio; s_yellowstonii	no	yes
GCF_006538445.1	Thermodesulfovibrio sp. Kuro-1	p_Nitrospirota; c_Thermodesulfovibrionia; o_Thermodesulfovibrionales; f_Thermodesulfovibrionaceae; g_Thermodesulfovibrio; s_yellowstonii	no	no
GCA_001803715.1	Nitrospirae bacterium GWD2_44_7	p_Nitrospirota; c_Thermodesulfovibrionia; o_Thermodesulfovibrionales; f_UBA1546; g_UBA1546; s_UBA1546 sp001805105	no	no
GCA_001803945.1	Nitrospirae bacterium RIFOXYA2_FULL_44_9	p_Nitrospirota; c_Thermodesulfovibrionia; o_Thermodesulfovibrionales; f_UBA1546; g_UBA1546; s_UBA1546 sp001805105	no	no
GCA_003500245.1	Nitrospiraceae bacterium	p_Nitrospirota; c_Thermodesulfovibrionia; o_Thermodesulfovibrionales; f_UBA1546; g_UBA1546; s_UBA1546 sp001805105	no	no
GCA_003510545.1	Nitrospiraceae bacterium	p_Nitrospirota; c_Thermodesulfovibrionia; o_Thermodesulfovibrionales; f_UBA1546; g_UBA1546; s_UBA1546 sp001805105	no	no
GCA_002323715.1	Nitrospiraceae bacterium UBA1546	p_Nitrospirota; c_Thermodesulfovibrionia; o_Thermodesulfovibrionales; f_UBA1546; g_UBA1546; s_UBA1546 sp001871685	no	no
GCA_002771505.1	Nitrospirae bacterium CG22	p_Nitrospirota; c_Thermodesulfovibrionia; o_Thermodesulfovibrionales; f_UBA1546; g_UBA1546; s_UBA1546 sp001871685	no	no
GCA_002780905.1	Nitrospirae bacterium CG02_land_8_20_14_3_00_44_33	p_Nitrospirota; c_Thermodesulfovibrionia; o_Thermodesulfovibrionales; f_UBA1546; g_UBA1546; s_UBA1546 sp001871685	no	no
GCA_002781345.1	Nitrospirae bacterium CG01_land_8_20_14_3_00_44_22	p_Nitrospirota; c_Thermodesulfovibrionia; o_Thermodesulfovibrionales; f_UBA1546; g_UBA1546; s_UBA1546 sp001871685	no	no
GCA_002782185.1	Nitrospirae bacterium CG_4_8_14_3_um_filter_44_28	p_Nitrospirota; c_Thermodesulfovibrionia; o_Thermodesulfovibrionales; f_UBA1546; g_UBA1546; s_UBA1546 sp001871685	no	no
GCA_002783085.1	Nitrospirae bacterium CG_4_10_14_3_um_filter_44_29	p_Nitrospirota; c_Thermodesulfovibrionia; o_Thermodesulfovibrionales; f_UBA1546; g_UBA1546; s_UBA1546 sp001871685	no	no
GCA_002790755.1	Nitrospirae bacterium CG_4_9_14_3_um_filter_44_28	p_Nitrospirota; c_Thermodesulfovibrionia; o_Thermodesulfovibrionales; f_UBA1546; g_UBA1546; s_UBA1546 sp001871685	no	no
GCA_003234985.1	Nitrospirae bacterium	p_Nitrospirota; c_Thermodesulfovibrionia; o_Thermodesulfovibrionales; f_UBA6898; g_UBA6898; s_UBA6898 sp003252075	no	no
GCA_003156875.1	Nitrospiraceae bacterium	p_Nitrospirota; c_Thermodesulfovibrionia; o_Thermodesulfovibrionales; f_UBA9935; g_Fen-1308; s_Fen-1308 sp003170655	no	no
GCA_003156895.1	Nitrospiraceae bacterium	p_Nitrospirota; c_Thermodesulfovibrionia; o_Thermodesulfovibrionales; f_UBA9935; g_Fen-1308; s_Fen-1308 sp003170655	no	no
GCA_003157305.1	Nitrospiraceae bacterium	p_Nitrospirota; c_Thermodesulfovibrionia; o_Thermodesulfovibrionales; f_UBA9935; g_Fen-1308; s_Fen-1308 sp003170655	no	no
GCA_003158615.1	Nitrospiraceae bacterium	p_Nitrospirota; c_Thermodesulfovibrionia; o_Thermodesulfovibrionales; f_UBA9935; g_Fen-1308; s_Fen-1308 sp003170655	no	no
GCA_003161685.1	Nitrospiraceae bacterium	p_Nitrospirota; c_Thermodesulfovibrionia; o_Thermodesulfovibrionales; f_UBA9935; g_Fen-1308; s_Fen-1308 sp003170655	no	no
GCA_003162155.1	Nitrospiraceae bacterium	p_Nitrospirota; c_Thermodesulfovibrionia; o_Thermodesulfovibrionales; f_UBA9935; g_Fen-1308; s_Fen-1308 sp003170655	no	no
GCA_003162365.1	Nitrospiraceae bacterium	p_Nitrospirota; c_Thermodesulfovibrionia; o_Thermodesulfovibrionales; f_UBA9935; g_Fen-1308; s_Fen-1308 sp003170655	no	no

ID	NCBI Organism Name	GTDB Taxonomy	GTDB species representative	NCBI type material
GCA_003168405.1	Nitrospiraceae bacterium	p_Nitrospirota; c_Thermodesulfovibrionia; o_Thermodesulfovibrionales; f_UBA9935; g_Fen-1308; s_Fen-1308 sp003170655	no	no
GCA_003168475.1	Nitrospiraceae bacterium	p_Nitrospirota; c_Thermodesulfovibrionia; o_Thermodesulfovibrionales; f_UBA9935; g_Fen-1308; s_Fen-1308 sp003170655	no	no
GCA_003170055.1	Nitrospiraceae bacterium	p_Nitrospirota; c_Thermodesulfovibrionia; o_Thermodesulfovibrionales; f_UBA9935; g_Fen-1308; s_Fen-1308 sp003170655	no	no
GCA_001803605.1	Nitrospirae bacterium GWA2_46_11	p_Nitrospirota; c_Thermodesulfovibrionia; o_Thermodesulfovibrionales; f_UBA9935; g_GWB2-47-37; s_GWB2-47-37 sp001803635	no	no
GCA_003453275.1	Nitrospiraceae bacterium	p_Nitrospirota; c_Thermodesulfovibrionia; o_Thermodesulfovibrionales; f_UBA9935; g_GWB2-47-37; s_GWB2-47-37 sp001803635	no	no
GCA_003538695.1	Nitrospiraceae bacterium	p_Nitrospirota; c_Thermodesulfovibrionia; o_Thermodesulfovibrionales; f_UBA9935; g_GWB2-47-37; s_GWB2-47-37 sp001803635	no	no
GCA_002897755.1	bacterium BMS3Abin10	p_Nitrospirota; c_Thermodesulfovibrionia; o_UBA6902; f_BMS3Bbin08; g_BMS3Bbin08; s_BMS3Bbin08 sp002897775	no	no
GCA_002898055.1	bacterium BMS3Abin09	p_Nitrospirota; c_Thermodesulfovibrionia; o_UBA6902; f_UBA6902; g_BMS3Abin09; s_BMS3Abin09 sp002897915	no	no
GCA_002451095.1	Nitrospiraceae bacterium UBA6907	p_Nitrospirota; c_Thermodesulfovibrionia; o_UBA6902; f_UBA6902; g_UBA6902; s_UBA6902 sp002451135	no	no
GCA_003508235.1	Nitrospiraceae bacterium	p_Nitrospirota; c_Thermodesulfovibrionia; o_UBA6902; f_UBA6902; g_UBA6902; s_UBA6902 sp002451135	no	no
GCA_001803725.1	Nitrospirae bacterium GWD2_57_8	p_Nitrospirota; c_UBA9217; o_UBA9217; f_UBA9217; g_GWC2-57-13; s_GWC2-57-13 sp001805055	no	no
GCA_003483085.1	Nitrospiraceae bacterium	p_Nitrospirota; c_UBA9217; o_UBA9217; f_UBA9217; g_GWC2-57-13; s_GWC2-57-13 sp001805055	no	no
